# Supplementary material for: What happens when pharmacist independent prescribers lead on medicine management in older people’s care homes: a qualitative study
Source: BMJ Open. 2023 Oct 31;13(10):e068678. doi: 10.1136/bmjopen-2022-068678 (PMC10619113; doi:10.1136/bmjopen-2022-068678)
Supplement: Supplementary data [file bmjopen-2022-068678supp003.pdf]

Supplementary file 3 Coding Framework

code	Number participants	Number times coded
1 Implementation	10	30
list of services offered by PIP	5	7
care home study procedure	8	23
GP study procedures	6	7
2 Mechanisms of impact	7	46
medication changes made	13	33
bio chemical monitoring	8	9
non-patient facing activities	8	25
completing PCP	5	15
tasking items to GP	4	4
liaising with community pharmacist about scripts	7	14
liaising with primary care practice pharmacy staff	4	6
evidence of GP tasking items to PIP	4	10
recruiting residents	3	4
authorising repeat prescription	7	13
staff education	8	13
staff training	12	15
review of medication systems	11	18
review of stock	7	8
care home workload due to intervention	7	24
3 Communication	10	62
PIP to GP	9	19
PIP to care home	12	55
PIP to residents and relatives	8	16
PIP to others	10	21
GP to PIP	7	16
Care home to PIP	13	56
resident or relative to PIP	0	0
GP to CH	5	6
4 Outcomes	10	46
case studies of improvement to residents	18	43
case studies where planned change not successful	2	2
reduced drug or stock wastage	3	3
improved ordering system	8	19
adding to multi disciplinary practice	17	44
opinion on intervention	6	32

PIP satisfaction or not	5	11
GP satisfaction or not	7	28
Care home satisfaction or not	10	47
5 intervention safety	4	6
PIP perspective	5	12
GP perspective	7	17
Care home perspective	6	9
resident or relative perspective	0	0
6 contextual factors	13	61
barriers to delivery	18	44
facilitators to delivery	14	42
impact of site factors on delivery	14	34
impact of patient factors on delivery	8	12
GP workload impact	12	27
care home staff awareness	11	30
7 Intervention normalised into routine	13	25
actions taken to ensure intervention works	3	5
narrative of engagement or disengagement	10	18
legacy of intervention	11	20
Future developments	2	4
8 Discussion on professional roles	12	46
9. Geographic differences	6	9
10 Should this type of service be implemented	14	21
