# Supplementary material for: What happens when pharmacist independent prescribers lead on medicine management in older people’s care homes: a qualitative study
Source: BMJ Open. 2023 Oct 31;13(10):e068678. doi: 10.1136/bmjopen-2022-068678 (PMC10619113; doi:10.1136/bmjopen-2022-068678)
Supplement: Supplementary data [file bmjopen-2022-068678supp004.pdf]

Supplementary file 4 Example of text to code 'PIP satisfaction or not'

PIP 1

Reference 1-the one to one contact, the face to face umm for those patients that were able to participate umm you know I think a lot of the time they are happy to see somebody and know there is another person involved in their care, umm a lot of them were great characters and you know it was lovely for building that relationship and then of course there would be the patients where you would be in a relationship with them and then you would be leaving the room and they had forgotten who you were, that was just their you know their conditions, so umm but no it was a positive experience definitely from the patients' perspective and getting to know them.

Reference 2 - I thought the project is really worthwhile umm and I hope that you are able to get all the data and make sense of it all especially with me, umm I, you know I think it's a great project, I have encouraged other people to get involved in it locally umm and you know I know there's a few. -

PIP 2

Reference 1 - part of me, admittedly four hours isn't enough time, sometimes I wish it was more patients because I felt I could have probably made more of an impact and I do wish it was only the one Care Home but other than that.

Reference 2 – I think so with the Primary Care network role, pharmacist's role that is something that each Care Home resident will be getting and should be getting the Pharmacist's review so. Yeah it is, it's utterly definitely of benefit. - §

PIP 6

Reference 1 – Q I'm just wondering if there is a specific example of where you felt that that lack of knowing affected your sort of practice? Response I think it just was the, I think it was just the fact that I never got to the stage where the Home were calling me about things, we never got to that stage

Reference 2 – Q Was there anything that was particularly good about your experience of delivering the service? Response 2 / 3 Definitely, yes, I have been very negative, no I think just the fact that there is a need for it, you know I did pick up on things in review, GPs now are so busy you know particularly when they get given, the way that it happens, that they get given a whole Care Home to look after, they do tend to when, you know once that patient moves into that Care Home the GP will you know do a quick review and all of that to get to know them and their records a bit better but often after that the GP can only ever respond to acute needs and those patients, you know often those, the way it works at the Care Home I was at, that the GP would visit every Thursday, they would have a list of all of the patients that the Care Home wanted them to see so it was often dictated by what the Care Home needs were rather than what the GP, and by that time it was all filled up so the GP you know would follow up on some things but in terms of on-going chronic conditions that's where I feel Pharmacists in Care Homes have an impact and that I saw, so my main things were about trying to reduce pain medication, so we got a few people reduced on some of the Opioid patches and monitoring, not over-monitoring because you know that is totally unnecessary at this stage but the monitoring that is necessary so there was some blood tests that had slipped that were important and de-prescribing other things for different medications like statins that patients don't need so much anymore and the GP I felt was grateful for that because they would love to have

the time to do that I'm sure, but yeah they are working on a, you know firefighting basis whereas this was more of a you know a pro-active approach to it. –

PIP 19

Reference 1 - it was generally positive, there were obviously some things we will perhaps come back to that were sort of challenging in terms of time management and prioritising what I needed to do umm but umm but yes on the whole I think I learnt from it and it was beneficial to the patients I hope. –

PIP 9

Reference 1 - quite good really it kind of built upon the kind of relationships that I had with the Care Home anyway, so I do speak to them quite often on the phone, I deal with queries but it just, it made things better because I had to go and see the Home, I didn't know where the Home was, it was just out there somewhere. I didn't know what the Home was like, I had a picture of it in my head about who the people were who I was dealing with but I didn't know them so I had to go and see them so that was great and then because we knew one another we then kind of shared email addresses and stuff and we made maybe some of the things a bit more twenty-first century, we were emailing one another whereas in the past they were faxing us queries and stuff so, so that's yeah so it was, yeah it was good it kind of pushed us a bit more together to kind of work together.

Reference 2 - can't think of anywhere where it was negative, I can just see it was all positive. Basically we did do a kind of an in-depth six month review of people which I don't think we would have done it had I not been involved in the CHIPPS study you know and there was lots of, because basically what I used it for we kind of took a step back and said 'why is this patient on this drug?' as opposed to 'what's the drug for?' so if they are on drugs and antidepressants you know well anti-depressants they're depressed, but actually what I started doing was going you know 'why were they put on this drug?' and sometimes we didn't know and they were on the drug and I didn't think it was doing any good and I said 'maybe we could maybe halve this or stop this?' so yeah so I can't think of anything, I can't think of anything negative.

Reference 3 - So communication only improved, you know we did move away from them sending faxes to us saying 'and you owe these medicines for various people' to them emailing and you know yeah and it just improved you know and sometimes when I had those emails I was able to reply back to them and say 'I've sorted it out for you now' but I was able to reply to the Pharmacy as well saying 'these things have been sorted, the script is on the way' or whatever and it only improved really, do you know what I mean 'improved'?

Reference 4 - I thought whatever you are going to try and show a difference in, that half my time or double my time was taken filling out paperwork when actually I did do it in half the time but you do not know and I kind of squandered some of that yeah
