# Supplementary material for: What happens when pharmacist independent prescribers lead on medicine management in older people’s care homes: a qualitative study
Source: BMJ Open. 2023 Oct 31;13(10):e068678. doi: 10.1136/bmjopen-2022-068678 (PMC10619113; doi:10.1136/bmjopen-2022-068678)
Supplement: Supplementary data [file bmjopen-2022-068678supp005.pdf]

Table Demographic characteristics of interview participants

Intervention ID Interviewee N=38		General practice			Pharmacist			Care Home		
**Triad**	**Interviewee**	**Location***	**Patient list**	**Previously employed PIP**	**Time Independent Prescriber**	**Previous experience in care homes**	**Number of residents in intervention**	**Type of registration**	**Indices of multiple deprivation***	**Ownership**
**1**	PIP, CH Manager	Northern Ireland Urban	< 10,000	Yes	1 month	No	6	Dual	10	Private
**2**	PIP	England Semirural	≥10,000	Yes	2 months	Yes	20	Dual	8	Private
**3**	GP	England Urban	< 10,000	No	12 months	Yes	16	Residential	6	Local Authority
**4**	PIP	Scotland Urban	≥10,000	No	12 months	No	24	Dual	10	Private
**6**	PIP, GP, CH Manager, CH Staff	England Urban	≥10,000	No	16 months	No	22	Residential	5	Voluntary
**8**	PIP, GP, CH Manager	England Semirural	≥10,000	Yes	18 months	Yes	24	Dual	4	Private
**9**	PIP, CH Manager	England Rural	≥10,000	Yes	18 months	No	21	Residential	6	Private
**11**	PIP, GP, CH Staff	Scotland Semirural	< 10,000	Yes	3 years	No	14	Residential	8	Private
**12**	CH Manager	Northern Ireland Urban	≥10,000	Yes	4 years	Yes	19	Residential	7	Voluntary
**14**	PIP, GP, CH Manager (x2), CH Staff	Scotland Rural	< 10,000	Yes	4 years	No	20	Dual	9	Private
**15**	PIP	Scotland Urban	< 10,000	Yes	6 years	Missing	18	Dual	7	Private
**16**	PIP, GP	England Urban	< 10,000	No	6 years	Yes	20	Dual	1	Private
**17**	PIP	Northern Ireland	Missing	Yes	7 years	Yes	6	Dual	10	Private

		Urban								
**18**	CH Manager	Scotland Urban	< 10,000	Yes	8 years	No	9	Dual	6	Private
**19**	PIP, GP, CH Staff	England Rural	< 10,000	No	9 years	Yes	24	Residential	6	Private
**20**	PIP	Northern Ireland Semirural	Missing	Yes	10 years	Yes	23	Dual	8	Private
**21**	GP, CH Manager (x2), CH Staff	Scotland Urban	< 10,000	Yes	14 years	Yes	21	Dual	10	
**22**	PIP	Scotland Urban	< 10,000	Yes	16 years	Yes	11	Dual	Private	

Key

Location country: urban refers to city GP practice, semirural refers to market town GP practice, rural refers to village GP practice

Indices of Multiple Deprivation: The deciles ranks are nationally calculated by ranking the 32,844 LSOAs in England from most deprived to least deprived and dividing them into 10 equal groups. LSOAs in decile 1 fall within the most deprived 10% of LSOAs nationally and LSOAs in decile 10 fall within the least deprived 10% of LSOAs nationally. NI decile calculated manually from rank
