# Supplementary material for: What happens when pharmacist independent prescribers lead on medicine management in older people’s care homes: a qualitative study
Source: BMJ Open. 2023 Oct 31;13(10):e068678. doi: 10.1136/bmjopen-2022-068678 (PMC10619113; doi:10.1136/bmjopen-2022-068678)
Supplement: Author's manuscript [file bmjopen-2022-068678.draft_revisions.pdf]

BMJ Open is committed to open peer review. As part of this commitment we make the peer review history of every article we publish publicly available.

When an article is published we post the peer reviewers' comments and the authors' responses online. We also post the versions of the paper that were used during peer review. These are the versions that the peer review comments apply to.

The versions of the paper that follow are the versions that were submitted during the peer review process. They are not the versions of record or the final published versions. They should not be cited or distributed as the published version of this manuscript.

BMJ Open is an open access journal and the full, final, typeset and author-corrected version of record of the manuscript is available on our site with no access controls, subscription charges or pay-per-view fees (http://bmjopen.bmj.com).

If you have any questions on BMJ Open's open peer review process please email info.bmjopen@bmj.com

BMJ Open

A qualitative understanding of outcomes when pharmacist independent prescribers lead on medicine management in older people's care homes.

Journal:	*BMJ Open*
Manuscript ID	bmjopen-2022-068678
Article Type:	Original research
Date Submitted by the Author:	27-Sep-2022

Complete List of Authors:	Birt, Linda; University of East Anglia; University of Leicester<br>Dalgarno, Lindsay; University of Glasgow<br>Poland, Fiona; University of East Anglia, School of Health Sciences<br>Wright, David; University of East Anglia; University of Leicester<br>Bond, Christine; University of Aberdeen

Keywords:	PRIMARY CARE, CLINICAL PHARMACOLOGY, GERIATRIC MEDICINE, Risk management < HEALTH SERVICES ADMINISTRATION & MANAGEMENT, QUALITATIVE RESEARCH

SCHOLARONE™
Manuscripts

I, the Submitting Author has the right to grant and does grant on behalf of all authors of the Work (as defined in the below author licence), an exclusive licence and/or a non-exclusive licence for contributions from authors who are: i) UK Crown employees; ii) where BMJ has agreed a CC-BY licence shall apply, and/or iii) in accordance with the terms applicable for US Federal Government officers or employees acting as part of their official duties; on a worldwide, perpetual, irrevocable, royalty-free basis to BMJ Publishing Group Ltd ("BMJ") its licensees and where the relevant Journal is co-owned by BMJ to the co-owners of the Journal, to publish the Work in this journal and any other BMJ products and to exploit all rights, as set out in our licence.

The Submitting Author accepts and understands that any supply made under these terms is made by BMJ to the Submitting Author unless you are acting as an employee on behalf of your employer or a postgraduate student of an affiliated institution which is paying any applicable article publishing charge ("APC") for Open Access articles. Where the Submitting Author wishes to make the Work available on an Open Access basis (and intends to pay the relevant APC), the terms of reuse of such Open Access shall be governed by a Creative Commons licence – details of these licences and which Creative Commons licence will apply to this Work are set out in our licence referred to above.

Other than as permitted in any relevant BMJ Author's Self Archiving Policies, I confirm this Work has not been accepted for publication elsewhere, is not being considered for publication elsewhere and does not duplicate material already published. I confirm all authors consent to publication of this Work and authorise the granting of this licence.

For peer review only

A qualitative understanding of outcomes when pharmacist independent prescribers lead on medicine management in older people's care homes.

Linda Birt[1,2], Lindsay Dalgarno,[3,4] Fiona Poland,[1] David Wright,[5,2] Christine Bond[3]

1.School of Health Sciences, University of East Anglia

2.School of Healthcare, University of Leicester

3.Institute of Applied Health Sciences, University of Aberdeen, Aberdeen, UK School of HealthCare, University of Leicester

4.General Practice and Primary Care, University of Glasgow

5. School of Pharmacy, University of East Anglia

Corresponding author

Dr Linda Birt

School of Health Sciences

Queen's Building

University of East Anglia

Norwich Research park

Norwich

NR4 7TJ

Linda.birt@uea.ac.uk

For peer review only

A qualitative understanding of outcomes when pharmacist independent prescribers lead on medicine management in older people's care homes.

Abstract

Objective: Residents in care homes for older people frequently experience polypharmacy, increasing the likelihood of adverse drug events. Pharmacists working within a multidisciplinary primary care team are ideally placed to lead on medication reviews. A randomised controlled trial integrated pharmacists, with independent prescribing rights (PIPs), into older people care homes. In the intervention service PIPS worked with general practitioners (GPs) and care home staff for 6 months, to optimise medication management at individual resident and care home levels. PIPs activity included stopping medicines that were no longer needed or where potential harms outweighed benefits. This analysis of qualitative data examined stakeholders perceptions of how the service impacted on care home medication procedures and resident wellbeing.

Design: Pragmatic research design with secondary analysis of interviews

Settings: Primary care pharmacist intervention in older people care homes in England, Scotland and Northern Ireland

Participants: Recruited from intervention arm of the trial: Independent prescribing pharmacists (n=14), General Practitioners (n=8) Care home managers (n=9) Care home Staff (n=6)

Results: There were resonances between different participant groups about the potential benefits to care home residents of a medication service provided by PIPs. There were small differences in perceptions about outcomes related to communication between professionals. Results are reported through three themes 1) *It's a natural fit'* – pharmacists undertaking medicine review in care homes fitted within multidisciplinary care; 2) *'The resident is cared for'* - there were subjective improvements in residents' wellbeing; 3) *Moving from 'firefighting' to effective systems* - there was evidence of changes to care home medicine procedures.

Conclusions: This study suggests that pharmacist independent prescribers in primary care may be the healthcare professional optimally placed to be the medicines link between general practitioners and care home staff

Strengths and limitations of this study

- The study draws on data from a relevant sample of 37 healthcare professionals involved in medication management in care homes

For peer review only

- The analytical approach foregrounded the effect of pharmacist-led medication review on the older resident in care homes, therefore having relevance to practices supporting safer medicines in this group of people.
- A limitation is the absence of residents' voices, due to recruitment challenges with this group
- The study provides insights into activity provided by specialist pharmacists with independent prescribing qualifications so it cannot be assumed that more generalist pharmacist would have the experience to make specialised medicine management decisions.

Key words Pharmacist prescriber, care home, older people, primary care, medication, general practice, multidisciplinary health care,

For peer review only

Introduction

Older people in care homes are often subject to complex medication regimes which may include the concurrent administration of more than five different medicines daily (1,2). This is known as polypharmacy; it is associated with a higher risk of adverse side effects including falls, hospitalization and mortality (3,4) and the complexity of managing medicines and administering multiple drugs increase care home staff workload (5). This can lead to increased rates of medicine administration errors, and while few errors result in serious consequences for residents, all need to be reported and followed-up (6). The Care Quality Commission highlights management of medicines as an area of concern and holds it under constant review (7). In United Kingdom care homes, prescribing and structured medicine reviews for individual residents are the responsibility of health professionals in the primary care team (8). Patient medicines review has historically been the role of the general practitioner, other multidisciplinary team members are increasingly undertaking reviews (8). Pharmacists are being routinely employed to lead on medicine review in primary care practices, and an English government initiative to enable pharmacists to have a role within care homes is gathering momentum (9). If pharmacists have the postgraduate Independent Prescriber qualification, they can start (prescribe) or stop (deprescribe) medicines within their area of clinical competence (10). This enhanced role allows them to use their expert clinical knowledge of medicines to deliver medication management services in care homes (11).

This paper reports on a secondary analysis of interview data collected from pharmacists with independent prescribing rights (PIPs), general practitioners (GPs) and care home (CH) staff as part of the process evaluation undertaken As part of the Care Home Independent Prescribing Pharmacist Study (CHIPPS). Within the trial a service intervention was designed which enabled pharmacist-led medication management for care homes. PIPs worked within care homes on a weekly basis over 6 months to review their medication systems and residents' personal medicines (12). PIPs were based within the general practice aligned to the care home. The secondary analysis aimed to understand how the PIP role was enacted and PIPs, GPs and CH staffs' perceptions of how PIP activity impacted on care home medication procedures and resident wellbeing, concepts which were outside the remit of the process evaluation (13). The pharmacist-led service offered in care homes is briefly described below to set the findings in the practice context; full details of the trial design are available at (12).

The pharmacist-led service was delivered through a triad of GP, PIP based at the GP practice, and up to 24 care home residents, from up to three care homes, registered with the GP. Service specifications were developed following focus groups with stakeholders (14) to guide the PIPs work, see supplementary file 1. The primary trial outcome was reduction in falls for residents; however in a pre-study focus group health care professionals (HCPs) wondered whether there might be wider benefits for residents. The communication between trial participants, their understanding of responsibilities of PIPs and outcomes for residents are the focus of this secondary analysis of process evaluation interview data. Within the trial 25 triads were recruited to the intervention arm. Sixteen

For peer review only

PIPs were already working with the GP; the remaining nine were allocated a GP practice for the purposes of the study. PIPs undertook a two-day bespoke face-to-face training programme, produced a portfolio of evidence of competencies and finally were signed off to prescribe in older people medicine by an independent GP (15). To spread researcher workload (triad recruitment, data collection) the intervention was delivered over four six-month phases between January 2018 and March 2020. Data collection for the process evaluation occurred between May 2019 and March 2020 as each phase completed. The secondary analysis of interview data was carried out by (LB) and (LD).

Methods

Design

Pragmatism is a research methodology suited to characterising knowledge about how behaviours and actions impact on healthcare systems and outcomes. This approach can help appreciate the value of knowledge within its context of activity, uses and how these relate to the experience of addressing practical problems (16). As this work aimed to understand and learn from the diverse ways different stakeholders engaged with and perceived the intervention, a pragmatic design provided a way to recognise multiple realities of people involved. English ethical approval was gained from East of England Cambridge Central Research Ethics Committee 17/EE/0360 (28.11.2017; this applied to research in Northern Ireland. Scottish ethical approval was gained from Scotland A research Ethics Committee 17/SS/0118 (07.12.2017). Written informed consent was obtained from all subjects.

Recruitment and sample

At the end of each 6-month intervention all stakeholders in each triad (PIP n=23 [two withdrew during the study], GP n=25, CH managers n=38) were invited by email to take part in a semi-structured interview. A reminder email was sent after two weeks. CH managers provided recruitment information staff, residents and relatives. Those interested in participating returned expressions of interest to study researchers. A purposive sampling framework was designed to ensure representation across stakeholder roles, location and phases of the intervention. However, response rates were low and therefore all those replying were invited to interview.

Data collection

A semi-structured interview guide drew on outcomes from the earlier feasibility study (17) and reflected the Medical Research Council process evaluation domains (18). Questions explored participants' experiences of the service intervention, anticipated and unanticipated outcomes including multidisciplinary communication and outcomes for residents. Interviews were undertaken by (LB) and (LD) academic researchers, between May 2019 and March 2020, either in person or by

For peer review only

telephone and audio recorded and professionally transcribed. Informed consent was obtained before interview.

Data management and analysis

An inductive thematic analysis was used to characterise participants' experiences of delivering or receiving the pharmacist-led service. Thematic analysis provides a structured, methodical way for the researcher to familiarise with the data, and to organise, analyse and report findings; importantly it is not strongly aligned to any epistemological stance (19) so was appropriate within the pragmatic design. Both researchers familiarised themselves with the data through checking transcripts for accuracy, then reading and making analytical memos (20). Next, they independently developed open codes which were discussed with the wider research team to refine the boundaries of the codes. Each interview was coded and any uncertainty arising during coding was discussed between researchers. While the researchers predominantly coded interviews they had carried out, each coded two of the other's interviews to check for consistency of approach. This along with fortnightly review meetings increased dependability during analysis. The coding framework was revised iteratively as analyses progressed, for example grouping similar codes into a category of meaning. Categories were refined and, following discussions with the research team and patient and public partners, the final themes (overarching, abstracted semantic interpretations of the data (21) were developed. While a theme does not rely on specific quantities of data, here each theme provided extensive examples of data. To further support trustworthiness in results, illustrative examples of data within the theme were shared at research meetings to seek consensus from healthcare professionals, including pharmacists and general practitioners. Participant validation was not undertaken due to the time between interviews conducted in each phase and the end of study analysis.

Patient and Public Involvement

Public involvement was supported by the Patient and Public Involvement in Research group (further details at https://nspccro.nihr.ac.uk/working-with-us/public-patient-and-carer-voice-in-research). Public members were involved at all stages of the trial and in relation to this paper they reviewed data and provided comments on emerging interpretations. These enabled the research team to have confidence in their interpretations and to consider in greater detail the resident focused benefits.

Findings

Interviews, lasting between 30 and 90 minutes, were undertaken with 38 HCP: 14 PIPs, 9 GPs, 9 CH Managers and 6 CH staff. Participants were recruited from 18 triads: six in Scotland, 4 in Northern Ireland and 8 in England. There was no expression of interest from any stakeholder in seven triads, in two the PIP had not delivered the intervention; nonetheless data was collected from a heterogenous sample of professionals and locations as shown in Table 1 (demographic characteristics of interview participants). We identify illustrative quotes with professional role and unique triad number; non italicised text in quotes is for explanation.

For peer review only

Table 1 Demographic characteristics of interview participants

Intervention ID Interviewee N=38		General practice			Pharmacist			Care Home	
**Triad**	**Interviewee**	**Location**	**Patient list**	**Previously employed PIP**	**Time Independent prescriber**	**Previous experience in care homes**	**Number of residents in intervention**	**Type of registration**	**Ownership**
**1**	PIP, CH Manager	Northern Ireland	< 10,000	Yes	1 month	No	6	Dual	Private
**2**	PIP	England	≥10,000	Yes	2 months	Yes	20	Dual	Private
**3**	GP	England	< 10,000	No	12 months	Yes	16	Residential	Local Authority
**4**	PIP	Scotland	≥10,000	No	12 months	No	24	Dual	Private
**6**	PIP, GP, CH Manager CH Staff	England	≥10,000	NO	16 months	No	22	Residential	Voluntary
**8**	PIP, GP, CH Manager	England	≥10,000	Yes	18 months	Yes	24	Dual	Private
**9**	PIP CH Manager	England	≥10,000	Yes	18 months	No	21	Residential	Private
**11**	PIP, GP, CH Staff	Scotland	< 10,000	Yes	3 years	No	14	Residential	Private
**12**	CH manager	Northern Ireland	≥10,000	Yes	4 years	Yes	19	Residential	Voluntary
**14**	PIP, GP, CH Manager(x2) CH Staff	Scotland	< 10,000	Yes	4 years	No	20	Dual	Private
**15**	PIP	Scotland	< 10,000	Yes	6 years	Missing	18	Dual	Private
**16**	PIP, GP	England	< 10,000	No	6 years	Yes	20	Dual	Private
**17**	PIP	Northern Ireland	Missing	Yes	7 years	Yes	6	Dual	Private
**18**	CH manager	Scotland	< 10,000	Yes	8 years	No	9	Dual	Private

For peer review only

**19**	PIP, GP, CH Staff	England	< 10,000	No	9 years	Yes	24	Residential	Private
**20**	PIP	Northern Ireland	Missing	Yes	10 years	Yes	23	Dual	Private
**21**	GP, CH Manager(x2) CH Staff	Scotland	< 10,000	Yes	14 years	Yes	21	Dual	Private
**22**	PIP	Scotland	< 10,000	Yes	16 years	Yes	11	Dual	Private

For peer review only

Three themes were developed. Theme 1) *It's a natural fit' -multidisciplinary working in care homes* explores GP, PIP and care home staff experience of this new role; 2) *'The resident is cared for' - shared goals in medication management* provides exemplars of improvement in residents experience of medication and the impact of targeted deprescribing; 3) *Moving from 'firefighting' to effective systems* reports on the improvement to organisation systems across care homes and GP practices and whether this lasted beyond the intervention. Participants mostly recounted positive experiences but some identified challenges inherent in pharmacist-led medication management in care homes.

Theme 1' It's a natural fit'- multidisciplinary working in care homes

This theme reports GP, care home staff and PIP experiences of pharmacist-led medication management. The GP and care home staff valued the PIP activity for differing reasons, suggesting a PIP role can meet both sets of needs.

GP perceptions

GPs highlighted the safety advantage of having PIPs within the multidisciplinary team explaining that it was helpful to have a 'another pair of eyes' to increase patient safety:

> *Having a pharmacist who had good knowledge of all kinds of medications, going through polypharmacy, with a fine-tooth comb and picking up any errors or things we could do better.* GP_21

GPs also drew on PIP expertise for the wider benefit of care home residents questioning *'things that can be crushed or can be opened or whether they need to be changed to suspensions'*, explaining the PIP '*seems to know the answer for everything with regards to medication interactions and probably the thing I ask her most is appliances, different catheters and things*' GP_8.

GPs explained that as the PIP could spend several hours within the home with a small number of residents, they could understand residents' medication needs in greater detail, *'it has brought up things that I might not have thought about'* GP_16. GPs and care home staff were generally open to suggestions from PIPs. However, two PIPs not based in the GP practice prior to the intervention struggled to have their prescribing role accepted, *'I couldn't just go off and prescribe things because the doctor would need to know … it was quite obvious she didn't want me to do anything without putting it through her'* PIP_19. This may suggest a need for actively reviewing and clarifying accountability of roles so that PIPs may be fully integrated into the multidisciplinary team.

While GPs found it hard to quantify the impact of the intervention on their workload, on reflection they mostly described how they were now dealing with fewer daily enquiries from care homes. Where the PIP and GP had established communication channels, the PIP regularly identified residents and issues that the GP should look at in their weekly ward round:

> *It has reduced the time taken to see patients as I can be confident their meds are all up to date, tests required for routine monitoring have been flagged up and I have been able to action these.* GP_3

One GP described a situation where they believed a PIP had increased their workload as the PIP had requested biochemical monitoring which was '*creating work…actual fact I didn't change resident's dose, there was no indication to do so and I did wonder what I was trying to achieve.'* GP_19. In part this situation arose as the PIP did not have full access to the resident's clinical records and was unaware a blood test had been taken during a recent hospital visit, highlighting the importance of PIPs being able to have full access to the primary care team's systems and patient records.

Care home team perceptions

Care home managers and staff identified benefits in having a dedicated pharmacist attached to the home. Most care home teams explained they had gained improved accessibility and advice by having the PIP as point of contact in the primary care team, *'if you're phoning a surgery about a pharmacy issue, probably better speaking to a pharmacist than a GP'* CH manager_21a.

Where multidisciplinary working was embedded care home teams explained the clinical skills of each professional were respected, and teamwork supported effective resident care:

> *The* [CH] *nurses' assessments are being taken as a valuable tool, because the nurses are observing, the nurses are giving the assessment, and the nurses are liaising with the PIP, to prescribe what they think is needed, so I think it's a win, win situation, the nurses are feeling valued, and the PIP as well.* CH manager_21b

However, in two homes the PIP was not able to become integrated into the care home. In one the PIP reported that care home staff were unclear of the PIP role. In another there was confusion about the legal position of the PIP to prescribe and the manager insisted the GP signed off all PIP prescribing activity. This highlights the importance of understanding and trust across the triad.

PIP perceptions

PIPs commonly reported that their prescribing authority meant decisions and residents care could be followed up in a timely way:

> *I think the benefits of having pharmacists in Primary Care is we can go in and make the changes and own it and follow it up rather than pass it on, …leaving it for other people to follow up.* PIP_17

A few PIPs mentioned the role of the pharmacy technician as potentially supporting their work, as pharmacy technicians would undertake stock control and check medicine administration records for accuracy with stock. Only one PIP appeared to have worked with a technician during the intervention '*…a [pharmacy] technician that's employed to go around the homes, I had a relationship with them already, … a lot of the work that they had already done, …so it meant me going in wasn't*

For peer review only

as huge job to turn around the whole care home' PIP_14. A GP pointed out that it may not be good use of a pharmacist '*to be going in and counting tablets and working out what the home needed'* GP_21.

In summary GPs and PIPs made clear what expertise an independent prescribing pharmacist could bring to the multidisciplinary team and saw a continuing place for pharmacists in care homes. While most care home teams valued the PIP role a few were still uncertain on the range of their specific responsibilities.

Theme 2 'The resident is cared for' - shared goals in medicine management

GPs and PIPs highlighted a strength of having the PIP as 'the linchpin in medicine management' in that they had detailed pharmaceutical knowledge allowing them to take actions which would directly and specifically benefit residents. PIPs reviewed prescriptions and could actively seek to rectify any prescribing errors and reduce the drug burden related to adverse effects from medicines. By doing this they importantly improved safety for the resident and helped to make MAR (Medication administration record) sheets fit for purpose thereby potentially reducing the risk of administration error. Changes the PIP could make ranged from small actions such as changing the time of eye-drop administration, so the resident was not woken every night by staff, and changing tablet preparations to soluble form, to complex titrating of drugs, for example, reducing doses of morphine-based painkillers and stopping antipsychotic drugs.

PIPs reviewed all medication in the context of a resident's current biomedical markers and stage of life. Residents, like others in the multidisciplinary team, were sometimes uncertain of the PIP's credentials, and PIPs reported that a few residents were reluctant to have their medicines reviewed preferring to remain with prescriptions given by the GP or secondary care team. There were a couple of triads where care home teams struggled to recall changes which had impacted on residents' wellbeing. These proved to be either homes where the PIP struggled to build working relationships and therefore their medication activity was negligible, or homes that already had highly developed relationships with either the PIP allocated to the intervention or with another pharmacist. In this latter case it was reported that most residents already received regular medication reviews.

Managing resident prescriptions

Independent prescribing activity was a key role of the PIP in the multidisciplinary team which could directly benefit residents, often because this could process a new prescription much more speedily:

> *If my residents are feeling their symptoms, then refer to the PIP, and the PIP will prescribe immediately, there will immediate response, and then the residents will be happy.*
> CH manager_21b

Other care home teams identified how having direct contact with a PIP stopped conditions worsening, '*somebody having dry skin, and being able to contact a pharmacist, definitely improved care and stopped things becoming more acute, skin breaking down'* CH Manager_16. They explained

that for such a minor complaint they would not have contacted the GP but would have waited for the weekly GP visit, with the ensuing delay potentially leading to the condition becoming acute.

Key to successful deprescribing, reducing or stopping a medicine, was the PIP having trustful relationships with the care home team, the GP, the resident or their family. The importance of this trustful relationship was made evident when care home teams explained how they were now confident in trying to titrate prescriptions down as they knew the PIP was easy to contact and had agreed that if there were adverse effects the process could be stopped, and the medicine reinstated.

> *Seeing PIP on a weekly basis meant we were able to make some huge reductions but also we were both very honest with each other so when we had got rid of almost everything and then resident's behaviour got worse again, we were able to add in a tiny little bit of something and I mean tiny bit which was appropriate*. CH Manager_9

Titrating anti-psychotic medication is a complex process and each stakeholder needs to be fully committed and in good communication with each other. Here the PIP explains how the process continued after the intervention ended, illustrating the continued benefit of having a PIP working in the GP practice:

> *We had started to titrate it down and for a few days they were okay and then they started to need a lot of diazepam so we increased it back up but now it has come back down by half; they are checking and watching even though the study has ended because I have been involved and generally keeping an eye on things too they still have my contacts. PIP_1*

Discussions on deprescribing were also enabled between PIP and the resident or their relative, thereby providing more patient-centred care. A care home manager explains:

> '*…talking through that with the PIP and the resident she has come to a circle and is off her Butec patches with no pain; that was agreed with her and her family … she didn't mind coming off and giving it a trial period.* CH Manager_6

Shared decisions

While it was intended that PIPs would be actively engaged with residents as part of the intervention, frequently residents did not have the mental capacity for this. However, even though a PIP stated that residents would possibly have little recall of their conversation they still found it relationally valuable to meet with residents '*I enjoyed meeting them all and then they become more than just a name on a page, you remember the person behind the medication*' PIP_1. A few PIPs also reported the benefit of engaging with family:

> *I could really get on well with the family, she wanted to know everything what I was going to do so that was more interesting because I could discuss things, a really good example of joint working with the family.* PIP_16

In summary most participants readily identified ways in which the intervention had directly benefited individual residents. The key factor enabling PIPs to work in ways which benefited residents stemmed from the trust the care home team, resident and their family could place on the PIP as a readily accessible, clinically competent person who could react appropriately if adverse consequences arose to any medicine change.

Theme 3 Moving from firefighting to effective systems

Within the care home, most of the PIPs actively reviewed and where appropriate improved medication systems, for example streamlining dispensing systems, consolidating MAR charts and stock control.

Streamlining medicine supply and stock control

PIPs could articulate their detailed knowledge of the role of the community pharmacist and the processes which may be in place when a medicine is started or stopped, particularly the time it can take for automated systems to register the change. This 'insider' knowledge' placed the PIPs in an ideal position to educate other professionals and try to streamline medicine ordering processes:

> *Geriatrician didn't realise that although it was stopped in the MAR chart the next month's is out there in the van waiting to be delivered, just anticipating those fires.* PIP_9

PIPs explained that such lack of understanding about the medicine supply process led to stopped medicines still being dispensed and administered and this could be a patient safety issue. The majority reported developing enhanced relationships with community pharmacists and care home staff. If the PIP worked in the GP practice, these were often then sustained post-intervention, *'any query we just email the PIP we get information get straight from the horse's mouth'* CH Manager_14a.

A couple of PIPs explained that taking part in the intervention had prompted them to review systems in the GP surgery:

> *We have 5 people- pharmacy technician, pharmacy assistants, generating prescriptions, dealing with questions, and discharge letters, so one of them is now appointed for the nursing home patients. If we need to discuss anything, she's the one that helps if we make a change, she'll change it on their file, and then she'll liaise as well with the nursing home.* PIP_22

Rationalising record and stock

Working alongside care home staff PIPs allocated time to review MAR chart and consider if medication could move to PRN (as required) or homely medicines. The staff reported they appreciated dedicated time to discuss things and simplified MAR charts:

> *It sorted out things that we didn't need any longer and put things a bit more into place, like the PRNs particularly where some people didn't need it on their MAR charts any longer, … I*

For peer review only

> *suppose that had been overlooked which does happen I'm afraid, yes it was useful.* CH staff_19

Removing no-longer needed prescriptions and identifying changes in the ordering systems helped reduce workload, *'it really reduced our workload, in terms of kind of managing medication in the nursing home'* CH manager_11.

Many of the PIPs explained by regularly attending the care homes they were able to identify waste and unused stock and could take measures to reduce waste:

> *I spoke a few times with the dispensing pharmacists about errors that came up on the MAR chart etc. we tried to address those issues. I spoke to the appliance contractors not just the Pharmacy, … I had to address it in a really decisive manner.* PIP_16

The GP highlighting the importance of cost-saving inherent in stock control *'I wouldn't have had a clue about it* {over stock} *because I don't go into the store cupboards so that was a massive cost saving*' GP_16.

In summary, many PIPs were found to take an active lead in reviewing and advising on more efficient medication systems. However, their efforts to improve systems could be thwarted if either the care home staff or GP team were unwilling, or unable, to adopt new ways of working.

Discussion

This secondary analysis of data from the process evaluation of the CHIPPS study specifically focused on how the PIP role was enacted and PIPs, GPs and care home staffs' perceptions of the impact of PIP activity on care home medication procedures and resident wellbeing. The results provide contextualised understandings of the independent prescribing pharmacist role within the wider primary care team, with most PIPs and GPs suggesting PIPs should have a continued role in management of care home medication and residents' medicines. Importantly most participants reported perceptible benefits from PIP activity whether through improved resident wellbeing or increased safety and streamlining of care home medication procedures. Benefits from the intervention appeared greater when there was professional trust between GP, care home staff and the PIP. Examining exceptions can help better understand what resources, training or other preparation might be needed to be put in place for stakeholders to benefit from extended pharmacist roles.

GPs and PIPs made clear an independent prescribing pharmacist could bring specialised clinical knowledge to the multidisciplinary team and they saw a continuing place for pharmacists in care homes. While most care home teams valued the role of the PIP a few were still uncertain on the range of their specific responsibilities. Pharmacists have an increasingly valued place within multidisciplinary primary care teams, and this is evident within policy statements (22,8). However, importantly the pharmacist role needs to cover more than reviewing medicines to maximise impact. As part of a multidisciplinary team, pharmacists can provide dedicated specialist input into medicine

For peer review only

review by drawing on their clinical pharmaceutical knowledge (23). An independent prescriber pharmacist may be able to relieve the GP of some prescribing activity and so help to back fill GP shortages as fewer European GPs are now recruited (24).

The review of individual resident's medicines undertaken as part of this intervention was reported by care home staff and PIPs as having readily demonstrated benefits for residents' subjective quality of life. While the evidence on objective improvements in resident outcomes i.e., admission to hospital, falls, or mortality are inconclusive (25,26). Holland et al., (27) suggested that measures relating to older people's quality of life may be a better measure. Our study adds to this by indicating that measures of impact may need to be related to resident's everyday experiences, such as better engagement with others, more alert at mealtimes, rather than clinical risk of side effects from multiple no longer need medicines. We found that there needed to be trust between the care home staff and the PIP in order for some of the pharmacists' recommendations to be enacted; care home staff needed to have confidence the pharmacist would respond quickly if the resident's behaviour became distressed and a challenge to manage.

As part of the intervention service specification PIPs spent time actively reviewing, and where appropriate, improving care home medication systems. For example, reviewing and improving dispensing systems, consolidating MAR charts and monitoring stock control. These were reported as having benefits for care home staff in that administration and reordering was easier, and potentially safer. Improved management of stock reduced wastage and therefore had financial benefits. These findings resonate with evidence supporting the place of pharmacists within care homes (28). Nonetheless future work might explore if this is optimum use of a pharmacist's skills or if this might be a technician level activity.

The exceptions to the positive results reported so far occurred when relational factors inhibited the scope of pharmacist contributions such as when the relationship between pharmacist and GP or care home could not be successfully established. If care homes did not fully understand the clinical scope of the prescribing pharmacist, this led to limited opportunities for PIPs to undertake medicine reviews. PIPs indicated that for their role to be maximised there needed to be a clear understanding from GPs and care home staff about the role of the pharmacist and the level of clinical responsibility they held. This suggests the need for strategies which will develop shared understanding of the potential of each role. Our results resonate with a survey by Kahn et al. (29) that aimed to understand factors impacting on interprofessional working in primary care; they found that the time the team had worked together, and opportunities for formal and informal communication were important on enabling the team to develop trust and collaborative working practices. In our intervention the few PIPs who had not previously worked in the trial GP surgery reported greater resistance to their role and made fewer substantive changes to resident's medicines and care home medication systems. It is important that all stakeholders understand the pharmacist role as to support collaborative working practices (30).

For peer review only

A limitation of this research is that insights and understandings are from only a very specific group of pharmacists in care homes. All these pharmacists were independent prescribers, and all had received additional training in medicine for older people. It cannot be assumed that more generalist pharmacist in a GP surgery would have the experience to make the specialised medicine management decisions this group did. There are recommendations to make care home pharmacist a designated speciality (31). However, the bespoke training developed for this intervention appeared appropriate for enhancing pharmacists' clinical skills and confidence (32). Pharmacists in our study had medicine review responsibilities only for between 9 and 24 residents, so allowing them dedicated time to come to understand those resident's individual needs.

There are practice implications for other roles within the wider healthcare team. For example, during interviews PIPs referred to their work with community pharmacists describing the community pharmacist position as key in dispensing medicines. Future work exploring medications management in care homes might include the community pharmacist or dispensing pharmacy so that the efficiency of medicine ordering, and dispensing can be further optimised. This might reduce the safety risk, reported in this study, which occurs when medicines which have been deprescribed remain on the MAR sheet.

Conclusion

Independent prescribing pharmacists can successfully take responsibility for medication management and medicine review for older people in care homes. When they develop professionally trustful relationships with GP colleagues and care home staff, they can independently make changes to medicines which benefit resident wellbeing. Their expertise in medication systems including stock control and ordering enabled them to streamline care home medication systems with the potential benefit of reducing waste and likelihood of administration errors. The changing landscape of global primary care provisions indicates that pharmacists will continue to have a key role in leading management of medicines but that how this happens may require monitoring to enable refinement of the delivery model.

Author contribution: DW, CB, FP conceived the overall research design and provided commentary on the progress of the data collection and analysis. LB and LD undertook qualitative data collection and analysis, all authors reviewed emerging results. LB led on producing the manuscript. All authors commented on versions of the manuscript, and all agree to the final version.

Acknowledgements Thank you to residents and their families who agreed to receive the intervention, the pharmacists, GP practices and care homes who delivered the intervention and took part in the process evaluation. We would also like to acknowledge the South Norfolk Clinical Commissioning Group as the study sponsor and the CHIPPS Study team

For peer review only

Availability of data and materials: The datasets used and/or analysed during the current study are available from the corresponding author on reasonable request.

Competing interests DW received speaker fees from Desitin Pharma and speaker fees and unrestricted education grants from Rosemont Pharmaceuticals. All other authors have no competing/conflict of interest.

Ethical Approval English ethical approval was gained from East of England Cambridge Central Research Ethics Committee 17/EE/0360 (28.11.2017; this applied to research in Northern Ireland. Scottish ethical approval was gained from Scotland A research Ethics Committee 17/SS/0118 (07.12.2017).

Funding This is a summary of independent research funded by the National Institute for Health Research (NIHR) under its Programme Grants for Applied Research Programme (Grant Reference Number RP-PG-0613-20007). The views expressed are those of the authors and not necessarily those of the National Health Service, the NIHR or the Department of Health.

For peer review only

References

1. MacRae C, Henderson DAG, Mercer SW, et al. Excessive polypharmacy and potentially inappropriate prescribing in 147 care homes: a cross-sectional study. *BJGP Open* 2021; 5(6): 0167. https://doi.org/10.3399/BJGPO.2021.0167

2. Pazan F, & Wehling M. Polypharmacy in older adults: a narrative review of definitions, epidemiology and consequences. *European Geriatric Medicine* 2021;12**,** 4 43–452. (https://doi.org/10.1007/s41999-021-00479-3

3. WHO. *Medication safety in polypharmacy: technical report*. 2019. Available: https://www.who.int/publications/i/item/WHO-UHC-SDS-2019.11

4. Davies LE, Spiers G, Kingston A, et al. Adverse Outcomes of Polypharmacy in Older People: Systematic Review of Reviews. *Journal of the American Medical Directors Association 2020;* 21(2):181-187. https://doi.org/10.1080/21548331.2019.1706995

5. Szczepura A, Wild D, & Nelson S. Medication administration errors for older people in long-term residential care. *BMC Geriatrics* 2011; 11(82). https://doi.org/10.1186/1471-2318-11-82

6. National Care Forum. *Preventing medication errors in care homes a review of publications.* 2019. Available:https://www.nationalcareforum.org.uk/wp-content/uploads/2019/11/Preventing-Medication-Errors.pdf

7. Care Quality Commission. *Regulation 12: safe care and treatment.* 2021. Available:https://www.cqc.org.uk/guidance-providers/regulations-enforcement/regulation-12-safe-care-treatment

8. Department of Health and Social Care. *Good for you, good for us, good for everybody A plan to reduce overprescribing to make patient care better and safer, support the NHS, and reduce carbon*

emissions. 2021. Available:https://assets.publishing.service.gov.uk/government/uploads/system/uploads/attachment_data/file/1019475/good-for-you-good-for-us-good-for-everybody.pdf

9. NHS England. *NHS England Funding and Resource 2018/19: Supporting 'Next Steps for the NHS Five Year Forward View'*. 2018. Available: NHS England » NHS England Funding and Resource 2018/19: Supporting 'Next Steps for the NHS Five Year Forward View'

10. General Pharmaceutical Council. *Pharmacist Independent Prescriber*. 2022. Available: https://www.pharmacyregulation.org/education/pharmacist-independent-prescriber

11. Bleidt BA. Deprescribing: an emerging role for pharmacists. *Journal of Pharmaceutical Health Services Research* 2019; 10(2):159–160. https://doi.org/10.1111/jphs.12303

12. Bond CM, Holland R, Alldred DP, et al. Protocol for a cluster randomised controlled trial to determine the effectiveness and cost-effectiveness of independent pharmacist prescribing in care homes: the CHIPPS study. *Trials* 2020, 21(1):103. https://doi.org/10.1186/s13063-019-3827-0

13. Bond CM, Holland R, Alldred DP, et al. Protocol for the process evaluation of a cluster randomised controlled trial to determine the effectiveness and cost-effectiveness of independent pharmacist prescribing in care home: the CHIPPS study. *Trials* 2020; 21(1):439. https://doi.org/10.1186/s13063-020-04264-8

14. Lane K, Bond C, Wright D, et al. "Everyone needs to understand each other's systems": Stakeholder views on the acceptability and viability of a Pharmacist Independent Prescriber role in care homes for older people in the UK. *Health Social Care Community* 2020; 28(5):1479–87.https://doi.org/10.1111/hsc.12970

15. Wright D J, Blyth A, Maskrey V, et al. Development and feasibility testing of an evidence-based training programme for pharmacist independent prescribers responsible for the medicines-related activities within care homes. *International Journal of Pharmacy Practice* 2021; 29(4):376-84. https://doi.org/10.1093/ijpp/riab025

16. Kelly LM, & Cordeiro M. Three principles of pragmatism for research on organizational processes. *Methodological Innovations* 2020; 13(2). https://doi.org/10.1177/2059799120937242

17. Inch J, Notman F, Bond CM, et al. The Care Home Independent Prescribing Pharmacist Study (CHIPPS)—a non-randomised feasibility study of independent pharmacist prescribing in care homes. *Pilot Feasibility Study* 2019*;* 5:89 . https://doi.org/10.1186/s40814-019-0465-

18. Moore GF, Audrey S, Barker M,. et al. Process evaluation of complex interventions: Medical Research Council Guidance. *BMJ* 2015; 350:h1258. doi:10.1136/bmj.h1258

19. Nowell LS, Norris JM, White DE, et al. Thematic Analysis: Striving to Meet the Trustworthiness Criteria. *International Journal of Qualitative Methods* 2017; 16(1). https://doi.org/10.1177%2F1609406917733847

20. Saldana J. *The coding manual for qualitative researchers*. 2021. London, Sage Publications Ltd.

21. Kieger ME, & Varpio L. Thematic analysis of qualitative data: AMEE Guide No. 131. *Medical Teacher* 2020; 42(8):846-854. https://doi.org/10.1080/0142159X.2020.1755030

22. International Pharmaceutical Federation (FIP). *FIP Regional Conference for the European Region*. 2020. The Hague: International Pharmaceutical Federation. Available: https://www.fip.org/file/4840

23. Baruth JM, Gentry MT, Rummans TA, et al. Polypharmacy in older adults: the role of the multidisciplinary team. *Hospital Practice* 2020; 48(sup1):56-62. https://doi.org/10.1080/21548331.2019.1706995

24. OECD. *Health at a Glance: Europe 2020 : State of Health in the EU Cycle Availability of doctors.* 2020. Available:https://www.oecd-ilibrary.org/sites/1d767767-en/index.html?itemId=/content/component/1d767767-en

25. Lee, SWH, Mak, VSL, Tang, YW. Pharmacist services in nursing homes: A systematic review and meta-analysis. Br J Clin Pharmacol. 2019; 85: 2668– 2688. https://doi.org/10.1111/bcp.14101

26. O'Mahony D. Optimization of Medication by Pharmacists in Older People With Multimorbidity for Improved Outcomes—Mirage or Reality? *JAMA Network Open* 2021; 4(4):e216392. doi:10.1001/jamanetworkopen.2021.6392

27. Holland R, Desborough J, Goodyer L, et al. (2008), Does pharmacist-led medication review help to reduce hospital admissions and deaths in older people? A systematic review and meta-analysis. British Journal of Clinical Pharmacology 2008; 65:303-316. https://doi.org/10.1111/j.1365-2125.2007.03071.x

28. Riordan DO, Walsh KA, Galvin R, et al. The effect of pharmacist-led interventions in optimising prescribing in older adults in primary care: A systematic review. *SAGE Open Medicine* 2016; *4*: 2050312116652568. https://doi.org/10.1177/2050312116652568

29. Khan AF, Barnsley J, Harris JK, et al. Examining the extent and factors associated with interprofessional teamwork in primary care settings. *Journal of Interprofessional Care* 2021; 36(1):52-63. https://doi.org/10.1080/13561820.2021.1874896

30. Zogas A, Gillespie C, Kleinberg F, et al. (2021). Clinical pharmacist integration into veterans' primary care: Team members perspectives. *The Journal of the American Board of Family Medicine* 2021; 34(2):320-327. https://doi.org/10.3122/jabfm.2021.02.200328

31.Burns C. *The Pharmaceutical Journal*, Recognise care home pharmacy as a specialism, says RPS Scotland;Online: DOI:10.1211/PJ.2019.20206469

32. Birt L, Dalgarno L, Bond CM, et al. Evaluation of a training programme for Pharmacist Independent Prescribers in a care home medicine management intervention. BMC Medical Education 2022; 22, 55. https://doi.org/10.1186/s12909-022-03575-5

For peer review only

Additional file 1 CHIPPS Intervention Service Specification

	Specification
**Recruitment and employment of the Pharmacist Independent Prescriber (PIP)**	
	Excellent interpersonal, communication and IT skills
	Familiarity with relevant GP software systems
	Experience of providing prescribing and medicines management advice and support
	Previous experience of working in GP practice environment
	Be able to travel to site locations
	A mobile phone to be contactable for the purposes of delivering this service
	Appropriate indemnity insurance for prescribing
**PIP roles and responsibilities**	
Review each resident's medication and develop and implement a pharmaceutical care plan (essential)	Optimise prescribing ensuring clear indication and evidence base for each medication (taking into consideration national and local pathways, guidelines and formularies), informed by tools such as STOPP/START
	Minimise the potential for adverse effects
	Optimise the dose of all medication
	Co-ordinate appropriate monitoring and associated tests for all medicines and conditions

Service specification 05.08.2020

For peer review only - http://bmjopen.bmj.com/site/about/guidelines.xhtml

	Agree initial care plan with GP, care staff and resident (where appropriate)
	Document and maintain records relating to review and care plan in GP and care home records as appropriate
Prescribing (essential)	
	Authorise repeat prescriptions
	Co-ordinate appropriate monitoring and associated tests for all medicines and conditions
	Deprescribe medicines according to agreed pharmaceutical care plan
	Document medication changes in GP and care home records and notify supplying pharmacy of all changes to medication within 24 hours
	Only initiate new medicines for existing diagnoses or for common ailments which can be managed with medicines classified by the Medicines and Healthcare products Regulatory Agency (MHRA) as Pharmacy (P) or General Sales List (GSL)
	Any additional areas of prescribing must be agreed and documented with the GP practice prior to prescribing (e.g. antibiotics for simple UTIs
Communication (essential)	
	Agree local protocols for communication with GP practice and care home prior to commencing service. This should include:<br>○ Process of communication and messaging

For peer review only - http://bmjopen.bmj.com/site/about/guidelines.xhtml

	○ The location and expected level of detail of all PIP interventions in the medical records Process and communication of referrals for activities outside the competence of the PIP
	Inform supplying community pharmacy about service and role (prior start of service)<br>○ Communicate all changes in medication to supplying pharmacy
	Complete all documentation and recording of activities as required by the study team.
Support systematic ordering, prescribing, and administration processes with each care home, GP practice and supplying pharmacy where needed: (undertaken at PIP's discretion)	
	Provide instructions on how to administer each drug
	Synchronise residents prescription quantities for monthly cycles
	Add or clarify directions for all medication where it is currently not clear
	Provide advice on repeat prescription ordering processes to:<br>○ Minimising missed items<br>○ Optimising quantities
	Optimise the use of homely remedies within the care home
	Reconcile resident medication following a transfer of care
Training provision (undertaken at PIP's discretion)	
	Review training needs of care home and GP practice and draft proposed training plan

Service specification 05.08.2020

For peer review only

	Provide training to care home staff on training needs basis from agreed list of potential topics/areas
	Provide guidance to relevant GP practice on training needs basis from agreed list of potential topics/areas
**Safe and effective service provision**	
	PIP will be contactable and respond to messages within 24 hours (Monday - Friday)
	The PIP will establish a locally agreed protocol with the GP practice for referral/notification of all medicine related queries from CHIPPS participants to the PIP as appropriate (see 4.3.5)
	PIP will have full (read/write) access to GP record system to issues prescriptions and update records
	Where possible PIP will use remote access to update records when changes are made to GP held record<br>○ Where remote access is not feasible the PIP must update records within 24 hours of making a change
	PIP will have full (read/write) access to care home records to update records during all visits using appropriate local reporting systems
	The PIP will visit/contact the care home at least once a week
	The PIP will visit/contact the GP practice at least once a week
	Wherever possible, all annual leave should be agreed before the beginning of the study. A clear system for transfer of responsibility communicated to GP, care home and supplying pharmacy

For peer review only - http://bmjopen.bmj.com/site/about/guidelines.xhtml

	The PIP will work within the local prescribing formularies of GP practice and primary care organisation.
	The PIP will report and document all significant clinical events or near misses using local reporting procedures and study documentation.
	Ensure all records are aligned

For peer review only

For peer review only - http://bmjopen.bmj.com/site/about/guidelines.xhtml

Consolidated criteria for reporting qualitative studies (COREQ): 32-item checklist

Developed from:
Tong A, Sainsbury P, Craig J. Consolidated criteria for reporting qualitative research (COREQ): a 32-item checklist for interviews and focus groups. *International Journal for Quality in Health Care*. 2007. Volume 19, Number 6: pp. 349 – 357

For study A qualitative understanding of outcomes when pharmacist independent prescribers lead on medicine management in older people's care homes.

No. Item	Guide questions/description	Reported on Page #
**Domain 1: Research team and reflexivity**		
Personal Characteristics		
1. Inter viewer/facilitator	Which author/s conducted the interview or focus group?	LB and LD
2. Credentials	What were the researcher's credentials? E.g. PhD, MD	LB has PhD with extensive experience in qualitative methods<br>LD has MA and experience of qualitative data collection and analysis
3. Occupation	What was their occupation at the time of the study?	Researcher
4. Gender	Was the researcher male or female?	Female
5. Experience and training	What experience or training did the researcher have?	LB PhD and 10 years post-doctoral experience<br>LD 5 years post masters experience
Relationship with participants		
6. Relationship established	Was a relationship established prior to study commencement?	.LB had not previous contact with participants<br>LD had had some contact with participants through data collection for RCT
7. Participant knowledge of the interviewer	What did the participants know about the researcher? e.g. personal goals, reasons for doing the research	Participants were aware the study was a follow on from the intervention study and the particular focus was on proactive deprescribing. This was in the participant information sheet

For peer review only

8. Interviewer characteristics	What characteristics were reported about the inter viewer/facilitator? e.g. Bias, assumptions, reasons and interests in the research topic	Reflexive iterative analysis was undertaken the researchers do not work in primary care and have no vested interest in the outcomes page 6
**Domain 2: study design**		
Theoretical framework		
9. Methodological orientation and Theory	What methodological orientation was stated to underpin the study? e.g. grounded theory, discourse analysis, ethnography, phenomenology, content analysis	The reported study uses thematic analysis within a pragmatic design Page 5/6
Participant selection		
10. Sampling	How were participants selected? e.g. purposive, convenience, consecutive, snowball	Purposive sampling of a defined bounded cohort all invited to interview and sampling framework in place Page 5
11. Method of approach	How were participants approached? e.g. face-to-face, telephone, mail, email	Contacted by email Page 5
12. Sample size	How many participants were in the study?	38
13. Non-participation	How many people refused to participate or dropped out? Reasons?	38 recruited from potential sample of 86 although several care home mangers had left the establishment. All you responded to recruitment email were interviewed. Page 6
Setting		
14. Setting of data collection	Where was the data collected? e.g. home, clinic, workplace	Data collected in person and by telephone page 5
15. Presence of non-participants	Was anyone else present besides the participants and researchers?	No
16. Description of sample	What are the important characteristics of the sample? e.g. demographic data, date	Table 1 reports sample characteristics
Data collection		
17. Interview guide	Were questions, prompts, guides provided by the authors? Was it pilot tested?	Not pilot tested but reviewed by experts and PPI and drew on earlier results from feasibility study page 4
18. Repeat interviews	Were repeat inter views carried out? If yes, how many?	NO
19. Audio/visual recording	Did the research use audio or visual recording to collect the data?	Audio recording Page6

For peer review only

20. Field notes	Were field notes made during and/or after the interview or focus group?	Researcher reflexive memos kept Page 6
21. Duration	What was the duration of the inter views or focus group?	30-90 minutes Page 6
22. Data saturation	Was data saturation discussed?	Not explicitly but strengths of sample discussed page 6
23. Transcripts returned	Were transcripts returned to participants for comment and/or correction?	No page 6
**Domain 3: analysis and findings**		
Data analysis		
24. Number of data coders	How many data coders coded the data?	Primarily LB and LD but codes discussed and developed with wider team Page6
25. Description of the coding tree	Did authors provide a description of the coding tree?	No
26. Derivation of themes	Were themes identified in advance or derived from the data?	Inductive analysis Page 6
27. Software	What software, if applicable, was used to manage the data?	NVivo
28. Participant checking	Did participants provide feedback on the findings?	The results were shared with a diverse research team for feedback Page 6
Reporting		
29. Quotations presented	Were participant quotations presented to illustrate the themes/findings? Was each quotation identified? e.g. participant number	Yes in results 9-14
30. Data and findings consistent	Was there consistency between the data presented and the findings?	Yes
31. Clarity of major themes	Were major themes clearly presented in the findings?	Yes
32. Clarity of minor themes	Is there a description of diverse cases or discussion of minor themes?	Yes discussion of minority of responses which indicated concern role of PIP interweaved in results and discussion

For peer review only

BMJ Open

What happens when pharmacist independent prescribers lead on medicine management in older people's care homes: a qualitative study?

Journal:	*BMJ Open*
Manuscript ID	bmjopen-2022-068678.R1
Article Type:	Original research
Date Submitted by the Author:	29-Dec-2022
Complete List of Authors:	Birt, Linda; University of East Anglia; University of Leicester<br>Dalgarno, Lindsay; University of Glasgow<br>Poland, Fiona; University of East Anglia, School of Health Sciences<br>Wright, David; University of East Anglia; University of Leicester<br>Bond, Christine; University of Aberdeen
**Primary Subject Heading**:	Geriatric medicine
Secondary Subject Heading:	Health services research
Keywords:	PRIMARY CARE, CLINICAL PHARMACOLOGY, GERIATRIC MEDICINE, Risk management < HEALTH SERVICES ADMINISTRATION & MANAGEMENT, QUALITATIVE RESEARCH

SCHOLARONE™
Manuscripts

I, the Submitting Author has the right to grant and does grant on behalf of all authors of the Work (as defined in the below author licence), an exclusive licence and/or a non-exclusive licence for contributions from authors who are: i) UK Crown employees; ii) where BMJ has agreed a CC-BY licence shall apply, and/or iii) in accordance with the terms applicable for US Federal Government officers or employees acting as part of their official duties; on a worldwide, perpetual, irrevocable, royalty-free basis to BMJ Publishing Group Ltd ("BMJ") its licensees and where the relevant Journal is co-owned by BMJ to the co-owners of the Journal, to publish the Work in this journal and any other BMJ products and to exploit all rights, as set out in our licence.

The Submitting Author accepts and understands that any supply made under these terms is made by BMJ to the Submitting Author unless you are acting as an employee on behalf of your employer or a postgraduate student of an affiliated institution which is paying any applicable article publishing charge ("APC") for Open Access articles. Where the Submitting Author wishes to make the Work available on an Open Access basis (and intends to pay the relevant APC), the terms of reuse of such Open Access shall be governed by a Creative Commons licence – details of these licences and which Creative Commons licence will apply to this Work are set out in our licence referred to above.

Other than as permitted in any relevant BMJ Author's Self Archiving Policies, I confirm this Work has not been accepted for publication elsewhere, is not being considered for publication elsewhere and does not duplicate material already published. I confirm all authors consent to publication of this Work and authorise the granting of this licence.

For peer review only

What happens when pharmacist independent prescribers lead on medicine management in older people's care homes: a qualitative study?

Linda Birt[1,2], Lindsay Dalgarno,[3,4] Fiona Poland,[1] David Wright,[5,2] Christine Bond[3]

1.School of Health Sciences, University of East Anglia

2.School of Healthcare, University of Leicester

3.Institute of Applied Health Sciences, University of Aberdeen, Aberdeen, UK School of HealthCare, University of Leicester

4.General Practice and Primary Care, University of Glasgow

5. School of Pharmacy, University of East Anglia

Corresponding author

Dr Linda Birt

School of Health Sciences

Queen's Building

University of East Anglia

Norwich Research park

Norwich

NR4 7TJ

Linda.birt@uea.ac.uk

For peer review only

For peer review only - http://bmjopen.bmj.com/site/about/guidelines.xhtml

What happens when pharmacist independent prescribers lead on medicine management in older people's care homes: a qualitative study?

Abstract

Objective: Older people in care homes frequently experience polypharmacy, increasing the likelihood of medicine-related burden. Pharmacists working within multidisciplinary primary care teams are ideally placed to lead on medication reviews. A randomised controlled trial placed pharmacists, with independent prescribing rights (~~PIPs~~), into older people care homes. In the intervention service PIPs worked with general practitioners (GPs) and care home staff for 6 months, to optimise medicine management at individual resident and care home level. PIPs activity included stopping medicines that were no longer needed or where potential harms outweighed benefits. This analysis of qualitative data examines health and social care stakeholders' perceptions of how the service impacted on care home medicine procedures and resident wellbeing.

Design: Pragmatic research design with secondary analysis of interviews

Setting: Primary care pharmacist intervention in older people care homes in England, Scotland and Northern Ireland

Participants: Recruited from intervention arm of the trial: Independent prescribing pharmacists (n=14), General Practitioners (n=8) Care home managers (n=9) Care home Staff (n=6)

Results: There were resonances between different participant groups about potential benefits to care home residents of a medicine service provided by PIPs. There were small differences in perceptions about changes related to communication between professionals. Results are reported through three themes 1) '*It's a natural fit'* – pharmacists undertaking medication review in care homes fitted within multidisciplinary care; 2) *'The resident is cared for'* - there were subjective improvements in residents' wellbeing; 3) *Moving from 'firefighting' to effective systems* - there was evidence of changes to care home medicine procedures.

Conclusion: This study suggests that pharmacist independent prescribers in primary care working within the multi-disciplinary team can safely manage care home residents' medicines leading to subjective improvements in residents' well-being and medicine management procedures. Care home staff appreciated contact with a dedicated person in the GP practice.

Strengths and limitations of this study

- The study draws on data from a relevant sample of 37 health and social care professionals involved in medicine management in care homes

For peer review only

- The analytical approach foregrounded the effect of pharmacist-led medication review on the older resident in care homes, therefore having relevance to practices supporting safer medicines in this group of people.
- A limitation is the absence of residents' voices, due to recruitment challenges with this group
- The study provides insights into activity provided by specialist pharmacists with independent prescribing qualifications so it cannot be assumed that more generalist pharmacist would have the experience to make specialised medicine management decisions.

Key words Pharmacist prescriber, care home, older people, primary care, medication, general practice, multidisciplinary health care,

For peer review only

Introduction

Older people in care homes are often subject to complex medicine regimes which may include the concurrent administration of more than five different medicines daily (1,2). This is known as polypharmacy; it is associated with a higher risk of adverse side effects including falls, hospitalization and mortality (3,4). The complexity of managing medicines and administering multiple medicines increases care home staff workload (5). This can lead to increased rates of medicine administration errors, and while few errors result in serious consequences for residents, all need to be reported and followed-up (6). The Care Quality Commission highlights management of medicines as an area of concern and holds it under constant review (7). In United Kingdom care homes, prescribing and medication reviews for individual residents are the responsibility of clinical professionals in the primary care team (8). A Structured Medication Review (SMR) is recommended for all care home residents. (9) An SMR takes a personalised approach drawing on shared decision-making principles to increase safety and efficacy (9). Pharmacists are being routinely employed to lead on SMRs in primary care practices, and an English government initiative to enable pharmacists to have a role within care homes is gathering momentum (10). Pharmacists have specialised knowledge of medicine-related burden (11) including 'drug burden' where the cumulative effect of the active ingredients of medicines can cause physical and cognitive function (12). If pharmacists have the postgraduate Independent Prescriber qualification, they can start (prescribe) or stop (deprescribe) medicines within their area of clinical competence (13,14). This enhanced role allows them to use their expert clinical knowledge of medicines to deliver services in care homes.

This paper reports on a secondary analysis of interview data collected from pharmacists with independent prescribing rights (PIPs), general practitioners (GPs) and care home (CH) staff as part of the process evaluation (15) undertaken as part of the Care Home Independent Prescribing Pharmacist Study (CHIPPS). Within the trial a service intervention was designed which enabled pharmacist-led medicine management for care homes. PIPs worked within care homes on a weekly basis over 6 months to review their medicine systems and residents' personal medicines (16). PIPs were based within the general practice aligned to the care home. The secondary analysis aimed to understand how the PIP role was enacted and PIPs, GPs and CH staffs' perceptions of how PIP activity impacted on care home medicine procedures and resident wellbeing, concepts which were outside the remit of the trial process evaluation (17).

Methods

The CHIPPS study

The pharmacist-led service offered in care homes is briefly described below to set the findings of the secondary analysis in the practice context; full details of the trial design are available at (12) and trial outcomes are available (18).

The pharmacist-led service was delivered through a triad of GP, PIPs based at the GP practice, and up to 24 care home residents, from up to three care homes, registered with the GP. Service

For peer review only - http://bmjopen.bmj.com/site/about/guidelines.xhtml

specifications were developed following focus groups with stakeholders to guide the PIPs work, see supplementary file 1. The primary trial outcome was reduction in falls for residents (18). However, in a pre-study focus group health and social care professionals (HSCPs) wondered whether there might be wider benefits for residents (19). The communication between trial participants, their understanding of responsibilities of PIPs and outcomes for residents are the focus of this secondary analysis of process evaluation interview data. Within the trial 25 triads were recruited to the intervention arm. Sixteen PIPs were already working with the GP; the remaining nine were allocated a GP practice for the purposes of the study. PIPs undertook a two-day bespoke face-to-face training programme, produced a portfolio of evidence of competencies and finally were signed off to prescribe in older people medicine by an independent GP (20). To spread researcher workload (triad recruitment, data collection) the intervention was delivered over four six-month phases between January 2018 and March 2020. Data collection for the process evaluation occurred between May 2019 and March 2020 as each phase completed. The secondary analysis of interview data was carried out by (LB) and (LD).

Design

Pragmatism is a research methodology suited to characterising knowledge about how behaviours and actions impact on healthcare systems and outcomes. This approach can help appreciate the value of knowledge within its context of activity, uses and how these relate to the experience of addressing practical problems (21). As this work aimed to understand and learn from the diverse ways different stakeholders engaged with and perceived the intervention, a pragmatic design provided a way to recognise multiple realities of people involved. English ethical approval was gained from East of England Cambridge Central Research Ethics Committee 17/EE/0360 (28.11.2017; this applied to research in Northern Ireland. Scottish ethical approval was gained from Scotland A research Ethics Committee 17/SS/0118 (07.12.2017). Written informed consent was obtained from all subjects.

Recruitment and sample

During the process evaluation health and social care stakeholders in each triad where the intervention was delivered were invited to take part in a semi-structured interview at the end of each 6-month phase: PIP n=23 (2 withdrew and contact details not available) GP n=25, CH managers n=38). A reminder email was sent after two weeks. CH managers provided recruitment information to staff, residents and relatives. Those interested in participating returned expressions of interest to study researchers. A purposive sampling framework was designed to ensure representation across stakeholder roles, location and phases of the intervention. However, response rates were low and therefore all those replying were invited to interview.

Data collection

For peer review only - http://bmjopen.bmj.com/site/about/guidelines.xhtml

The process evaluation semi-structured interview guide drew on outcomes from the earlier feasibility study (22) and reflected the Medical Research Council process evaluation domains (23). Questions explored participants' experiences of the service intervention, anticipated and unanticipated outcomes including multidisciplinary communication and outcomes for residents (see supplementary file 2). Interviews were undertaken by (LB) and (LD) academic researchers, between May 2019 and March 2020, either in person or by telephone and audio recorded and professionally transcribed. Informed consent was obtained before interview.

Data management and analysis

An inductive thematic analysis was used to characterise participants' experiences of delivering or receiving the pharmacist-led service. Thematic analysis provides a structured, methodical way for the researcher to familiarise with the data, and to organise, analyse and report findings; importantly it is not strongly aligned to any epistemological stance (24) so was appropriate within the pragmatic design. Both researchers familiarised themselves with the data through checking transcripts for accuracy, then reading and making analytical memos (25). Next, they independently developed open codes (see supplementary file 3). The developing framework was discussed with the wider research team to refine the boundaries of the codes. Each interview was coded and any uncertainty arising during coding was discussed between researchers (see supplementary file 4). While the researchers predominantly coded interviews they had carried out, each coded two of the other's interviews to check for consistency of approach. This along with fortnightly review meetings increased dependability during analysis. The coding framework was revised iteratively as analyses progressed, for example grouping similar codes into a category of meaning. Categories were refined and, following discussions with the research team and patient and public partners, the final themes (overarching, abstracted semantic interpretations of the data (26) were developed. While a theme does not rely on specific quantities of data, here each theme provided extensive examples of data. To further support trustworthiness in results, illustrative examples of data within the theme were shared at research meetings to seek consensus from healthcare professionals, including pharmacists and general practitioners. Participant validation was not undertaken due to the time between interviews conducted in each phase and the end of study analysis.

For peer review only

Patient and Public Involvement

Public involvement was supported by the Patient and Public Involvement in Research group (further details at https://nspccro.nihr.ac.uk/working-with-us/public-patient-and-carer-voice-in-research). Public members were involved at all stages of the trial and in relation to this paper they reviewed data and provided comments on emerging interpretations. Public members had experience of having relatives receiving polypharmacy and of working in care homes; this enabled the research team to have confidence in their interpretations and to consider in greater detail potential resident focused benefits.

Results

For peer review only - http://bmjopen.bmj.com/site/about/guidelines.xhtml

Interviews, lasting between 30 and 90 minutes, were undertaken with 38 HCP: 14 PIPs, 9 GPs, 9 CH Managers and 6 CH staff. Participants were recruited from 18 of the 25 triads: six in Scotland, 4 in Northern Ireland and 8 in England. There was no expression of interest from any stakeholder in seven triads. In three of these triads the PIPs did not deliver the intervention; in the remaining 4 triads the demographics and trial outcomes were similar to the sample interviewed. Data was collected from a heterogenous sample of professionals and locations as shown in Table 1 (demographic characteristics of interview participants). Data were collected from triads with older people care homes that provide personal care and some social activities (residential) and dual registration care home which provide personal and nursing care. We identify illustrative quotes within quotation marks. For substantial quotes we provide professional role and unique triad number; non italicised text in quotes is for explanation.

For peer review only - http://bmjopen.bmj.com/site/about/guidelines.xhtml

For peer review only

Table 1 Demographic characteristics of interview participants

Intervention ID Interviewee N=38		General practice			Pharmacist			Care Home	
**Triad**	**Interviewee**	**Location**	**Patient list**	**Previously employed PIP**	**Time Independent prescriber**	**Previous experience in care homes**	**Number of residents in intervention**	**Type of registration**	**Ownership**
**1**	PIP, CH Manager	Northern Ireland	< 10,000	Yes	1 month	No	6	Dual	Private
**2**	PIP	England	≥10,000	Yes	2 months	Yes	20	Dual	Private
**3**	GP	England	< 10,000	No	12 months	Yes	16	Residential	Local Authority
**4**	PIP	Scotland	≥10,000	No	12 months	No	24	Dual	Private
**6**	PIP, GP, CH Manager CH Staff	England	≥10,000	NO	16 months	No	22	Residential	Voluntary
**8**	PIP, GP, CH Manager	England	≥10,000	Yes	18 months	Yes	24	Dual	Private
**9**	PIP CH Manager	England	≥10,000	Yes	18 months	No	21	Residential	Private
**11**	PIP, GP, CH Staff	Scotland	< 10,000	Yes	3 years	No	14	Residential	Private
**12**	CH manager	Northern Ireland	≥10,000	Yes	4 years	Yes	19	Residential	Voluntary
**14**	PIP, GP, CH Manager(x2) CH Staff	Scotland	< 10,000	Yes	4 years	No	20	Dual	Private
**15**	PIP	Scotland	< 10,000	Yes	6 years	Missing	18	Dual	Private
**16**	PIP, GP	England	< 10,000	No	6 years	Yes	20	Dual	Private
**17**	PIP	Northern Ireland	Missing	Yes	7 years	Yes	6	Dual	Private
**18**	CH manager	Scotland	< 10,000	Yes	8 years	No	9	Dual	Private

For peer review only - http://bmjopen.bmj.com/site/about/guidelines.xhtml

**19**	PIP, GP, CH Staff	England	< 10,000	No	9 years	Yes	24	Residential	Private
**20**	PIP	Northern Ireland	Missing	Yes	10 years	Yes	23	Dual	Private
**21**	GP, CH Manager(x2) CH Staff	Scotland	< 10,000	Yes	14 years	Yes	21	Dual	Private
**22**	PIP	Scotland	< 10,000	Yes	16 years	Yes	11	Dual	Private

For peer review only

Three themes were developed. Theme 1) *'It's a natural fit' -multidisciplinary working in care homes* explores GP, PIP and care home staff differing experiences of this new role; 2) *'The resident is cared for' -shared goals in medicine management* provides exemplars of improvement in residents experience of medication and the impact of targeted deprescribing; 3) *Moving from 'firefighting' to effective systems* reports on the improvement to organisation systems across care homes and GP practices and whether this lasted beyond the intervention. Participants mostly recounted positive experiences but some identified challenges inherent in pharmacist-led medicine management in care homes.

Theme 1' It's a natural fit'- multidisciplinary working in care homes

This theme reports GP, care home staff and PIPs experiences of pharmacist-led medicine management. The GP and care home staff valued the PIPs activity for differing reasons, suggesting a PIP role can meet both sets of needs.

GP perceptions

GPs highlighted the safety advantage of having PIPs within the multidisciplinary team explaining that it was helpful to have a 'another pair of eyes' to increase patient safety:

> *'Having a pharmacist who had good knowledge of all kinds of medications, going through polypharmacy, with a fine-tooth comb and picking up any errors or things we could do better'.* GP_21

GPs also drew on PIPs expertise of medicine administration for the wider benefit of care home residents, *'things that can be crushed or can be opened or whether they need to be changed to suspensions'*, explaining the PIP '*seems to know the answer for everything with regards to medication interactions and probably the thing I ask her most is appliances, different catheters and things*' GP_8.

GPs explained that as PIPs could spend several hours within the home with a small number of residents, they could understand residents' medicine needs in greater detail, '*it has brought up things that I might not have thought about'* GP_16. GPs and care home staff were generally open to suggestions from PIPs. However, two PIPs not based in the GP practice prior to the intervention struggled to have their prescribing role accepted, *'I couldn't just go off and prescribe things because the doctor would need to know … it was quite obvious she didn't want me to do anything without putting it through her'* PIP_19. This may suggest a need for actively reviewing and clarifying accountability of roles so that PIPs may be fully integrated into the multidisciplinary team.

While GPs found it hard to quantify the impact of the intervention on their workload, on reflection they mostly described how they were now dealing with fewer daily enquiries from care homes. Where PIPs and GP had established communication channels, the PIPs regularly identified residents and issues that the GP should look at in their weekly ward round:

For peer review only - http://bmjopen.bmj.com/site/about/guidelines.xhtml

> *'It has reduced the time taken to see patients as I can be confident their meds are all up to date, tests required for routine monitoring have been flagged up and I have been able to action these'.* GP_3

Not all GPs agreed that the PIP intervention was a 'natural fit', rather one GP described a situation where they believed the intervention had increased their workload as the PIP had requested biochemical monitoring which was '*creating work…actual fact I didn't change resident's dose, there was no indication to do so and I did wonder what I was trying to achieve.'* GP_19. In part this situation arose as the PIP did not have full access to the resident's clinical records and was unaware a blood test had been taken during a recent hospital visit, highlighting the importance of PIPs being able to have full access to the primary care team's systems and patient records.

Care home team perceptions

Care home managers and staff identified benefits in having a dedicated pharmacist attached to the home. Most care home teams explained they had gained improved accessibility and advice by having PIPs as point of contact in the primary care team, '*if you're phoning a surgery about a pharmacy issue, probably better speaking to a pharmacist than a GP'* CH manager_21a.

Where multidisciplinary working was embedded care home teams explained the clinical skills of each professional were respected, and teamwork supported effective resident care:

> *'The* [CH] *nurses' assessments are being taken as a valuable tool, because the nurses are observing, the nurses are giving the assessment, and the nurses are liaising with the PIP, to prescribe what they think is needed, so I think it's a win, win situation, the nurses are feeling valued, and the PIP as well'.* CH manager_21b

However, in two homes PIPs was not able to become integrated into the care home. In one the PIP reported that care home staff were unclear of their role. In another there was confusion about the legal position of PIPs to prescribe and the manager insisted the GP signed off all the PIP's prescribing activity. This highlights the importance of understanding and trust across the triad.

PIP perceptions

PIPs commonly reported that their prescribing authority meant decisions and residents care could be followed up in a timely way:

> *'I think the benefits of having pharmacists in Primary Care is we can go in and make the changes and own it and follow it up rather than pass it on, …leaving it for other people to follow up'.* PIP_17

A few PIPs mentioned the role of the pharmacy technician as potentially supporting their work, as pharmacy technicians would undertake stock control and check medicine administration records for accuracy with stock. Only one PIP appeared to have worked with a technician during the intervention '*…a [pharmacy] technician that's employed to go around the homes, I had a relationship*

For peer review only - http://bmjopen.bmj.com/site/about/guidelines.xhtml

with them already, … a lot of the work that they had already done, …so it meant me going in wasn't as huge job to turn around the whole care home' PIP_14. A GP pointed out that it may not be good use of a pharmacist '*to be going in and counting tablets and working out what the home needed'* GP_21.

In summary GPs and PIPs made clear what expertise an independent prescribing pharmacist could bring to the multidisciplinary team and saw a continuing place for pharmacists in care homes. While most care home teams valued the PIP role a few were still uncertain on the range of their specific responsibilities.

Theme 2 'The resident is cared for' - shared goals in medicine management

GPs and PIPs highlighted a strength of having a PIP as 'the linchpin in medicine management' in that they had detailed pharmaceutical knowledge allowing them to take actions which would directly and specifically benefit residents. PIPs reviewed prescriptions and could actively seek to rectify any prescribing errors and reduce the drug burden related to adverse effects from medicines. By doing this they importantly improved safety for the resident and helped to make MAR (Medication administration record) sheets fit for purpose thereby potentially reducing the risk of administration error. Changes PIPs could make ranged from small actions such as changing the time of eye-drop administration, so residents were not woken every night by staff, and changing tablet preparations to soluble form, to complex titrating of medicines, for example, reducing doses of morphine-based painkillers and stopping antipsychotic medicines. PIPs reviewed all medicines in the context of a resident's current biomedical markers and stage of life.
Residents, like others in the multidisciplinary team, were sometimes uncertain of the PIP's credentials, and PIPs reported that a few residents were reluctant to have their medicines reviewed preferring to remain with prescriptions given by the GP or secondary care team. There were a few triads where care home teams struggled to recall changes which had impacted on residents' wellbeing. These proved to be either homes where PIPs struggled to build working relationships and therefore their medication activity was negligible, or homes that already had highly developed relationships with either the PIP allocated to the intervention or with another pharmacist. In this latter case it was reported that most residents already received regular medication reviews.

Managing resident prescriptions

Independent prescribing activity was a key role of PIPs in the multidisciplinary team. Their involvement could directly benefit residents, often because this could progress a new prescription much more speedily:

> *'If my residents are feeling their symptoms, then refer to the PIP, and the PIP will prescribe immediately, there will immediate response, and then the residents will be happy'.*
> CH manager_21b

Other care home teams identified how having direct contact with a PIP stopped conditions

For peer review only - http://bmjopen.bmj.com/site/about/guidelines.xhtml

worsening, '*somebody having dry skin, and being able to contact a pharmacist, definitely improved care and stopped things becoming more acute, skin breaking down'* CH Manager_16. They explained that for such conditions they would not have contacted the GP but would have waited for the weekly GP visit, with the ensuing delay potentially leading to the worsening of conditions.

Key to successful deprescribing, reducing or stopping a medicine, was the PIP having trustful relationships with the care home team, the GP, the resident or their family. The importance of this trustful relationship was made evident when care home teams explained how they were now confident in trying to titrate prescriptions down as they knew the PIP was easy to contact and had agreed that if there were adverse effects the process could be stopped, and the medicine reinstated.

> *'Seeing PIP on a weekly basis meant we were able to make some huge reductions but also we were both very honest with each other so when we had got rid of almost everything and then resident's behaviour got worse again, we were able to add in a tiny little bit of something and I mean tiny bit which was appropriate'*. CH Manager_9

Titrating anti-psychotic medicine is a complex process and each stakeholder needs to be fully committed and in good communication with each other. Here a PIP explains how the process continued after the intervention ended, illustrating the continued benefit of having PIPs working in the GP practice:

> *'We had started to titrate it down and for a few days they were okay and then they started to need a lot of diazepam so we increased it back up but now it has come back down by half; they are checking and watching even though the study has ended because I have been involved and generally keeping an eye on things too they still have my contacts'. PIP_1*

Discussions on deprescribing were also enabled between PIPs and the resident or their relative, thereby providing more patient-centred care. A care home manager explains:

> *'…talking through that with the PIP and the resident she has come to a circle and is off her Butec patches with no pain; that was agreed with her and her family … she didn't mind coming off and giving it a trial period'*. CH Manager_6

Shared decisions

While it was intended that PIPs would be actively engaged with residents as part of the intervention, frequently residents did not have the mental capacity for this. However, even though a PIP stated that residents would possibly have little recall of their conversation they still found it relationally valuable to meet with residents '*I enjoyed meeting them all and then they become more than just a name on a page, you remember the person behind the medication'* PIP_1. A few PIPs also reported the benefit of engaging with family:

> *'I could really get on well with the family, she wanted to know everything what I was going to do so that was more interesting because I could discuss things, a really good example of joint working with the family'*. PIP_16

For peer review only - http://bmjopen.bmj.com/site/about/guidelines.xhtml

In summary most participants readily identified ways in which the intervention had directly benefited individual residents. The key factor enabling PIPs to work in ways which benefited residents stemmed from the trust the care home team, resident and their family could place on the PIP as a readily accessible, clinically competent person who could react appropriately if adverse consequences arose to any medicine change.

Theme 3 Moving from firefighting to effective systems

Within the care home, most of the PIPs actively reviewed and where appropriate improved medicine ordering and administration systems, for example streamlining dispensing systems, consolidating MAR charts and stock control.

Streamlining medicine supply and stock control

PIPs could articulate their detailed knowledge of the role of the community pharmacist and the processes which may be in place when a medicine is started or stopped, particularly the time it can take for automated systems to register the change. This 'insider knowledge' placed PIPs in an ideal position to educate other professionals and try to streamline medicine ordering processes:

> '*Geriatrician didn't realise that although it was stopped in the MAR chart the next month's is out there in the van waiting to be delivered, just anticipating those fires'.* PIP_9

PIPs explained that such lack of understanding about the medicine supply process led to stopped medicines still being dispensed and administered and this could be a patient safety issue. The majority reported developing enhanced relationships with community pharmacists and care home staff. If the PIP worked in the GP practice, these relationships were often sustained post-intervention, *'any query we just email the PIP we get information get straight from the horse's mouth'* CH Manager_14a.

A few PIPs explained that taking part in the intervention had prompted them to review systems in the GP surgery:

> *'We have 5 people- pharmacy technician, pharmacy assistants, generating prescriptions, dealing with questions, and discharge letters, so one of them is now appointed for the nursing home patients. If we need to discuss anything, she's the one that helps if we make a change, she'll change it on their file, and then she'll liaise as well with the nursing home'.* PIP_22

Rationalising record and stock

Working alongside care home staff PIPs allocated time to review MAR chart and consider if medicine could move to PRN (as required) or homely medicines. The staff reported they appreciated dedicated time to discuss things and simplified MAR charts:

> *'It sorted out things that we didn't need any longer and put things a bit more into place, like the PRNs particularly where some people didn't need it on their MAR charts any longer, … I*

For peer review only - http://bmjopen.bmj.com/site/about/guidelines.xhtml

suppose that had been overlooked which does happen I'm afraid, yes it was useful'. CH staff_19

Removing no-longer needed prescriptions and identifying changes in the ordering systems helped reduce workload, *'it really reduced our workload, in terms of kind of managing medication in the nursing home'* CH manager_11.

Many of the PIPs explained that by regularly attending the care homes they were able to identify waste and unused stock and could take measures to reduce waste:

'I spoke a few times with the dispensing pharmacists about errors that came up on the MAR chart etc. we tried to address those issues. I spoke to the appliance contractors not just the Pharmacy, … I had to address it in a really decisive manner'. PIP_16

One GP highlighted the importance of cost-saving inherent in stock control *'I wouldn't have had a clue about it* {over stock} *because I don't go into the store cupboards so that was a massive cost saving*' GP_16.

In summary, many PIPs were found to take an active lead in reviewing and advising on more efficient medicine systems. However, their efforts to improve systems could be thwarted if either the care home staff or GP team were unwilling, or unable, to adopt new ways of working.

Discussion

This secondary analysis of interview data from the process evaluation of the CHIPPS study (15) specifically focused on how the PIP role was enacted and PIPs, GPs and care home staffs' perceptions of the impact of PIP activity on care home medicine procedures and resident wellbeing. The results provide contextualised understandings of the independent prescribing pharmacist role within the wider primary care team, with most PIPs and GPs suggesting PIPs should have a continued role in management of care home medicine systems and residents' medicines. Importantly most participants reported perceptible benefits from the PIP intervention whether through improved resident wellbeing or increased safety and streamlining of care home procedures. Benefits from the intervention appeared greater when there was professional trust between GP, care home staff and the PIP. Examining exceptions can help better understand what resources, training or other preparation might need to be put in place for stakeholders to benefit from extended pharmacist roles.

GPs and PIPs made clear an independent prescribing pharmacist could bring specialised clinical knowledge to the multidisciplinary team and they saw a continuing place for pharmacists in care homes. While most care home teams valued the role of the PIP a few were still uncertain on the range of their specific responsibilities. Pharmacists have an increasingly valued place within multidisciplinary primary care teams, and this is evident within policy statements (27,8). However, importantly the pharmacist role needs to cover more than reviewing medicines to maximise impact. As part of a multidisciplinary team, pharmacists can provide dedicated specialist input into

For peer review only - http://bmjopen.bmj.com/site/about/guidelines.xhtml

Structured Medication Reviews (9) by drawing on their clinical pharmaceutical knowledge (28). An independent prescriber pharmacist may be able to relieve the GP of some prescribing activity and so help to back fill GP shortages as fewer European GPs are now recruited (29).

The review of individual resident's medicines undertaken as part of this intervention was reported by care home staff and PIPs as having readily demonstrated benefits for residents' subjective quality of life. This understanding is important as the evidence on objective improvements in resident outcomes i.e., admission to hospital, falls, or mortality from this trial and others is inconclusive (18,30,31). Holland et al., (32) suggested that measures relating to older people's quality of life may be a better measure. Our study supports this suggestion by indicating that measures of impact may need to be related to resident's everyday experiences, such as better engagement with others, more alert at mealtimes, rather than clinical risk of side effects from multiple no longer need medicines. We found that there needed to be trust between the care home staff and PIPs in order for some of the pharmacists' recommendations to be enacted; care home staff needed to have confidence the pharmacist would respond quickly if the resident's behaviour became distressed and a challenge to manage.

As part of the intervention service specification PIPs spent time actively reviewing, and where appropriate, improving care home medicine systems. For example, reviewing and improving dispensing systems, consolidating MAR charts and monitoring stock control. These were reported as having benefits for care home staff in that administration and reordering was easier, and potentially safer. Improved management of stock reduced wastage and therefore had financial benefits. These findings resonate with evidence supporting the place of pharmacists within care homes (33). Nonetheless future work might explore if this is optimum use of a pharmacist's skills or if this might be a technician level activity.

The exceptions to the positive results reported so far occurred when relational factors inhibited the scope of pharmacist contributions. For example, when the relationship between pharmacist and GP or care home could not be successfully established or understanding of the pharmacists legal clinical scope was not understood. This suggests the need for strategies which will develop shared understanding of the potential of each other's roles. Our results resonate with a survey by Kahn et al. (34) that aimed to understand factors impacting on interprofessional working in primary care; they found that the time the team had worked together, and opportunities for formal and informal communication were important on enabling the team to develop trust and collaborative working practices. In our intervention the few PIPs who had not previously worked in the trial GP surgery reported greater resistance to their role and made fewer substantive changes to resident's medicines and care home medicine ordering and administration systems. It is important that all stakeholders understand the pharmacist role so as to support collaborative working practices (35).

Missing from our study are the resident and their relatives view of medication review and the place of the pharmacist in this. We attempted to recruit the residents consented to the intervention arm of the trial through care home staff. It is unclear why this was unsuccessful with only 3 residents

For peer review only - http://bmjopen.bmj.com/site/about/guidelines.xhtml

expressing an interest, but in part it is likely to be due to the severe cognitive impairment many residents were living with, meaning staff may not have actively encourage residents or their families to take part in the process evaluation interview. Therefore, a limitation of this research is that insights and understandings are from only a very specific group of HSCP involved in support older residents care homes. All pharmacists were independent prescribers, and all had received additional training in medicine for older people. It cannot be assumed that more generalist pharmacist in a GP surgery would have the experience to make the specialised medicine management decisions this group did. There are recommendations to make care home pharmacist a designated speciality (36). However further training may be needed as inexperienced pharmacists may rely on template driven reviews (37). The bespoke training developed for this intervention appeared appropriate for enhancing pharmacists' clinical skills and confidence (38). Pharmacists in our study had medication review responsibilities only for between 9 and 24 residents, so allowing them dedicated time to come to understand those resident's individual needs. An economic evaluation would also indicate if this level of pharmacist input would be cost effective in practice. It may be that some tasks could be allocated to pharmacy technicians.

There are practice implications for other roles within the wider healthcare team. For example, during interviews PIPs referred to their work with community pharmacists describing the community pharmacist position as key in dispensing medicines. Future work exploring medicines management in care homes might include the community pharmacist or dispensing pharmacy so that the efficiency of medicine ordering, and dispensing can be further optimised. This might reduce the safety risk, reported in this study, which occurs when medicines which have been deprescribed remain on the MAR sheet.

The CHIPPS study was completed in 2020 just a primary care networks (PCN) were being introduced in England and at the start of the COVID-19 pandemic. Within PCNs pharmacists work across several GP practices rather than being within a single practice (39). Our finding that PIPs not integrated within the GP team faced the most challenges in developing trustful working relationships mirrors an evaluation of PCNs published in 2022 (40). This evaluation reported the need for change in cultures and practices to support additional clinical roles in a GP practice (40,41). Those working within a PCN rather than a single GP surgery reported feeling a lack of autonomy, belonging and contribution, reflecting what PIPs 'dropped' into GP practices in the trial experienced.

Conclusion

Independent prescribing pharmacists can successfully take responsibility for medicine management and Structure Medication Reviews for older people in care homes. When pharmacists develop professional trustful relationships with GP colleagues and care home staff, they can independently make changes to medicines which benefit resident wellbeing. Their expertise in medicine systems including stock control and ordering enabled them to streamline care home systems with the potential benefit of reducing waste and likelihood of administration errors. The changing landscape of global primary care provisions indicates that pharmacists will continue to have a key role in

For peer review only - http://bmjopen.bmj.com/site/about/guidelines.xhtml

leading management of medicines but that how this happens may require monitoring to enable refinement of the delivery model.

Author contribution: DW, CB, FP conceived the overall research design and provided commentary on the progress of the data collection and analysis. LB and LD undertook qualitative data collection and analysis, all authors reviewed emerging results. LB led on producing the manuscript. All authors commented on versions of the manuscript, and all agree to the final version.

Acknowledgements Thank you to residents and their families who agreed to receive the intervention, the pharmacists, GP practices and care homes who delivered the intervention and took part in the process evaluation. We would also like to acknowledge the South Norfolk Clinical Commissioning Group as the study sponsor and the CHIPPS Study team

Availability of data and materials: The datasets used and/or analysed during the current study are available from the corresponding author on reasonable request.

Competing interests DW received speaker fees from Desitin Pharma and speaker fees and unrestricted education grants from Rosemont Pharmaceuticals. All other authors have no competing/conflict of interest.

Funding This is a summary of independent research funded by the National Institute for Health Research (NIHR) under its Programme Grants for Applied Research Programme (Grant Reference Number RP-PG-0613-20007). The views expressed are those of the authors and not necessarily those of the National Health Service, the NIHR or the Department of Health.

References

1. MacRae C, Henderson DAG, Mercer SW, et al. Excessive polypharmacy and potentially inappropriate prescribing in 147 care homes: a cross-sectional study. *BJGP Open* 2021; 5(6): 0167. https://doi.org/10.3399/BJGPO.2021.0167
2. Pazan F, & Wehling M. Polypharmacy in older adults: a narrative review of definitions, epidemiology and consequences. *European Geriatric Medicine* 2021;12**,** 4 43–452. (https://doi.org/10.1007/s41999-021-00479-3
3. WHO. *Medication safety in polypharmacy: technical report*. 2019. Available: https://www.who.int/publications/i/item/WHO-UHC-SDS-2019.11
4. Davies LE, Spiers G, Kingston A, et al. Adverse Outcomes of Polypharmacy in Older People: Systematic Review of Reviews. *Journal of the American Medical Directors Association 2020;* 21(2):181-187. https://doi.org/10.1080/21548331.2019.1706995

For peer review only - http://bmjopen.bmj.com/site/about/guidelines.xhtml

5. Szczepura A, Wild D, & Nelson S. Medication administration errors for older people in long-term residential care. *BMC Geriatrics* 2011; 11(82). https://doi.org/10.1186/1471-2318-11-82

6. National Care Forum. *Preventing medication errors in care homes a review of publications.* 2019. Available:https://www.nationalcareforum.org.uk/wp-content/uploads/2019/11/Preventing-Medication-Errors.pdf

7. Care Quality Commission. *Regulation 12: safe care and treatment.* 2021. Available:https://www.cqc.org.uk/guidance-providers/regulations-enforcement/regulation-12-safe-care-treatment

8. Department of Health and Social Care. *Good for you, good for us, good for everybody A plan to reduce overprescribing to make patient care better and safer, support the NHS, and reduce carbon emissions.* 2021. Available:https://assets.publishing.service.gov.uk/government/uploads/system/uploads/attachment_data/file/1019475/good-for-you-good-for-us-good-for-everybody.pdf

9. NHS England. *Structured Medication reviews and Medicine Optimisation*. No date Available https://www.england.nhs.uk/primary-care/pharmacy/smr/

10. NHS England. *NHS England Funding and Resource 2018/19: Supporting 'Next Steps for the NHS Five Year Forward View'*. 2018. Available: NHS England » NHS England Funding and Resource 2018/19: Supporting 'Next Steps for the NHS Five Year Forward View'

11. Mohammed MA, Moles RJ, Chen TF Medication-related burden and patients' lived experience with medicine: a systematic review and metasynthesis of qualitative studies *BMJ Open* 2016; 6:e010035. doi: 10.1136/bmjopen-2015-010035

12. Kouladjian L, Gnjidic D, Chen TF, Mangoni AA, Hilmer SN. Drug Burden Index in older adults: theoretical and practical issues. *Clinical Interventions in Aging* 2014; 9:1503-1515. doi:10.2147/CIA.S66660

13. General Pharmaceutical Council. *Pharmacist Independent Prescriber*. 2022. Available: https://www.pharmacyregulation.org/education/pharmacist-independent-prescriber

14. Bleidt BA. Deprescribing: an emerging role for pharmacists. *Journal of Pharmaceutical Health Services Research* 2019; 10(2):159–160. https://doi.org/10.1111/jphs.12303

15. Birt L, Dalgarno L, Wright DJ, et al. Process evaluation for the Care Homes Independent Pharmacist Prescriber Study (CHIPPS). *BMC Health Serv Res*. 2021;21(1):1041. Published 2021 Oct 2. doi:10.1186/s12913-021-07062-3

16. Bond CM, Holland R, Alldred DP, et al. Protocol for a cluster randomised controlled trial to determine the effectiveness and cost-effectiveness of independent pharmacist prescribing in care homes: the CHIPPS study. *Trials* 2020, 21(1):103. https://doi.org/10.1186/s13063-019-3827-0

17. Bond CM, Holland R, Alldred DP, et al. Protocol for the process evaluation of a cluster randomised controlled trial to determine the effectiveness and cost-effectiveness of independent pharmacist prescribing in care home: the CHIPPS study. *Trials* 2020; 21(1):439. https://doi.org/10.1186/s13063-020-04264-8

18. Holland R et al. The Care Homes Independent Prescribing Pharmacist Study (CHIPPS): A cluster randomised controlled trial to evaluate safety and effectiveness BMJ In press

For peer review only - http://bmjopen.bmj.com/site/about/guidelines.xhtml

19. Lane K, Bond C, Wright D, et al. “Everyone needs to understand each other’s systems”: Stakeholder views on the acceptability and viability of a Pharmacist Independent Prescriber role in care homes for older people in the UK. *Health Social Care Community* 2020; 28(5):1479–87.https://doi.org/10.1111/hsc.12970

20. Wright D J, Blyth A, Maskrey V, et al. Development and feasibility testing of an evidence-based training programme for pharmacist independent prescribers responsible for the medicines-related activities within care homes. *International Journal of Pharmacy Practice* 2021; 29(4):376-84. https://doi.org/10.1093/ijpp/riab025

21. Kelly LM, & Cordeiro M. Three principles of pragmatism for research on organizational processes. *Methodological Innovations* 2020; 13(2). https://doi.org/10.1177/2059799120937242

22. Inch J, Notman F, Bond CM, et al. The Care Home Independent Prescribing Pharmacist Study (CHIPPS)—a non-randomised feasibility study of independent pharmacist prescribing in care homes. *Pilot Feasibility Study* 2019*;* 5:89 . https://doi.org/10.1186/s40814-019-0465-

23. Moore GF, Audrey S, Barker M,. et al. Process evaluation of complex interventions: Medical Research Council Guidance. *BMJ* 2015; 350:h1258. doi:10.1136/bmj.h1258

24. Nowell LS, Norris JM, White DE, et al. Thematic Analysis: Striving to Meet the Trustworthiness Criteria. *International Journal of Qualitative Methods* 2017; 16(1). https://doi.org/10.1177%2F1609406917733847

25. Saldana J. *The coding manual for qualitative researchers*. 2021. London, Sage Publications Ltd.

26. Kieger ME, & Varpio L. Thematic analysis of qualitative data: AMEE Guide No. 131. *Medical Teacher* 2020; 42(8):846-854. https://doi.org/10.1080/0142159X.2020.1755030

27. International Pharmaceutical Federation (FIP). *FIP Regional Conference for the European Region*. 2020. The Hague: International Pharmaceutical Federation. Available: https://www.fip.org/file/4840

28. Baruth JM, Gentry MT, Rummans TA, et al. Polypharmacy in older adults: the role of the multidisciplinary team. *Hospital Practice* 2020; 48(sup1):56-62. https://doi.org/10.1080/21548331.2019.1706995

29. OECD. *Health at a Glance: Europe 2020 : State of Health in the EU Cycle Availability of doctors.* 2020. Available:https://www.oecd-ilibrary.org/sites/1d767767-en/index.html?itemId=/content/component/1d767767-en

30. Lee, SWH, Mak, VSL, Tang, YW. Pharmacist services in nursing homes: A systematic review and meta-analysis. Br J Clin Pharmacol. 2019; 85: 2668– 2688. https://doi.org/10.1111/bcp.14101

31. O’Mahony D. Optimization of Medication by Pharmacists in Older People With Multimorbidity for Improved Outcomes—Mirage or Reality? *JAMA Network Open* 2021; 4(4):e216392. doi:10.1001/jamanetworkopen.2021.6392

32. Holland R, Desborough J, Goodyer L, et al. (2008), Does pharmacist-led medication review help to reduce hospital admissions and deaths in older people? A systematic review and meta-analysis. British Journal of Clinical Pharmacology 2008; 65:303-316. https://doi.org/10.1111/j.1365-2125.2007.03071.x

For peer review only - http://bmjopen.bmj.com/site/about/guidelines.xhtml

33. Riordan DO, Walsh KA, Galvin R, et al. The effect of pharmacist-led interventions in optimising prescribing in older adults in primary care: A systematic review. *SAGE Open Medicine* 2016; *4*: 2050312116652568. https://doi.org/10.1177/2050312116652568

34. Khan AF, Barnsley J, Harris JK, et al. Examining the extent and factors associated with interprofessional teamwork in primary care settings. *Journal of Interprofessional Care* 2021; 36(1):52-63. https://doi.org/10.1080/13561820.2021.1874896

35. Zogas A, Gillespie C, Kleinberg F, et al. (2021). Clinical pharmacist integration into veterans' primary care: Team members perspectives. *The Journal of the American Board of Family Medicine* 2021; 34(2):320-327. https://doi.org/10.3122/jabfm.2021.02.200328

36. Burns C*. The Pharmaceutical Journal*, Recognise care home pharmacy as a specialism, says RPS Scotland;Online: DOI:10.1211/PJ.2019.20206469

37. Madden M, Mills T, Atkin K, Stewart S. Early implementation of the structured medication review in England: a qualitative study. *British Journal of General Practice* 2022; 72 (722): e641-e648. DOI: 10.3399/BJGP.2022.0014

38. Birt L, Dalgarno L, Bond CM, et al. Evaluation of a training programme for Pharmacist Independent Prescribers in a care home medicine management intervention. BMC Medical Education 2022; 22, 55. https://doi.org/10.1186/s12909-022-03575-5

39. Baird B., Beech J. *Primary Care networks explained*. The Kings Fund November 2020. Available https://www.kingsfund.org.uk/publications/primary-care-networks-explained

40. Baird b., Lamming L., Bhatt RT., Beech J., Dale V. Integrating additional roles into PCNs. The King's Fund February 2022. Available https://www.kingsfund.org.uk/sites/default/files/2022-02/Integrating%20additional%20roles%20in%20general%20practice%20report%28web%29.pdf

41. Mills T, Madden M, Stewart D, et al. Integration of a clinical pharmacist workforce into newly forming primary care networks: a qualitatively driven, complex systems analysis. *BMJ Open* 2022;12:e066025. doi:10.1136/bmjopen-2022-066025

For peer review only

For peer review only - http://bmjopen.bmj.com/site/about/guidelines.xhtml

Additional file 1 CHIPPS Intervention Service Specification

	Specification
**Recruitment and employment of the Pharmacist Independent Prescriber (PIP)**	
	Excellent interpersonal, communication and IT skills
	Familiarity with relevant GP software systems
	Experience of providing prescribing and medicines management advice and support
	Previous experience of working in GP practice environment
	Be able to travel to site locations
	A mobile phone to be contactable for the purposes of delivering this service
	Appropriate indemnity insurance for prescribing
**PIP roles and responsibilities**	
Review each resident’s medication and develop and implement a pharmaceutical care plan (essential)	Optimise prescribing ensuring clear indication and evidence base for each medication (taking into consideration national and local pathways, guidelines and formularies), informed by tools such as STOPP/START
	Minimise the potential for adverse effects
	Optimise the dose of all medication
	Co-ordinate appropriate monitoring and associated tests for all medicines and conditions

For peer review only - http://bmjopen.bmj.com/site/about/guidelines.xhtml

For peer review only

	Agree initial care plan with GP, care staff and resident (where appropriate)
	Document and maintain records relating to review and care plan in GP and care home records as appropriate
Prescribing (essential)	
	Authorise repeat prescriptions
	Co-ordinate appropriate monitoring and associated tests for all medicines and conditions
	Deprescribe medicines according to agreed pharmaceutical care plan
	Document medication changes in GP and care home records and notify supplying pharmacy of all changes to medication within 24 hours
	Only initiate new medicines for existing diagnoses or for common ailments which can be managed with medicines classified by the Medicines and Healthcare products Regulatory Agency (MHRA) as Pharmacy (P) or General Sales List (GSL)
	Any additional areas of prescribing must be agreed and documented with the GP practice prior to prescribing (e.g. antibiotics for simple UTIs
Communication (essential)	
	Agree local protocols for communication with GP practice and care home prior to commencing service. This should include:<br>○ Process of communication and messaging

For peer review only - http://bmjopen.bmj.com/site/about/guidelines.xhtml

	○ The location and expected level of detail of all PIP interventions in the medical records Process and communication of referrals for activities outside the competence of the PIP
	Inform supplying community pharmacy about service and role (prior start of service)<br>○ Communicate all changes in medication to supplying pharmacy
	Complete all documentation and recording of activities as required by the study team.
Support systematic ordering, prescribing, and administration processes with each care home, GP practice and supplying pharmacy where needed: (undertaken at PIP's discretion)	
	Provide instructions on how to administer each drug
	Synchronise residents prescription quantities for monthly cycles
	Add or clarify directions for all medication where it is currently not clear
	Provide advice on repeat prescription ordering processes to:<br>○ Minimising missed items<br>○ Optimising quantities
	Optimise the use of homely remedies within the care home
	Reconcile resident medication following a transfer of care
Training provision (undertaken at PIP's discretion)	
	Review training needs of care home and GP practice and draft proposed training plan

Service specification 05.08.2020

For peer review only - http://bmjopen.bmj.com/site/about/guidelines.xhtml

	Provide training to care home staff on training needs basis from agreed list of potential topics/areas
	Provide guidance to relevant GP practice on training needs basis from agreed list of potential topics/areas
**Safe and effective service provision**	
	PIP will be contactable and respond to messages within 24 hours (Monday - Friday)
	The PIP will establish a locally agreed protocol with the GP practice for referral/notification of all medicine related queries from CHIPPS participants to the PIP as appropriate (see 4.3.5)
	PIP will have full (read/write) access to GP record system to issues prescriptions and update records
	Where possible PIP will use remote access to update records when changes are made to GP held record<br>○ Where remote access is not feasible the PIP must update records within 24 hours of making a change
	PIP will have full (read/write) access to care home records to update records during all visits using appropriate local reporting systems
	The PIP will visit/contact the care home at least once a week
	The PIP will visit/contact the GP practice at least once a week
	Wherever possible, all annual leave should be agreed before the beginning of the study. A clear system for transfer of responsibility communicated to GP, care home and supplying pharmacy

For peer review only - http://bmjopen.bmj.com/site/about/guidelines.xhtml

	The PIP will work within the local prescribing formularies of GP practice and primary care organisation.
	The PIP will report and document all significant clinical events or near misses using local reporting procedures and study documentation.
	Ensure all records are aligned

For peer review only - http://bmjopen.bmj.com/site/about/guidelines.xhtml

CHIPPS WP6 QUAL Evaluation: Topic Guide for PIP

As the purpose of this interview is to encourage a conversation in which the participant can feel confident in expressing their own views, the following topic guide is indicative. This means that the interviewer may adapt it to suit the conversation style and preferences of the participant.

	**Introduction.**<br>**1. Introduce yourself**<br>**2. Explain the purpose of the research**	We have developed a new service in which a specially trained 'pharmacist independent prescriber' (PIP) becomes part of the care home team, working alongside general practitioners. The aim is to improve how resident's medicines are managed in a safe, effective and cost effective way. We hope this may improve resident's care, wellbeing and health outcomes. We are evaluating this new service in a large multi-centre randomised controlled trial in different places in the United Kingdom.<br>The study is run by the universities of East Anglia, Aberdeen, Leeds and Queen's Belfast. It is funded by the UK National Institute for Health Research.<br>*Since participating in the training for 'pharmacist independent prescribers (PIPs) ' you have set up and have delivered the Care Home Independent Pharmacists Prescriber service in the trial,*<br>*We would like you to consider your overall role as a PIP providing this service and your experience in delivering this. We would also like your views on the Service Specification and Pharmaceutical Care Plan, your thoughts about the mentoring you received and how useful the personal development plan and training was in preparing you for this role and how this has impacted on your ability to set up and deliver this service.*
	**The digital recorder**<br>**1. Stress confidentiality**	I would like to highlight the confidentiality of everything you tell me, and specifically that:<br>• *the recording will be deleted after being transcribed*<br>• *you won't be identified individually in any report.*<br>• *all information will be anonymised.*<br>• *we will not tell anyone else including your employer organisation, what you tell us as an individual.*<br>• *likewise no other individual e.g. GP, care home staff, resident will be identifiable in any report.*

For peer review only - http://bmjopen.bmj.com/site/about/guidelines.xhtml

CHIPPS Care Homes Independent Pharmacist Prescribing Study

	**2. Set ground rules**	*We do need to remind you, however, that* if you do disclose anything which might identify a risk to yourself or to others, or a personal or professional offence, this would be shared with the relevant responsible authority. However we would tell you if we thought this were the case.<br><br>• All of your views are of value to us. There are no right or wrong answers,<br>• Please ask me to clarify if the question isn't clear.<br>• We remind you not to share any personal or patient identifying information during this interview
	**Ask if there are any questions**	
	**Confirm consent**	

	**Stem question PIP service delivery**	**Probes / follow ups**
1.	What was your experience of delivering the service?	• Any particular issues<br>• Anything particularly good<br>(Interaction with GP/ care home staff/residents/ relatives access to records/ information/routines /types of interventions)<br>• Acceptability to residents without capacity
2	In what ways did your service affect resident care	• Examples positive and negative
3.	What are your views on the Service Specification?	• Clarity?<br>• Level of detail?<br>• Inclusion/exclusion criteria<br>• Service requirements?<br>• Outcomes<br>• Transitional arrangements
	Stem Question –research activity	•
34	What are your views on the research study documentation including: Pharmaceutical Care Plan, PIP log and resource use data?	• Ease of use<br>• Anything missing/ not needed<br>• Fit for purpose?

For peer review only

For peer review only - http://bmjopen.bmj.com/site/about/guidelines.xhtml

CHIPPS Care Homes Independent Pharmacist Prescribing Study

		• How much time did it take
		•
5.	What were your thoughts on the role of the mentor?	• Positive<br>• Negative<br>• Areas for improvements
6.	What was your experience of developing your own personal development plan with your mentor?	• Went well/ not so well<br>• Mentor support/ time scale for completion<br>• Accessing training on the identified development areas<br>• General acceptability
7.	What do you think about the competency framework assessment?	• Comprehensive?<br>• Easy/hard to demonstrate achievement?
78	What do you think about the assessor signing you off?	• Useful / difficult discussion?<br>• Appropriate?<br>• Alternative suggestions?<br>• Process fit for purpose?
	**Stem question Training**	**Probes / follow ups**
89	What are your thoughts on the impact of training you received?	Any gaps-<br>• *preparation for role,*<br>• *underpinning knowledge,*<br>• *competency framework,*<br>• *relationship building*<br>• *personal development skills*
10.	Now that you have been delivering the service for 3 months which particular elements of the training do you think have been most useful?	• Why?<br>• Relevance<br>• Time spent on this<br>• Delivery
11.	Which particular elements of the training do you think have been least useful in your role, delivering the service?	• Should we continue to provide this element of the training?

For peer review only - http://bmjopen.bmj.com/site/about/guidelines.xhtml

		• How should this element of training be delivered?<br>• What should we do to improve these elements of training?
11.	Is there anything that should have been included in the training that wasn't?	• How would you suggest that we train pharmacists in that?<br>• Is there existing training available, that you are aware of, to meet this<br>• Need?<br>• When should this training be provided?
12.	It is important to us that across the four areas, all PIPs provide a similar service. What would be the best way for this to be achieved?	• Training?<br>• Checklist?
13.	In what ways could you contribute to the training of the next cohort of Pharmacist Independent Prescribers?	• Mentor?<br>• Helping to deliver educational package locally?
14	How important for you was peer support	• use The Telegram group<br>• Use other ways if staying in contact
	**Stem question on team work**	•
8.	Where did communication issues arise between your and the other team members involved in the PIP service?	• Care homes<br>• Other Pharmacist<br>• Primary care team<br>• GP Practice<br>• Relatives/residents
9.	How do you think communication impacted on resident care? (positively/ negatively)	• Examples
14.	Any final comments?	
15.	Thank you for taking part in this interview	

N.B. all interview topic guides started with the setting the introduction and digital record prompts; in the remainder of the document we only include stem questions.

For peer review only - http://bmjopen.bmj.com/site/about/guidelines.xhtml

CHIPPS Care Homes Independent Pharmacist Prescribing Study

CHIPPS WP6 QUAL Evaluation: Topic Guide for GP

	**Stem question**	**Probes / follow ups**
Part 1	Intervention	
1.	Overall what are your views of the PIP service?	• New contributions<br>• New problems
	Views on changing skill mix in the primary care team<br>Views on changing skills mix in the care home team	• Diversity in team(s)<br>• Challenges<br>• Issues in managing
2.	How has the PIP service impacted on your work load?	• (Negatively/ positively)<br>• Time<br>• Visit frequency<br>• Referrals<br>• Tests<br>• Medication changes<br>• Medication reviews<br>• Medication ordering<br>• Repeat prescriptions
3.	In what ways has the PIP service impacted on patient care?	• Examples<br>• Acceptability for patients without capacity?
Part 2	Implementation	
4.	What barriers if any to implementing this service?	
	How could we overcome any barriers?	
5.	What did you see as facilitating the service to be implemented?	

For peer review only - http://bmjopen.bmj.com/site/about/guidelines.xhtml

For peer review only

	Stem question	Probes / follow ups
	**Stem question**	**Probes / follow ups**
Part 3	Working relationship	
6.	What is your view of your working relationship with the PIP?	• Good relationship areas<br>• Difficult relationship areas
7.	Could the working relationship with the PIP have been improved?	
8.	How has the new service affected your relationship with your patients?	• New contributions<br>• New problems
9.	How has the new service affected your relationship with the care home staff?	• New contributions<br>• New problems
10.	Where did communication issues arise between you and the other team members of the team involved in the PIP service, and what were they?	• PIP<br>• Care Home<br>• Community Pharmacist<br>• Primary care Pharmacist<br>• District Nurse<br>• GP Practice
11.	How do you think any communication issues may have affected patient care?	• (Positively/ negatively)<br>• examples
Part 4	Acceptability	
12.	What aspects of the PIP service went well from your perspective?	• Time commitment<br>• Paper work<br>• Issues raised/resolved
13.	What aspects of the PIP service went less well from your perspective?	• Time commitment<br>• Paper work

For peer review only - http://bmjopen.bmj.com/site/about/guidelines.xhtml

	**Stem question**	**Probes / follow ups**
		• Issues raised/resolved
	How could we improve these?	
14.	What elements of the service did you like best?	• Patient care
15.	Overall, how satisfied are you with the service provided by the PIP?	
16.	Would you like the service to continue?	• In its current from<br>• In a revised form<br>• Not at all
17.	Any final comments?	
18.	Thank you for taking part in this interview	

For peer review only

For peer review only - http://bmjopen.bmj.com/site/about/guidelines.xhtml

CHIPPS WP6 QUAL Evaluation: Topic Guide for Care Home Manager

	Stem question	Probes / follow ups
Part 1	Views of the intervention	
1.	Overall what are your views of the PIP service?	• New contributions<br>• New problems
	Views on changing skill mix in the primary care team<br>Views on changing skills mix in the care home team	• Diversity in team(s)<br>• Challenges<br>• Issues in managing
2.	How has the PIP service impacted on your work load? (positively/negatively)	• Time<br>• Referrals<br>• Tests<br>• Medication changes<br>• Medication reviews<br>• Repeat prescriptions<br>• Medication administration processes<br>• Record keeping<br>• Medication storage<br>• Interactions with GPs<br>• Interaction with community pharmacist<br>• Number of pharmaceutical advisory visits (i.e. not including PIP)
3.	In what ways has the PIP service affected patient care? positively/negatively)	• Examples<br>• Acceptability for patients without capacity?

For peer review only - http://bmjopen.bmj.com/site/about/guidelines.xhtml

	Stem question	Probes / follow ups
4.	Have you or your staff received any additional training since the introduction of the PIP service?	• Impact?
Part 2	Implementation	
5.	What barriers if any to implementing this service?	
	How could we overcome any barriers?	
6.	What did you see as facilitating the service to be implemented?	
Part 3	Working relationship	
7.	What is your view of your working relationship with the PIP?	• Good relationship areas<br>• Difficult relationship areas
	How can your working relationship with the PIP be improved?	
8.	What is your view of your staffs' relationship with the PIP?	• Good relationship areas<br>• Difficult relationship areas
	How can their working relationship with the PIP be improved?	
9.	How has your working relationship with the PIP affected your relationship with your patients?	• New contributions<br>New problems
10.	Where did communication issues arise between you and the other team members involved in the PIP service, and what were they?	• PIP<br>• Community Pharmacist<br>• Primary care Pharmacist<br>• District Nurse<br>• GP Practice
11.	How do you think communication issues may have affected patient care?	• Examples
Part 4	Acceptability	
12.	What aspects of the PIP service went well from your perspective?	• Time commitment<br>• Paper work<br>• Issues raised/resolved
13.	What aspects of the PIP service went less well from your perspective?	• Time commitment

For peer review only - http://bmjopen.bmj.com/site/about/guidelines.xhtml

	Stem question	Probes / follow ups
		• Paper work<br>• Issues raised/resolved
	How could we improve these?	
14.	What elements of the service did you like best?	
15.	Overall, how satisfied are you with the service provided by the PIP?	
16.	Would you like the service to continue?	• In its current from<br>• In a revised form<br>• Not at all
Part 5	Study procedures	
17.	Recruitment and participation	• Acceptability for residents with/without capacity<br>• Time commitment<br>• Paper work
18.	Any final comments	
19.	Thank you for taking part in this interview	

For peer review only - http://bmjopen.bmj.com/site/about/guidelines.xhtml

CHIPPS Care Homes Independent Pharmacist Prescribing Study

CHIPPS WP6 QUAL Evaluation: Topic Guide for Care Home Staff

	**Stem question**	**Probes / follow ups**
Part 1	Views of the intervention	
1.	What involvement have you had with the PIP service?	
2.	Overall what are your views of the PIP service?	• New contributions<br>• New problems
	Views on changing skill mix in the primary care team<br>Views on changing skills mix in the care home team	• Diversity in team(s)<br>• Challenges<br>• Issues in managing
3.	How has the PIP service impacted on your work load? (positively/negatively)	• Time<br>• Referrals<br>• Tests<br>• Medication changes<br>• Medication reviews<br>• Repeat prescriptions<br>• Medication administration processes<br>• Record keeping<br>• Medication storage<br>• Interactions with GPs<br>• Interaction with community pharmacist
4.	In what ways has the PIP service affected patient care? positively/negatively)	• Examples<br>• Acceptability for patients without capacity?
5.	Have you received any additional training since the introduction of the PIP service?	• Impact?
Part 2	Implementation	

For peer review only - http://bmjopen.bmj.com/site/about/guidelines.xhtml

	Stem question	Probes / follow ups
6.	What barriers if any to implementing this service?	
	How could we overcome any barriers?	
7.	What did you see as facilitating the service to be implemented?	
Part 3	Working relationship	
8.	What is your view of your working relationship with the PIP?	• Good relationship areas<br>• Difficult relationship areas
9.	*How can your working relationship with the PIP be improved?*	
10.	How has your working relationship with the PIP affected your relationship with your patients?	• New contributions<br>New problems
11.	Where did communication issues arise between you and the other team members involved in the PIP service, and what were they?	• PIP<br>• Community Pharmacist<br>• Primary care Pharmacist<br>• District Nurse<br>• GP Practice
12.	How do you think communication may have affected patient care? (positively/ negatively)	• Examples
Part 4.	Acceptability	
13.	What aspects of the PIP service went well from your perspective?	• Time commitment<br>• Paper work<br>• Issues raised/resolved
14.	What aspects of the PIP service went less well from your perspective?	• Time commitment<br>• Paper work<br>• Issues raised/resolved
	How could we improve these?	
15.	What elements of the service did you like best?	• Patient care

For peer review only - http://bmjopen.bmj.com/site/about/guidelines.xhtml

	**Stem question**	**Probes / follow ups**
16.	Overall, how satisfied are you with the service provided by the PIP?	
17.	Would you like the service to continue?	• In its current from<br>• In a revised form<br>• Not at all
18.	Any final comments?	
19.	Thank you for taking part in this interview	

For peer review only

For peer review only - http://bmjopen.bmj.com/site/about/guidelines.xhtml

CHIPPS WP6 QUAL Evaluation: Topic guide for residents or relatives

	**Stem question Resident/relatives**	**Probes / follow ups**
Part 1 Awareness of the intervention		
1.	Overall have you noticed any changes in your care/ the care of your relative/friend since the introduction of the PIP service?	
	What changes have you noticed?	• Good/Bad<br>• Examples
Part 2 Implementation		
2.	Before the PIP service started, what did you think about the information you received describing the new service?	• Relevant to you<br>• Amount of detail<br>• Understandable
3.	Given information you had before the PIP service started, how far was the service what you thought it would be?	• Examples
Part 3 Relationship with PIP		
4.	How did you get on with your PIP?	• Good relationship areas<br>• Difficult relationship areas
	How could your relationship with the PIP be improved?	
5.	How easy did you find it to talk to the PIP?	• Examples
6.	How do you think your relationship with the PIP affect your care/ your relative/friends care?	• Examples
Part 4 Acceptability		
7.	What aspects of the PIP service went well from your perspective?	• Examples
8.	What aspects of the PIP service went less well from your perspective?	• Examples
9.	Is there anything we could improve the overall service?	• Examples
10.	Overall how satisfied are you with the service provided by the PIP?	

For peer review only - http://bmjopen.bmj.com/site/about/guidelines.xhtml

Part 5	Study procedures	
16.	Recruitment and participation	• Acceptability for residents without capacity
11.	Any final comments?	
12.	Thank you for taking part in this interview	

End document

For peer review only - http://bmjopen.bmj.com/site/about/guidelines.xhtml

Supplementary file 3 Coding Framework

code	Number participants	Number times coded
1 Implementation	10	30
list of services offered by PIP	5	7
care home study procedure	8	23
GP study procedures	6	7
2 Mechanisms of impact	7	46
medication changes made	13	33
bio chemical monitoring	8	9
non-patient facing activities	8	25
completing PCP	5	15
tasking items to GP	4	4
liaising with community pharmacist about scripts	7	14
liaising with primary care practice pharmacy staff	4	6
evidence of GP tasking items to PIP	4	10
recruiting residents	3	4
authorising repeat prescription	7	13
staff education	8	13
staff training	12	15
review of medication systems	11	18
review of stock	7	8
care home workload due to intervention	7	24
3 Communication	10	62
PIP to GP	9	19
PIP to care home	12	55
PIP to residents and relatives	8	16
PIP to others	10	21
GP to PIP	7	16
Care home to PIP	13	56
resident or relative to PIP	0	0
GP to CH	5	6
4 Outcomes	10	46
case studies of improvement to residents	18	43
case studies where planned change not successful	2	2
reduced drug or stock wastage	3	3
improved ordering system	8	19
adding to multi disciplinary practice	17	44
opinion on intervention	6	32

For peer review only

PIP satisfaction or not	5	11
GP satisfaction or not	7	28
Care home satisfaction or not	10	47
5 intervention safety	4	6
PIP perspective	5	12
GP perspective	7	17
Care home perspective	6	9
resident or relative perspective	0	0
6 contextual factors	13	61
barriers to delivery	18	44
facilitators to delivery	14	42
impact of site factors on delivery	14	34
impact of patient factors on delivery	8	12
GP workload impact	12	27
care home staff awareness	11	30
7 Intervention normalised into routine	13	25
actions taken to ensure intervention works	3	5
narrative of engagement or disengagement	10	18
legacy of intervention	11	20
Future developments	2	4
8 Discussion on professional roles	12	46
9. Geographic differences	6	9
10 Should this type of service be implemented	14	21

For peer review only

Supplementary file 4 Example of text to code 'PIP satisfaction or not'

PIP 1

Reference 1-the one to one contact, the face to face umm for those patients that were able to participate umm you know I think a lot of the time they are happy to see somebody and know there is another person involved in their care, umm a lot of them were great characters and you know it was lovely for building that relationship and then of course there would be the patients where you would be in a relationship with them and then you would be leaving the room and they had forgotten who you were, that was just their you know their conditions, so umm but no it was a positive experience definitely from the patients' perspective and getting to know them.

Reference 2 - I thought the project is really worthwhile umm and I hope that you are able to get all the data and make sense of it all especially with me, umm I, you know I think it's a great project, I have encouraged other people to get involved in it locally umm and you know I know there's a few. -

PIP 2

Reference 1 - part of me, admittedly four hours isn't enough time, sometimes I wish it was more patients because I felt I could have probably made more of an impact and I do wish it was only the one Care Home but other than that.

Reference 2 – I think so with the Primary Care network role, pharmacist's role that is something that each Care Home resident will be getting and should be getting the Pharmacist's review so. Yeah it is, it's utterly definitely of benefit. - §

PIP 6

Reference 1 – Q I'm just wondering if there is a specific example of where you felt that that lack of knowing affected your sort of practice? Response I think it just was the, I think it was just the fact that I never got to the stage where the Home were calling me about things, we never got to that stage

Reference 2 – Q Was there anything that was particularly good about your experience of delivering the service? Response 2 / 3 Definitely, yes, I have been very negative, no I think just the fact that there is a need for it, you know I did pick up on things in review, GPs now are so busy you know particularly when they get given, the way that it happens, that they get given a whole Care Home to look after, they do tend to when, you know once that patient moves into that Care Home the GP will you know do a quick review and all of that to get to know them and their records a bit better but often after that the GP can only ever respond to acute needs and those patients, you know often those, the way it works at the Care Home I was at, that the GP would visit every Thursday, they would have a list of all of the patients that the Care Home wanted them to see so it was often dictated by what the Care Home needs were rather than what the GP, and by that time it was all filled up so the GP you know would follow up on some things but in terms of on-going chronic conditions that's where I feel Pharmacists in Care Homes have an impact and that I saw, so my main things were about trying to reduce pain medication, so we got a few people reduced on some of the Opioid patches and monitoring, not over-monitoring because you know that is totally unnecessary at this stage but the monitoring that is necessary so there was some blood tests that had slipped that were important and de-prescribing other things for different medications like statins that patients don't need so much anymore and the GP I felt was grateful for that because they would love to have

For peer review only

the time to do that I'm sure, but yeah they are working on a, you know firefighting basis whereas this was more of a you know a pro-active approach to it. –

PIP 19

Reference 1 - it was generally positive, there were obviously some things we will perhaps come back to that were sort of challenging in terms of time management and prioritising what I needed to do umm but umm but yes on the whole I think I learnt from it and it was beneficial to the patients I hope. –

PIP 9

Reference 1 - quite good really it kind of built upon the kind of relationships that I had with the Care Home anyway, so I do speak to them quite often on the phone, I deal with queries but it just, it made things better because I had to go and see the Home, I didn't know where the Home was, it was just out there somewhere. I didn't know what the Home was like, I had a picture of it in my head about who the people were who I was dealing with but I didn't know them so I had to go and see them so that was great and then because we knew one another we then kind of shared email addresses and stuff and we made maybe some of the things a bit more twenty-first century, we were emailing one another whereas in the past they were faxing us queries and stuff so, so that's yeah so it was, yeah it was good it kind of pushed us a bit more together to kind of work together.

Reference 2 - can't think of anywhere where it was negative, I can just see it was all positive. Basically we did do a kind of an in-depth six month review of people which I don't think we would have done it had I not been involved in the CHIPPS study you know and there was lots of, because basically what I used it for we kind of took a step back and said 'why is this patient on this drug?' as opposed to 'what's the drug for?' so if they are on drugs and antidepressants you know well anti-depressants they're depressed, but actually what I started doing was going you know 'why were they put on this drug?' and sometimes we didn't know and they were on the drug and I didn't think it was doing any good and I said 'maybe we could maybe halve this or stop this?' so yeah so I can't think of anything, I can't think of anything negative.

Reference 3 - So communication only improved, you know we did move away from them sending faxes to us saying 'and you owe these medicines for various people' to them emailing and you know yeah and it just improved you know and sometimes when I had those emails I was able to reply back to them and say 'I've sorted it out for you now' but I was able to reply to the Pharmacy as well saying 'these things have been sorted, the script is on the way' or whatever and it only improved really, do you know what I mean 'improved'?

Reference 4 - I thought whatever you are going to try and show a difference in, that half my time or double my time was taken filling out paperwork when actually I did do it in half the time but you do not know and I kind of squandered some of that yeah

For peer review only

BMJ Open

What happens when pharmacist independent prescribers lead on medicine management in older people's care homes: a qualitative study

Journal:	*BMJ Open*
Manuscript ID	bmjopen-2022-068678.R2
Article Type:	Original research
Date Submitted by the Author:	01-Sep-2023
Complete List of Authors:	Birt, Linda; University of East Anglia; University of Leicester<br>Dalgarno, Lindsay; University of Glasgow<br>Poland, Fiona; University of East Anglia, School of Health Sciences<br>Wright, David; University of East Anglia; University of Leicester<br>Bond, Christine; University of Aberdeen
**Primary Subject Heading**:	Geriatric medicine
Secondary Subject Heading:	Health services research
Keywords:	PRIMARY CARE, CLINICAL PHARMACOLOGY, GERIATRIC MEDICINE, Risk management < HEALTH SERVICES ADMINISTRATION & MANAGEMENT, QUALITATIVE RESEARCH

SCHOLARONE™
Manuscripts

I, the Submitting Author has the right to grant and does grant on behalf of all authors of the Work (as defined in the below author licence), an exclusive licence and/or a non-exclusive licence for contributions from authors who are: i) UK Crown employees; ii) where BMJ has agreed a CC-BY licence shall apply, and/or iii) in accordance with the terms applicable for US Federal Government officers or employees acting as part of their official duties; on a worldwide, perpetual, irrevocable, royalty-free basis to BMJ Publishing Group Ltd ("BMJ") its licensees and where the relevant Journal is co-owned by BMJ to the co-owners of the Journal, to publish the Work in this journal and any other BMJ products and to exploit all rights, as set out in our licence.

The Submitting Author accepts and understands that any supply made under these terms is made by BMJ to the Submitting Author unless you are acting as an employee on behalf of your employer or a postgraduate student of an affiliated institution which is paying any applicable article publishing charge ("APC") for Open Access articles. Where the Submitting Author wishes to make the Work available on an Open Access basis (and intends to pay the relevant APC), the terms of reuse of such Open Access shall be governed by a Creative Commons licence – details of these licences and which Creative Commons licence will apply to this Work are set out in our licence referred to above.

Other than as permitted in any relevant BMJ Author's Self Archiving Policies, I confirm this Work has not been accepted for publication elsewhere, is not being considered for publication elsewhere and does not duplicate material already published. I confirm all authors consent to publication of this Work and authorise the granting of this licence.

For peer review only

What happens when pharmacist independent prescribers lead on medicine management in older people's care homes: a qualitative study

Linda Birt[1,2], Lindsay Dalgarno,[3,4] Fiona Poland,[1] David Wright,[5,2] Christine Bond[3]

1.School of Health Sciences, University of East Anglia

2.School of Healthcare, University of Leicester

3.Institute of Applied Health Sciences, University of Aberdeen, Aberdeen, UK

4.General Practice and Primary Care, University of Glasgow

5. School of Pharmacy, University of East Anglia

Corresponding author

Dr Linda Birt

School of Health Sciences

Queen's Building

University of East Anglia

Norwich Research Park

Norwich

NR4 7TJ

Linda.birt@uea.ac.uk

For peer review only

For peer review only - http://bmjopen.bmj.com/site/about/guidelines.xhtml

What happens when pharmacist independent prescribers lead on medicine management in older people's care homes: a qualitative study

Abstract

Objective: Older people in care homes frequently experience polypharmacy, increasing the likelihood of medicine-related burden. Pharmacists working within multidisciplinary primary care teams are ideally placed to lead on medication reviews. A randomised controlled trial placed pharmacists, with independent prescribing rights (PIPs), into older people care homes. In the intervention service PIPs worked with general practitioners (GPs) and care home staff for 6 months, to optimise medicine management at individual resident and care home level. PIP activity included stopping medicines that were no longer needed or where potential harms outweighed benefits. This analysis of qualitative data examines health and social care stakeholders' perceptions of how the service impacted on care home medicine procedures and resident wellbeing.

Design: Pragmatic research design with secondary analysis of interviews.

Setting: Primary care pharmacist intervention in older people care homes in England, Scotland and Northern Ireland.

Participants: Recruited from intervention arm of the trial: Independent Prescribing Pharmacists (n=14), General Practitioners (n=8) Care Home Managers (n=9) Care Home Staff (n=6).

Results: There were resonances between different participant groups about potential benefits to care home residents of a medicine service provided by PIPs. There were small differences in perceptions about changes related to communication between professionals. Results are reported through three themes 1) '*It's a natural fit'* – pharmacists undertaking medication review in care homes fitted within multidisciplinary care; 2) *'The resident is cared for'* - there were subjective improvements in residents' wellbeing; 3) '*Moving from "firefighting" to effective systems'* - there was evidence of changes to care home medicine procedures.

Conclusion: This study suggests that pharmacist independent prescribers in primary care working within the multi-disciplinary team can manage care home residents' medicines leading to subjective improvements in residents' well-being and medicine management procedures. Care home staff appreciated contact with a dedicated person in the GP practice.

Strengths and limitations of this study

- The study draws on data from a relevant sample of 38 health and social care professionals involved in medicine management in care homes.

For peer review only - http://bmjopen.bmj.com/site/about/guidelines.xhtml

- The analytical approach foregrounded the effect of pharmacist-led medication review on the older resident in care homes, therefore having relevance to practices supporting safer medicines in this group of people.
- A limitation is the absence of residents' voices, due to recruitment challenges with this group.
- The study provides insights into activity provided by specialist pharmacists with independent prescribing qualifications so it cannot be assumed that more generalist pharmacists would have the experience to make specialised medicine management decisions.

Key words Pharmacist independent prescriber, care home, older people, primary care, medication, general practice, multidisciplinary health care.

For peer review only

Introduction

Older people in care homes are often subject to complex medicine regimes which may include the concurrent administration of more than five different medicines daily (1,2). This is known as polypharmacy; it is associated with a higher risk of adverse side effects including falls, hospitalisation and mortality (3,4). The complexity of managing and administering multiple medicines increases care home staff workload (5). This can lead to increased rates of medicine administration errors, and while few errors result in serious consequences for residents, all need to be reported and followed up (6). The Care Quality Commission highlights management of medicines as an area of concern and holds it under constant review (7). In United Kingdom care homes, prescribing and medication reviews for individual residents are the responsibility of clinical professionals in the primary care team (8). A Structured Medication Review (SMR) is recommended for all care home residents. (9) An SMR takes a personalised approach, drawing on shared decision-making principles to increase safety and efficacy (9). Pharmacists are being routinely employed to lead on SMRs in primary care practices, and an English government initiative to enable pharmacists to have a role within care homes is gathering momentum (10). Pharmacists have specialised knowledge of medicine-related burden (11) including 'drug burden' where the cumulative effect of the active ingredients of medicines can impact on physical and cognitive function (12). If pharmacists have the postgraduate Independent Prescriber qualification, they can start (prescribe) or stop (deprescribe) medicines within their area of clinical competence (13,14). This enhanced role allows them to use their expert clinical knowledge of medicines to deliver services in care homes.

This paper reports on a secondary analysis of interview data collected from pharmacists with independent prescribing rights (PIPs), general practitioners (GPs) and care home (CH) staff as part of the process evaluation (15) undertaken as part of the Care Home Independent Prescribing Pharmacist Study (CHIPPS) (16). Within the trial a service intervention was designed which enabled pharmacist-led medicine management for care homes. PIPs worked within care homes on a weekly basis over 6 months to review their medicine systems and residents' personal medicines (17). PIPs were based within the general practice aligned to the care home. The secondary analysis aimed to understand how the PIP role was enacted and PIPs, GPs and CH staffs' perceptions of how PIP activity impacted on care home medicine procedures and resident wellbeing, concepts which were outside the remit of the trial process evaluation (17).

Methods

The CHIPPS study

The pharmacist-led service offered in care homes is briefly described below to set the findings of the secondary analysis in the practice context; full details of the trial design are available at (16,17) and trial outcomes are available (18).

The pharmacist-led service was delivered through a triad of a GP, a PIP based at the GP practice, and up to 24 care home residents, from up to three care homes, registered with the GP. Service

For peer review only - http://bmjopen.bmj.com/site/about/guidelines.xhtml

specifications were developed following focus groups with stakeholders to guide the PIPs' work (see supplementary file 1). The primary trial outcome was reduction in falls for residents (18). However, in a pre-study focus group, health and social care professionals (HSCPs) wondered whether there might be wider benefits for residents (19). The communication between the triad, their understanding of responsibilities of PIPs and outcomes for residents are the focus of this secondary analysis of process evaluation interview data. Within the trial 25 triads were recruited to the intervention arm. Sixteen PIPs were already working with the GP; the remaining nine were allocated a GP practice for the purposes of the study. PIPs undertook a two-day bespoke face-to-face training programme, produced a portfolio of evidence of competencies and finally were signed off to prescribe in older people medicine by an independent GP (20). To spread researcher workload (triad recruitment, data collection) the intervention was delivered over four six-month phases between January 2018 and March 2020. Data collection for the process evaluation occurred between May 2019 and March 2020 as each phase completed. The secondary analysis of interview data was carried out by LB and LD.

Design

Pragmatism is a research methodology suited to characterising knowledge about how behaviours and actions impact on healthcare systems and outcomes. This approach can help appreciate the value of knowledge within its context of activity, uses and how these relate to the experience of addressing practical problems (21). As this work aimed to understand and learn from the diverse ways different stakeholders engaged with and perceived the intervention, a pragmatic design provided a way to recognise multiple realities of people involved. English ethical approval was gained from East of England Cambridge Central Research Ethics Committee 17/EE/0360 (28.11.2017); this applied to research in Northern Ireland. Scottish ethical approval was gained from Scotland A Research Ethics Committee 17/SS/0118 (07.12.2017). Written informed consent was obtained from all subjects.

Recruitment and sample

During the process evaluation, health and social care stakeholders in each triad where the intervention was delivered, were invited to take part in a semi-structured interview at the end of each 6-month phase: PIPs n=23 (2 withdrew and contact details not available) GPs n=25, CH managers n=38. A reminder invitation email was sent after two weeks. CH managers provided recruitment information to staff, residents and relatives. Those interested in participating returned expressions of interest to study researchers. A purposive sampling framework was designed to ensure representation across stakeholder roles, location and phases of the intervention. However, response rates were low and therefore all those replying were invited to interview.

Data collection

For peer review only - http://bmjopen.bmj.com/site/about/guidelines.xhtml

The process evaluation semi-structured interview guide drew on outcomes from the earlier feasibility study (22) and reflected the Medical Research Council process evaluation domains (23). Questions explored participants' experiences of the service intervention, anticipated and unanticipated outcomes, including multidisciplinary communication and outcomes for residents (see supplementary file 2). Interviews were undertaken by LB and LD academic researchers, between May 2019 and March 2020, either in person or by telephone and audio recorded and professionally transcribed. Informed consent was obtained before interview.

Data management and analysis

An inductive thematic analysis was used to characterise participants' experiences of delivering or receiving the pharmacist-led service. Thematic analysis provides a structured, methodical way for the researcher to familiarise with the data and to organise, analyse and report findings; importantly it is not strongly aligned to any epistemological stance (24) so was appropriate within the pragmatic design. Both researchers familiarised themselves with the data through checking transcripts for accuracy, then reading and making analytical memos (25). Next, they independently developed open codes (see supplementary file 3). The developing framework was discussed with the wider research team to refine the boundaries of the codes. Each interview was coded and any uncertainty arising during coding was discussed between researchers (see supplementary file 4). While the researchers predominantly coded interviews they had carried out, each coded two of the other's interviews to check for consistency of approach. This along with fortnightly review meetings increased dependability during analysis. The coding framework was revised iteratively as analyses progressed, for example grouping similar codes into a category of meaning. Categories were refined and, following discussions with the research team and patient and public partners, the final themes (overarching, abstracted semantic interpretations of the data (26)) were developed. While a theme does not rely on specific quantities of data, here each theme provided extensive examples of data. To further support trustworthiness in results, illustrative examples of data within the theme were shared at research meetings to seek consensus from healthcare professionals, including pharmacists and general practitioners. Participant validation was not undertaken due to the time between interviews conducted in each phase and the end of study analysis.

Patient and Public Involvement

Public involvement was supported by the Patient and Public Involvement in Research group (further details at https://nspccro.nihr.ac.uk/working-with-us/public-patient-and-carer-voice-in-research). Public members were involved at all stages of the trial and in relation to this paper they reviewed data and provided comments on emerging interpretations. Public members had experience of having relatives receiving polypharmacy and of working in care homes; this enabled the research team to have confidence in their interpretations and to consider in greater detail potential resident-focused benefits.

Results

For peer review only - http://bmjopen.bmj.com/site/about/guidelines.xhtml

Interviews, lasting between 30 and 90 minutes, were undertaken with 38 HCPs: 14 PIPs, 9 GPs, 9 CH Managers and 6 CH staff. Participants were recruited from 18 of the 25 triads: 6 in Scotland, 4 in Northern Ireland and 8 in England. There was no expression of interest from any stakeholder in seven triads. In three of these triads the PIPs did not deliver the intervention; in the remaining four triads the demographics and trial outcomes were similar to the sample interviewed. Data were collected from a heterogenous sample of professionals and locations as shown supplementary file 5. (demographic characteristics of interview participants). Data were collected from triads with older people care homes that provide personal care and some social activities (residential) and dual registration care homes which provide personal and nursing care. We identify illustrative quotes within quotation marks. For substantial quotes we provide professional role and unique triad number; non-italicised text in quotes is for explanation.

Three themes were developed. Theme 1) *'It's a natural fit' - multidisciplinary working in care homes* explores GP, PIP and care home staff differing experiences of this new role; 2) *'The resident is cared for' - shared goals in medicine management* provides exemplars of improvement in residents' experience of medication and the impact of targeted deprescribing; 3) *'Moving from "firefighting" to effective systems'* reports on the improvement to organisation systems across care homes and GP practices and whether this lasted beyond the intervention. Participants mostly recounted positive experiences but some identified challenges inherent in pharmacist-led medicine management in care homes.

Theme 1 'It's a natural fit'- multidisciplinary working in care homes

This theme reports GP, care home staff and PIPs' experiences of pharmacist-led medicine management. The GP and care home staff valued the PIPs' activity for differing reasons, suggesting a PIP role can meet both sets of needs.

GP perceptions

GPs highlighted the safety advantage of having PIPs within the multidisciplinary team, explaining that it was helpful to have 'another pair of eyes' to increase patient safety:

> *'Having a pharmacist who had good knowledge of all kinds of medications, going through polypharmacy, with a fine-tooth comb and picking up any errors or things we could do better.* GP_21.

GPs also drew on PIPs' expertise of medicine administration for the wider benefit of care home residents, *'things that can be crushed or can be opened or whether they need to be changed to suspensions'*, explaining the PIP '*seems to know the answer for everything with regards to medication interactions and probably the thing I ask her most is appliances, different catheters and things*' GP_8.

GPs explained that as PIPs could spend several hours within the home with a small number of residents, they could understand residents' medicine needs in greater detail, '*it has brought up*

things that I might not have thought about' GP_16. GPs and care home staff were generally open to suggestions from PIPs. However, two PIPs not based in the GP practice prior to the intervention struggled to have their prescribing role accepted, *'I couldn't just go off and prescribe things because the doctor would need to know … it was quite obvious she didn't want me to do anything without putting it through her'* PIP_19. This may suggest a need for actively reviewing and clarifying accountability of roles so that PIPs may be fully integrated into the multidisciplinary team.

While GPs found it hard to quantify the impact of the intervention on their workload, on reflection they mostly described how they were now dealing with fewer daily enquiries from care homes. Where PIPs and GPs had established communication channels, the PIPs regularly identified residents and issues that the GP should look at in their weekly ward round:

> *'It has reduced the time taken to see patients as I can be confident their meds are all up to date, tests required for routine monitoring have been flagged up and I have been able to action these'* GP_3.

Not all GPs agreed that the PIP intervention was a 'natural fit', rather one GP described a situation where they believed the intervention had increased their workload as the PIP had requested biochemical monitoring which was *'creating work…actual fact I didn't change resident's dose, there was no indication to do so and I did wonder what I was trying to achieve.'* GP_19. In part this situation arose as the PIP did not have full access to the resident's clinical records and was unaware a blood test had been taken during a recent hospital visit, highlighting the importance of PIPs being able to have full access to the primary care team's systems and patient records.

Care home team perceptions

Care home managers and staff identified benefits in having a dedicated pharmacist attached to the home. Most care home teams explained they had gained improved accessibility and advice by having PIPs as a point of contact in the primary care team, *'if you're phoning a surgery about a pharmacy issue, probably better speaking to a pharmacist than a GP'* CH manager_21a.

Where multidisciplinary working was embedded, care home teams explained the clinical skills of each professional were respected, and teamwork supported effective resident care:

> *'The* [CH] *nurses' assessments are being taken as a valuable tool, because the nurses are observing, the nurses are giving the assessment, and the nurses are liaising with the PIP, to prescribe what they think is needed, so I think it's a win, win situation, the nurses are feeling valued, and the PIP as well'* CH manager_21b.

However, in two homes PIPs were not able to become integrated into the care home. In one the PIP reported that care home staff were unclear of their role. In another there was confusion about the legal position of PIPs to prescribe and the manager insisted the GP signed off all the PIP's prescribing activity. This highlights the importance of understanding and trust across the triad.

PIP perceptions

For peer review only - http://bmjopen.bmj.com/site/about/guidelines.xhtml

PIPs commonly reported that their prescribing authority meant decisions and resident care could be followed up in a timely way:

> *'I think the benefits of having pharmacists in Primary Care is we can go in and make the changes and own it and follow it up rather than pass it on, …leaving it for other people to follow up'* PIP_17.

A few PIPs mentioned the role of the pharmacy technician as potentially supporting their work, as pharmacy technicians would undertake stock control and check medicine administration records for accuracy with stock. Only one PIP appeared to have worked with a technician during the intervention '*…a [pharmacy] technician that's employed to go around the homes, I had a relationship with them already, … a lot of the work that they had already done, …so it meant me going in wasn't as huge job to turn around the whole care home'* PIP_14. A GP pointed out that it may not be good use of a pharmacist '*to be going in and counting tablets and working out what the home needed'* GP_21.

In summary GPs and PIPs made clear what expertise an independent prescribing pharmacist could bring to the multidisciplinary team and saw a continuing place for pharmacists in care homes. While most care home teams valued the PIP role a few were still uncertain on the range of their specific responsibilities.

Theme 2 'The resident is cared for' - shared goals in medicine management

GPs and PIPs highlighted a strength of having a PIP as 'the linchpin in medicine management' in that they had detailed pharmaceutical knowledge allowing them to take actions which would directly and specifically benefit residents. PIPs reviewed prescriptions and could actively seek to rectify any prescribing errors and reduce the drug burden related to adverse effects from medicines. By doing this they importantly improved safety for the resident and helped to make MAR (Medication Administration Record) sheets fit for purpose thereby potentially reducing the risk of administration error. Changes PIPs could make ranged from small actions such as changing the time of eye drop administration, so residents were not woken every night by staff, changing tablet preparations to soluble form, to complex titrating of-medicines, for example, reducing doses of morphine-based painkillers and stopping antipsychotic medicines. PIPs reviewed all medicines in the context of a resident's current biomedical markers and stage of life.

Residents, alongside some members of the multidisciplinary team, were sometimes uncertain of the PIP's credentials, and PIPs reported that a few residents were reluctant to have their medicines reviewed, preferring to remain with prescriptions given by the GP or secondary care team. There were a few triads where care home teams struggled to recall changes which had impacted on residents' wellbeing. These proved to be either homes where PIPs struggled to build working relationships and therefore their medication activity was negligible, or homes that already had highly developed relationships with either the PIP allocated to the intervention or with another pharmacist. In this latter case it was reported that most residents already received regular

For peer review only - http://bmjopen.bmj.com/site/about/guidelines.xhtml

medication reviews.

Managing resident prescriptions

Independent prescribing activity was a key role of PIPs in the multidisciplinary team. Their involvement could directly benefit residents, often because this could progress a new prescription much more speedily:

> *'If my residents are feeling their symptoms, then refer to the PIP, and the PIP will prescribe immediately, there will immediate response, and then the residents will be happy'* CH manager_21b.

Other care home teams identified how having direct contact with a PIP stopped conditions worsening, '*somebody having dry skin, and being able to contact a pharmacist, definitely improved care and stopped things becoming more acute, skin breaking down'* CH Manager_16. They explained that for such conditions-they would not have contacted the GP but would have waited for the weekly GP visit, with the ensuing delay potentially leading to the worsening of conditions.

Key to successful deprescribing, reducing or stopping a medicine, was the PIP having trustful relationships with the care home team, the GP and the resident or their family. The importance of this trustful relationship was made evident when care home teams explained how they were now confident in trying to titrate prescriptions down as they knew the PIP was easy to contact and had agreed that if there were adverse effects the process could be stopped, and the medicine reinstated:

> *'Seeing PIP on a weekly basis meant we were able to make some huge reductions but also we were both very honest with each other so when we had got rid of almost everything and then resident's behaviour got worse again, we were able to add in a tiny little bit of something and I mean tiny bit which was appropriate'* CH Manager_9.

Titrating antipsychotic medicine is a complex process and each stakeholder needs to be fully committed and in good communication with each other. Here a PIP explains how the process continued after the intervention ended, illustrating the continued benefit of having PIPs working in the GP practice:

> *'We had started to titrate it down and for a few days they were okay and then they started to need a lot of diazepam so we increased it back up but now it has come back down by half; they are checking and watching even though the study has ended because I have been involved and generally keeping an eye on things too they still have my contacts. PIP_1.*

Discussions on deprescribing were also enabled between PIPs and the resident or their relative, thereby providing more patient-centred care. A care home manager explains:

> *'…talking through that with the PIP and the resident she has come full circle and is off her Butec patches with no pain; that was agreed with her and her family … she didn't mind coming off and giving it a trial period'* CH Manager_6.

For peer review only - http://bmjopen.bmj.com/site/about/guidelines.xhtml

Shared decisions

While it was intended that PIPs would be actively engaged with residents as part of the intervention, frequently residents did not have the mental capacity for this. However, even though a PIP stated that residents would possibly have little recall of their conversation they still found it relationally valuable to meet with residents, '*I enjoyed meeting them all and then they become more than just a name on a page, you remember the person behind the medication'* PIP_1. A few PIPs also reported the benefit of engaging with family:

> *'I could really get on well with the family, she wanted to know everything what I was going to do so that was more interesting because I could discuss things, a really good example of joint working with the family'* PIP_16.

In summary most participants readily identified ways in which the intervention had directly benefited individual residents. The key factor enabling PIPs to work in ways which benefited residents stemmed from the trust the care home team, resident and their family could place on the PIP as a readily accessible, clinically competent person who could react appropriately if adverse consequences arose to any medicine change.

Theme 3 'Moving from "firefighting" to effective systems'

Within the care home, most of the PIPs actively reviewed and where appropriate improved medicine ordering and administration systems, for example streamlining dispensing systems, consolidating MAR charts and stock control.

Streamlining medicine supply and stock control

PIPs could articulate their detailed knowledge of the role of the community pharmacist and the processes which may be in place when a medicine is started or stopped, particularly the time it can take for automated systems to register the change. This 'insider knowledge' placed PIPs in an ideal position to educate other professionals and try to streamline medicine ordering processes:

> *'Geriatrician didn't realise that although it was stopped in the MAR chart the next month's is out there in the van waiting to be delivered, just anticipating those fires'* PIP_9.

PIPs explained that such lack of understanding about the medicine supply process led to stopped medicines still being dispensed and administered and this could be a patient safety issue. The majority reported developing enhanced relationships with community pharmacists and care home staff. If the PIP worked in the GP practice, these relationships were often sustained post-intervention, *'any query we just email the PIP we get information get straight from the horse's mouth'* CH Manager_14a.

A few PIPs explained that taking part in the intervention had prompted them to review systems in the GP surgery:

For peer review only - http://bmjopen.bmj.com/site/about/guidelines.xhtml

'We have 5 people - pharmacy technician, pharmacy assistants, generating prescriptions, dealing with questions, and discharge letters, so one of them is now appointed for the nursing home patients. If we need to discuss anything, she's the one that helps if we make a change, she'll change it on their file, and then she'll liaise as well with the nursing home' PIP_22.

Rationalising record and stock

Working alongside care home staff PIPs allocated time to review MAR charts and consider if medicines could move to PRN (as required) or homely medicines. The staff reported they appreciated dedicated time to discuss things and simplified MAR charts:

'It sorted out things that we didn't need any longer and put things a bit more into place, like the PRNs particularly where some people didn't need it on their MAR charts any longer, … I suppose that had been overlooked which does happen I'm afraid, yes it was useful' CH staff_19.

Removing no-longer-needed prescriptions and identifying changes in the ordering systems helped reduce workload, *'it really reduced our workload, in terms of kind of managing medication in the nursing home'* CH manager_11.

Many of the PIPs explained that by regularly attending the care homes they were able to identify waste and unused stock and could take measures to reduce waste:

'I spoke a few times with the dispensing pharmacists about errors that came up on the MAR chart etc. we tried to address those issues. I spoke to the appliance contractors not just the Pharmacy, … I had to address it in a really decisive manner' PIP_16.

One GP highlighted the importance of cost-saving inherent in stock control, *'I wouldn't have had a clue about it* {over stock} *because I don't go into the store cupboards so that was a massive cost saving*' GP_16.

In summary, many PIPs were found to take an active lead in reviewing and advising on more efficient medicine systems. However, their efforts to improve systems could be thwarted if either the care home staff or GP team were unwilling, or unable, to adopt new ways of working.

Discussion

This secondary analysis of interview data from the process evaluation of the CHIPPS study (15) specifically focused on how the PIP role was enacted and PIPs, GPs and care home staffs' perceptions of the impact of PIP activity on care home medicine procedures and resident wellbeing. The results provide contextualised understandings of the independent prescribing pharmacist role within the wider primary care team, with most PIPs and GPs suggesting PIPs should have a continued role in management of care home medicine systems and residents' medicines. Importantly most participants reported perceptible benefits from the PIP intervention whether through improved

For peer review only - http://bmjopen.bmj.com/site/about/guidelines.xhtml

resident wellbeing or increased safety and streamlining of care home procedures. Benefits from the intervention appeared greater when there was professional trust between GP, care home staff and the PIP. Examining exceptions can help better understand what resources, training or other preparation might need to be put in place for stakeholders to benefit from extended pharmacist roles.

GPs and PIPs made clear a pharmacist independent prescriber could bring specialised clinical knowledge to the multidisciplinary team and they saw a continuing place for pharmacists in care homes. While most care home teams valued the role of the PIP a few were still uncertain on the range of their specific responsibilities. Pharmacists have an increasingly valued place within multidisciplinary primary care teams, and this is evident within policy statements (27,8). However, importantly the pharmacist role needs to cover more than reviewing medicines to maximise impact. As part of a multidisciplinary team, pharmacists can provide dedicated specialist input into Structured Medication Reviews (9) by drawing on their clinical pharmaceutical knowledge (28). A pharmacist independent prescriber may be able to relieve the GP of some prescribing activity and so help to backfill GP shortages as fewer European GPs are now recruited (29). Yet as the CHIPPS study found there are differences in the character of pharmacist medication review (30) and there are suggestions that there may need to be quality check tools on practice (31).

The review of individual resident's medicines undertaken as part of this intervention was reported by care home staff and PIPs as having readily demonstrated benefits for residents' subjective quality of life. This understanding is important as the evidence on objective improvements in resident outcomes i.e., admission to hospital, falls, or mortality from this trial and others is inconclusive (18,32,33). Holland et al., (34) suggested that measures relating to older people's quality of life may be a better measure. This is borne out in trails that fail to find significant change in primary outcomes such as falls but do find positive outcomes in reduction of drug related burden (18, 35) Our study suggests that measures of impact may need to be related to residents' everyday experiences, such as better engagement with others, more alert at mealtimes, rather than clinical risk of side effects from multiple no-longer-needed medicines. We found that there needed to be trust between the care home staff and PIPs in order for some of the pharmacists' recommendations to be enacted; care home staff needed to have confidence the pharmacist would respond quickly if a resident's behaviour became distressed and a challenge to manage.

As part of the intervention service specification PIPs spent time actively reviewing, and where appropriate, improving care home medicine systems. For example, reviewing and improving dispensing systems, consolidating MAR charts and monitoring stock control. These were reported as having benefits for care home staff in that administration and reordering were easier, and potentially safer. Improved management of stock reduced wastage and therefore had financial benefits. These findings resonate with evidence supporting the place of pharmacists within care homes (36). Nonetheless future work might explore if this is optimum use of a pharmacist's skills or if this might be a technician-level activity.

For peer review only - http://bmjopen.bmj.com/site/about/guidelines.xhtml

The exceptions to the positive results reported so far occurred when relational factors inhibited the scope of pharmacist contributions. For example, when the relationship between pharmacist and GP or care home could not be successfully established, or understanding of the pharmacist's legal clinical scope was not understood. This suggests the need for strategies which will develop shared understanding of the potential of each other's roles. Our results resonate with a survey by Kahn et al. (37) that aimed to understand factors impacting on interprofessional working in primary care; they found that the time the team had worked together, and opportunities for formal and informal communication were important in enabling the team to develop trust and collaborative working practices. In our intervention the few PIPs who had not previously worked in the trial GP surgery reported greater resistance to their role and made fewer substantive changes to residents' medicines and care home medicine ordering and administration systems. It is important that all stakeholders understand the pharmacist role so as to support collaborative working practices (38).

Strengths and limitations

Missing from our study are the resident and relatives' opinions of medication review and the place of the pharmacist in this. We attempted to recruit the residents consented to the intervention arm of the trial through care home staff. It is unclear why this was unsuccessful with only three residents expressing an interest, but in part it is likely to be due to the severe cognitive impairment many residents were living with, meaning staff may not have actively encouraged residents or their families to take part in a process evaluation interview. A further point to note is that the sample, while appearing representative of the main trial sample, consists of those who volunteered for the additional process evaluation interview so may not represent the views of those who declined. This point along with the limited number of participants represented in a small number of codes means that the transferability of the data needs to be considered with caution as the results provide insights and understandings from a very specific group of HSCPs involved in supporting older resident care homes. All pharmacists were independent prescribers, and all had received additional training in medicine for older people. It cannot be assumed that more generalist pharmacists in a GP surgery would have the experience to make the specialised medicine management decisions demonstrated by these pharmacist independent prescribers who had received additional training. There are recommendations to make care home pharmacists a designated speciality (39). However further training may be needed as inexperienced pharmacists may rely on template-driven reviews (40). The bespoke training developed for this intervention appeared appropriate for enhancing pharmacists' clinical skills and confidence (41). Pharmacists in our study had medication review responsibilities only for between 9 and 24 residents, so allowing them dedicated time to come to understand those residents' individual needs.

The CHIPPS RCT found the fall rate risk ratio for the intervention group compared with the control group was not significant. However, the Drug Burden Index outcome significantly favoured the intervention (18). Reduction in drug related problems is reported in another pharmacist led review intervention (42) indicating there is potential for pharmacist led review to have some positive

For peer review only - http://bmjopen.bmj.com/site/about/guidelines.xhtml

impacts for care home residents relating to side effects of some medicines. Further economic evaluation would be required to examine if this level of pharmacist input would be cost effective in practice. It may be that some tasks could be allocated to the developing role of pharmacy technicians (43).

There are practice implications for other roles within the wider healthcare team. For example, during interviews PIPs referred to their work with community pharmacists, describing the community pharmacist position as key in dispensing medicines. Future work exploring medicines management in care homes might include the community pharmacist or dispensing pharmacy so that the efficiency of medicine ordering and dispensing can be further optimised. This might reduce the potential safety risk, reported in this study, which occurs when medicines which have been deprescribed remain on the MAR chart.

The CHIPPS process evaluation was completed in 2020 just as primary care networks (PCN) were being introduced in England and at the start of the COVID-19 pandemic. A key aspect of the role was the presence of the pharmacy within the care home the move to more 'on-line' working since the COVID-19 pandemic may make this aspect of the intervention more difficulty to implement. Within PCNs pharmacists work across several GP practices rather than being within a single practice (44). Our finding that PIPs not integrated within the GP team faced the most challenges in developing trustful working relationships, mirrors an evaluation of PCNs published in 2022 (45,46). This evaluation reported the need for change in cultures and practices to support additional clinical roles in a GP practice (40,41). Those working within a PCN rather than a single GP surgery reported feeling a lack of autonomy, belonging and contribution, reflecting the trial experience of PIPs 'dropped' into GP practices.

Conclusion

Independent prescribing pharmacists can successfully take responsibility for medicine management and Structured Medication Reviews for older people in care homes. When pharmacists develop professional trustful relationships with GP colleagues and care home staff, they can independently make changes to medicines which benefit resident wellbeing. Their expertise in medicine systems including stock control and ordering enabled them to streamline care home systems with the potential benefit of reducing waste and likelihood of administration errors. The changing landscape of global primary care provision indicates that pharmacists will continue to have a key role in leading management of medicines but that how this happens may require monitoring to enable refinement of the delivery model.

Author contribution: DW, CB, FP conceived the overall research design and provided commentary on the progress of the data collection and analysis, LB and LD undertook qualitative data collection

For peer review only - http://bmjopen.bmj.com/site/about/guidelines.xhtml

and analysis, all authors reviewed emerging results. LB led on producing the manuscript. All authors commented on versions of the manuscript and all agree to the final version.

Acknowledgements: Thank you to residents and their families who agreed to receive the intervention, the pharmacists, GP practices and care homes who delivered the intervention and took part in the process evaluation. We would also like to acknowledge the South Norfolk Clinical Commissioning Group as the study sponsor and the CHIPPS Study team

Ethical approval: English ethical approval was gained from East of England Cambridge Central Research Ethics Committee 17/EE/0360 (28.11.2017); this applied to research in Northern Ireland. Scottish ethical approval was gained from Scotland A Research Ethics Committee 17/SS/0118 (07.12.2017). Written informed consent was obtained from all subjects.

Availability of data and materials: The datasets used and/or analysed during the current study are available from the corresponding author on reasonable request.

Competing interests: DW received speaker fees from Desitin Pharma and speaker fees and unrestricted education grants from Rosemont Pharmaceuticals. All other authors have no competing/conflict of interest.

Funding: This is a summary of independent research funded by the National Institute for Health Research (NIHR) under its Programme Grants for Applied Research Programme (Grant Reference Number RP-PG-0613-20007). The views expressed are those of the authors and not necessarily those of the National Health Service, the NIHR or the Department of Health.

For peer review only

For peer review only - http://bmjopen.bmj.com/site/about/guidelines.xhtml

References

1. MacRae C, Henderson DAG, Mercer SW, et al. Excessive polypharmacy and potentially inappropriate prescribing in 147 care homes: a cross-sectional study. *BJGP Open* 2021; 5(6): 0167. https://doi.org/10.3399/BJGPO.2021.0167
2. Pazan F, & Wehling M. Polypharmacy in older adults: a narrative review of definitions, epidemiology and consequences. *European Geriatric Medicine* 2021;12**,** 4 43–452. (https://doi.org/10.1007/s41999-021-00479-3
3. WHO. *Medication safety in polypharmacy: technical report*. 2019. Available: https://www.who.int/publications/i/item/WHO-UHC-SDS-2019.11
4. Davies LE, Spiers G, Kingston A, et al. Adverse Outcomes of Polypharmacy in Older People: Systematic Review of Reviews. *Journal of the American Medical Directors Association 2020;* 21(2):181-187. https://doi.org/10.1080/21548331.2019.1706995
5. Szczepura A, Wild D, & Nelson S. Medication administration errors for older people in long-term residential care. *BMC Geriatrics* 2011; 11(82). https://doi.org/10.1186/1471-2318-11-82
6. National Care Forum. *Preventing medication errors in care homes a review of publications.* 2019. Available:https://www.nationalcareforum.org.uk/wp-content/uploads/2019/11/Preventing-Medication-Errors.pdf
7. Care Quality Commission. *Regulation 12: safe care and treatment.* 2021. Available:https://www.cqc.org.uk/guidance-providers/regulations-enforcement/regulation-12-safe-care-treatment
8. Department of Health and Social Care. *Good for you, good for us, good for everybody A plan to reduce overprescribing to make patient care better and safer, support the NHS, and reduce carbon emissions.* 2021. Available:https://assets.publishing.service.gov.uk/government/uploads/system/uploads/attachment_data/file/1019475/good-for-you-good-for-us-good-for-everybody.pdf
9. NHS England. *Structured Medication reviews and Medicine Optimisation*. No date Available https://www.england.nhs.uk/primary-care/pharmacy/smr/
10. NHS England. *NHS England Funding and Resource 2018/19: Supporting 'Next Steps for the NHS Five Year Forward View'*. 2018. Available: NHS England » NHS England Funding and Resource 2018/19: Supporting 'Next Steps for the NHS Five Year Forward View'
11. Mohammed MA, Moles RJ, Chen TF Medication-related burden and patients' lived experience with medicine: a systematic review and metasynthesis of qualitative studies *BMJ Open* 2016; 6:e010035. doi: 10.1136/bmjopen-2015-010035
12. Kouladjian L, Gnjidic D, Chen TF, Mangoni AA, Hilmer SN. Drug Burden Index in older adults: theoretical and practical issues. *Clinical Interventions in Aging* 2014; 9:1503-1515. doi:10.2147/CIA.S66660
13. General Pharmaceutical Council. *Pharmacist Independent Prescriber*. 2022. Available: https://www.pharmacyregulation.org/education/pharmacist-independent-prescriber

For peer review only - http://bmjopen.bmj.com/site/about/guidelines.xhtml

14. Bleidt BA. Deprescribing: an emerging role for pharmacists. *Journal of Pharmaceutical Health Services Research* 2019; 10(2):159–160. https://doi.org/10.1111/jphs.12303

15. Birt L, Dalgarno L, Wright DJ, et al. Process evaluation for the Care Homes Independent Pharmacist Prescriber Study (CHIPPS). *BMC Health Serv Res*. 2021;21(1):1041. Published 2021 Oct 2. doi:10.1186/s12913-021-07062-3

16. Bond CM, Holland R, Alldred DP, et al. Protocol for a cluster randomised controlled trial to determine the effectiveness and cost-effectiveness of independent pharmacist prescribing in care homes: the CHIPPS study. *Trials* 2020, 21(1):103. https://doi.org/10.1186/s13063-019-3827-0

17. Bond CM, Holland R, Alldred DP, et al. Protocol for the process evaluation of a cluster randomised controlled trial to determine the effectiveness and cost-effectiveness of independent pharmacist prescribing in care home: the CHIPPS study. *Trials* 2020; 21(1):439. https://doi.org/10.1186/s13063-020-04264-8

18. Holland R et al. The Care Homes Independent Prescribing Pharmacist Study (CHIPPS): A cluster randomised controlled trial to evaluate safety and effectiveness BMJ In press

19. Lane K, Bond C, Wright D, et al. "Everyone needs to understand each other's systems": Stakeholder views on the acceptability and viability of a Pharmacist Independent Prescriber role in care homes for older people in the UK. *Health Social Care Community* 2020; 28(5):1479–87.https://doi.org/10.1111/hsc.12970

20. Wright D J, Blyth A, Maskrey V, et al. Development and feasibility testing of an evidence-based training programme for pharmacist independent prescribers responsible for the medicines-related activities within care homes. *International Journal of Pharmacy Practice* 2021; 29(4):376-84. https://doi.org/10.1093/ijpp/riab025

21. Kelly LM, & Cordeiro M. Three principles of pragmatism for research on organizational processes. *Methodological Innovations* 2020; 13(2). https://doi.org/10.1177/2059799120937242

22. Inch J, Notman F, Bond CM, et al. The Care Home Independent Prescribing Pharmacist Study (CHIPPS)—a non-randomised feasibility study of independent pharmacist prescribing in care homes. *Pilot Feasibility Study* 2019*;* 5:89 . https://doi.org/10.1186/s40814-019-0465-

23. Moore GF, Audrey S, Barker M,. et al. Process evaluation of complex interventions: Medical Research Council Guidance. *BMJ* 2015; 350:h1258. doi:10.1136/bmj.h1258

24. Nowell LS, Norris JM, White DE, et al. Thematic Analysis: Striving to Meet the Trustworthiness Criteria. *International Journal of Qualitative Methods* 2017; 16(1). https://doi.org/10.1177%2F1609406917733847

25. Saldana J. *The coding manual for qualitative researchers*. 2021. London, Sage Publications Ltd.

26. Kieger ME, & Varpio L. Thematic analysis of qualitative data: AMEE Guide No. 131. *Medical Teacher* 2020; 42(8):846-854. https://doi.org/10.1080/0142159X.2020.1755030

27. International Pharmaceutical Federation (FIP). *FIP Regional Conference for the European Region*. 2020. The Hague: International Pharmaceutical Federation. Available: https://www.fip.org/file/4840

28. Baruth JM, Gentry MT, Rummans TA, et al. Polypharmacy in older adults: the role of the multidisciplinary team. *Hospital Practice* 2020; 48(sup1):56-62. https://doi.org/10.1080/21548331.2019.1706995

For peer review only - http://bmjopen.bmj.com/site/about/guidelines.xhtml

29. OECD. *Health at a Glance: Europe 2020 : State of Health in the EU Cycle Availability of doctors.* 2020. Available:https://www.oecd-ilibrary.org/sites/1d767767-en/index.html?itemId=/content/component/1d767767-en

30. Birt L, Dalgarno L, Wright DJ, et al. Process evaluation for the Care Homes Independent Pharmacist Prescriber Study (CHIPPS). *BMC Health Serv Res*. 2021;21(1):1041. https://doi.org/10.1186/s12913-021-07062-3

31. Patounas, M., Lau, E.T.L., Rigby, D., Chan, V. Nissen, L.M. Development and trial of an instrument to evaluate accredited pharmacists' clinical home medicines review reports in Australia. *Journal of Pharmacy Practice and Research* 2023, 53: 32-38. https://doi.org/10.1002/jppr.1829

32. Lee, SWH, Mak, VSL, Tang, YW. Pharmacist services in nursing homes: A systematic review and meta-analysis. Br J Clin Pharmacol. 2019; 85: 2668– 2688. https://doi.org/10.1111/bcp.14101

33. O'Mahony D. Optimization of Medication by Pharmacists in Older People With Multimorbidity for Improved Outcomes—Mirage or Reality? *JAMA Network Open* 2021; 4(4):e216392. doi:10.1001/jamanetworkopen.2021.6392

34. Holland R, Desborough J, Goodyer L, et al. (2008), Does pharmacist-led medication review help to reduce hospital admissions and deaths in older people? A systematic review and meta-analysis. British Journal of Clinical Pharmacology 2008; 65:303-316. https://doi.org/10.1111/j.1365-2125.2007.03071.x

35. Roughead EE., et al. Effect of an ongoing pharmacist service to reduce medicine-induced deterioration and adverse reactions in aged-care facilities (nursing homes): a multicentre, randomised controlled trial (the ReMInDAR trial), *Age and Ageing*, 2022, 51(4). https://doi.org/10.1093/ageing/afac092

36. Riordan DO, Walsh KA, Galvin R, et al. The effect of pharmacist-led interventions in optimising prescribing in older adults in primary care: A systematic review. *SAGE Open Medicine* 2016; *4*: 2050312116652568. https://doi.org/10.1177/2050312116652568

37. Khan AF, Barnsley J, Harris JK, et al. Examining the extent and factors associated with interprofessional teamwork in primary care settings. *Journal of Interprofessional Care* 2021; 36(1):52-63. https://doi.org/10.1080/13561820.2021.1874896

38. Zogas A, Gillespie C, Kleinberg F, et al. Clinical pharmacist integration into veterans' primary care: Team members perspectives. *The Journal of the American Board of Family Medicine* 2021; 34(2):320-327. https://doi.org/10.3122/jabfm.2021.02.200328

39. Burns C. *The Pharmaceutical Journal*, Recognise care home pharmacy as a specialism, says RPS Scotland;Online: DOI:10.1211/PJ.2019.20206469

40. Madden M, Mills T, Atkin K, Stewart S. Early implementation of the structured medication review in England: a qualitative study. *British Journal of General Practice* 2022; 72 (722): e641-e648. DOI: 10.3399/BJGP.2022.0014

41. Birt L, Dalgarno L, Bond CM, et al. Evaluation of a training programme for Pharmacist Independent Prescribers in a care home medicine management intervention. BMC Medical Education 2022; 22, 55. https://doi.org/10.1186/s12909-022-03575-5

For peer review only - http://bmjopen.bmj.com/site/about/guidelines.xhtml

42. Lexow M., Wernecke K., Sultzer,R. *et al.* Determine the impact of a structured pharmacist-led medication review - a controlled intervention study to optimise medication safety for residents in long-term care facilities. *BMC Geriatric* 2022, 22, 307.https://doi.org/10.1186/s12877-022-03025-3

43. Street KA. , ADJ, Taylor. A consensus building study to define the role of a 'clinical' pharmacy technician in a Primary Care Network environment in England, *International Journal of Pharmacy Practice 2023*, 31(1):62–69. https://doi.org/10.1093/ijpp/riac077

44. Baird B., Beech J. *Primary Care networks explained*. The Kings Fund November 2020. Available https://www.kingsfund.org.uk/publications/primary-care-networks-explained

45. Baird b., Lamming L., Bhatt RT., Beech J., Dale V. Integrating additional roles into PCNs. The King's Fund February 2022. Available https://www.kingsfund.org.uk/sites/default/files/2022-02/Integrating%20additional%20roles%20in%20general%20practice%20report%28web%29.pdf

46. Mills T, Madden M, Stewart D, et al. Integration of a clinical pharmacist workforce into newly forming primary care networks: a qualitatively driven, complex systems analysis. *BMJ Open* 2022;12:e066025. doi:10.1136/bmjopen-2022-066025

For peer review only

For peer review only - http://bmjopen.bmj.com/site/about/guidelines.xhtml

Additional file 1 CHIPPS Intervention Service Specification

	Specification
**Recruitment and employment of the Pharmacist Independent Prescriber (PIP)**	
	Excellent interpersonal, communication and IT skills
	Familiarity with relevant GP software systems
	Experience of providing prescribing and medicines management advice and support
	Previous experience of working in GP practice environment
	Be able to travel to site locations
	A mobile phone to be contactable for the purposes of delivering this service
	Appropriate indemnity insurance for prescribing
**PIP roles and responsibilities**	
Review each resident's medication and develop and implement a pharmaceutical care plan (essential)	Optimise prescribing ensuring clear indication and evidence base for each medication (taking into consideration national and local pathways, guidelines and formularies), informed by tools such as STOPP/START
	Minimise the potential for adverse effects
	Optimise the dose of all medication
	Co-ordinate appropriate monitoring and associated tests for all medicines and conditions

Service specification 05.08.2020

For peer review only - http://bmjopen.bmj.com/site/about/guidelines.xhtml

	Agree initial care plan with GP, care staff and resident (where appropriate)
	Document and maintain records relating to review and care plan in GP and care home records as appropriate
Prescribing (essential)	
	Authorise repeat prescriptions
	Co-ordinate appropriate monitoring and associated tests for all medicines and conditions
	Deprescribe medicines according to agreed pharmaceutical care plan
	Document medication changes in GP and care home records and notify supplying pharmacy of all changes to medication within 24 hours
	Only initiate new medicines for existing diagnoses or for common ailments which can be managed with medicines classified by the Medicines and Healthcare products Regulatory Agency (MHRA) as Pharmacy (P) or General Sales List (GSL)
	Any additional areas of prescribing must be agreed and documented with the GP practice prior to prescribing (e.g. antibiotics for simple UTIs
Communication (essential)	
	Agree local protocols for communication with GP practice and care home prior to commencing service. This should include:<br>○ Process of communication and messaging

For peer review only - http://bmjopen.bmj.com/site/about/guidelines.xhtml

	○ The location and expected level of detail of all PIP interventions in the medical records Process and communication of referrals for activities outside the competence of the PIP
	Inform supplying community pharmacy about service and role (prior start of service)<br>○ Communicate all changes in medication to supplying pharmacy
	Complete all documentation and recording of activities as required by the study team.
Support systematic ordering, prescribing, and administration processes with each care home, GP practice and supplying pharmacy where needed: (undertaken at PIP's discretion)	
	Provide instructions on how to administer each drug
	Synchronise residents prescription quantities for monthly cycles
	Add or clarify directions for all medication where it is currently not clear
	Provide advice on repeat prescription ordering processes to:<br>○ Minimising missed items<br>○ Optimising quantities
	Optimise the use of homely remedies within the care home
	Reconcile resident medication following a transfer of care
Training provision (undertaken at PIP's discretion)	
	Review training needs of care home and GP practice and draft proposed training plan

For peer review only - http://bmjopen.bmj.com/site/about/guidelines.xhtml

	Provide training to care home staff on training needs basis from agreed list of potential topics/areas
	Provide guidance to relevant GP practice on training needs basis from agreed list of potential topics/areas
**Safe and effective service provision**	
	PIP will be contactable and respond to messages within 24 hours (Monday - Friday)
	The PIP will establish a locally agreed protocol with the GP practice for referral/notification of all medicine related queries from CHIPPS participants to the PIP as appropriate (see 4.3.5)
	PIP will have full (read/write) access to GP record system to issues prescriptions and update records
	Where possible PIP will use remote access to update records when changes are made to GP held record<br>○ Where remote access is not feasible the PIP must update records within 24 hours of making a change
	PIP will have full (read/write) access to care home records to update records during all visits using appropriate local reporting systems
	The PIP will visit/contact the care home at least once a week
	The PIP will visit/contact the GP practice at least once a week
	Wherever possible, all annual leave should be agreed before the beginning of the study. A clear system for transfer of responsibility communicated to GP, care home and supplying pharmacy

Service specification 05.08.2020

For peer review only - http://bmjopen.bmj.com/site/about/guidelines.xhtml

	The PIP will work within the local prescribing formularies of GP practice and primary care organisation.
	The PIP will report and document all significant clinical events or near misses using local reporting procedures and study documentation.
	Ensure all records are aligned

For peer review only

For peer review only - http://bmjopen.bmj.com/site/about/guidelines.xhtml

CHIPPS WP6 QUAL Evaluation: Topic Guide for PIP

As the purpose of this interview is to encourage a conversation in which the participant can feel confident in expressing their own views, the following topic guide is indicative. This means that the interviewer may adapt it to suit the conversation style and preferences of the participant.

	**Introduction.**<br>**1. Introduce yourself**<br>**2. Explain the purpose of the research**	We have developed a new service in which a specially trained 'pharmacist independent prescriber' (PIP) becomes part of the care home team, working alongside general practitioners. The aim is to improve how resident's medicines are managed in a safe, effective and cost effective way. We hope this may improve resident's care, wellbeing and health outcomes. We are evaluating this new service in a large multi-centre randomised controlled trial in different places in the United Kingdom.<br><br>The study is run by the universities of East Anglia, Aberdeen, Leeds and Queen's Belfast. It is funded by the UK National Institute for Health Research.<br><br>*Since participating in the training for 'pharmacist independent prescribers (PIPs) ' you have set up and have delivered the Care Home Independent Pharmacists Prescriber service in the trial,*<br><br>*We would like you to consider your overall role as a PIP providing this service and your experience in delivering this. We would also like your views on the Service Specification and Pharmaceutical Care Plan, your thoughts about the mentoring you received and how useful the personal development plan and training was in preparing you for this role and how this has impacted on your ability to set up and deliver this service.*
	**The digital recorder**<br>**1. Stress confidentiality**	I would like to highlight the confidentiality of everything you tell me, and specifically that:<br>• *the recording will be deleted after being transcribed*<br>• *you won't be identified individually in any report.*<br>• *all information will be anonymised.*<br>• *we will not tell anyone else including your employer organisation, what you tell us as an individual.*<br>• *likewise no other individual e.g. GP, care home staff, resident will be identifiable in any report.*

For peer review only - http://bmjopen.bmj.com/site/about/guidelines.xhtml

	**2. Set ground rules**	*We do need to remind you, however, that* if you do disclose anything which might identify a risk to yourself or to others, or a personal or professional offence, this would be shared with the relevant responsible authority. However we would tell you if we thought this were the case.<br><br>• All of your views are of value to us. There are no right or wrong answers,<br>• Please ask me to clarify if the question isn't clear.<br>• We remind you not to share any personal or patient identifying information during this interview
	**Ask if there are any questions**	
	**Confirm consent**	

	**Stem question PIP service delivery**	**Probes / follow ups**
1.	What was your experience of delivering the service?	• Any particular issues<br>• Anything particularly good<br>(Interaction with GP/ care home staff/residents/ relatives access to records/ information/routines /types of interventions)<br>• Acceptability to residents without capacity
2	In what ways did your service affect resident care	• Examples positive and negative
3.	What are your views on the Service Specification?	• Clarity?<br>• Level of detail?<br>• Inclusion/exclusion criteria<br>• Service requirements?<br>• Outcomes<br>• Transitional arrangements
	Stem Question –research activity	•
34	What are your views on the research study documentation including: Pharmaceutical Care Plan, PIP log and resource use data?	• Ease of use<br>• Anything missing/ not needed<br>• Fit for purpose?

For peer review only - http://bmjopen.bmj.com/site/about/guidelines.xhtml

CHIPPS Care Homes Independent Pharmacist Prescribing Study

		• How much time did it take
		•
5.	What were your thoughts on the role of the mentor?	• Positive<br>• Negative<br>• Areas for improvements
6.	What was your experience of developing your own personal development plan with your mentor?	• Went well/ not so well<br>• Mentor support/ time scale for completion<br>• Accessing training on the identified development areas<br>• General acceptability
7.	What do you think about the competency framework assessment?	• Comprehensive?<br>• Easy/hard to demonstrate achievement?
78	What do you think about the assessor signing you off?	• Useful / difficult discussion?<br>• Appropriate?<br>• Alternative suggestions?<br>• Process fit for purpose?
	**<u>Stem question Training</u>**	**<u>Probes / follow ups</u>**
89	What are your thoughts on the impact of training you received?	Any gaps-<br>• *preparation for role,*<br>• *underpinning knowledge,*<br>• *competency framework,*<br>• *relationship building*<br>• *personal development skills*
10.	Now that you have been delivering the service for 3 months which particular elements of the training do you think have been most useful?	• Why?<br>• Relevance<br>• Time spent on this<br>• Delivery
11.	Which particular elements of the training do you think have been least useful in your role, delivering the service?	• Should we continue to provide this element of the training?

For peer review only - http://bmjopen.bmj.com/site/about/guidelines.xhtml

		• How should this element of training be delivered?<br>• What should we do to improve these elements of training?
11.	Is there anything that should have been included in the training that wasn't?	• How would you suggest that we train pharmacists in that?<br>• Is there existing training available, that you are aware of, to meet this<br>• Need?<br>• When should this training be provided?
12.	It is important to us that across the four areas, all PIPs provide a similar service. What would be the best way for this to be achieved?	• Training?<br>• Checklist?
13.	In what ways could you contribute to the training of the next cohort of Pharmacist Independent Prescribers?	• Mentor?<br>• Helping to deliver educational package locally?
14	How important for you was peer support	• use The Telegram group<br>• Use other ways if staying in contact
	**Stem question on team work**	•
8.	Where did communication issues arise between your and the other team members involved in the PIP service?	• Care homes<br>• Other Pharmacist<br>• Primary care team<br>• GP Practice<br>• Relatives/residents
9.	How do you think communication impacted on resident care? (positively/ negatively)	• Examples
14.	Any final comments?	
15.	Thank you for taking part in this interview	

N.B. all interview topic guides started with the setting the introduction and digital record prompts; in the remainder of the document we only include stem questions.

For peer review only - http://bmjopen.bmj.com/site/about/guidelines.xhtml

CHIPPS WP6 QUAL Evaluation: Topic Guide for GP

	Stem question	Probes / follow ups
Part 1	Intervention	
1.	Overall what are your views of the PIP service?	• New contributions<br>• New problems
	Views on changing skill mix in the primary care team<br>Views on changing skills mix in the care home team	• Diversity in team(s)<br>• Challenges<br>• Issues in managing
2.	How has the PIP service impacted on your work load?	• (Negatively/ positively)<br>• Time<br>• Visit frequency<br>• Referrals<br>• Tests<br>• Medication changes<br>• Medication reviews<br>• Medication ordering<br>• Repeat prescriptions
3.	In what ways has the PIP service impacted on patient care?	• Examples<br>• Acceptability for patients without capacity?
Part 2	Implementation	
4.	What barriers if any to implementing this service?	
	How could we overcome any barriers?	
5.	What did you see as facilitating the service to be implemented?	

For peer review only - http://bmjopen.bmj.com/site/about/guidelines.xhtml

	Stem question	Probes / follow ups
	**Stem question**	**Probes / follow ups**
Part 3	Working relationship	
6.	What is your view of your working relationship with the PIP?	• Good relationship areas<br>• Difficult relationship areas
7.	Could the working relationship with the PIP have been improved?	
8.	How has the new service affected your relationship with your patients?	• New contributions<br>• New problems
9.	How has the new service affected your relationship with the care home staff?	• New contributions<br>• New problems
10.	Where did communication issues arise between you and the other team members of the team involved in the PIP service, and what were they?	• PIP<br>• Care Home<br>• Community Pharmacist<br>• Primary care Pharmacist<br>• District Nurse<br>• GP Practice
11.	How do you think any communication issues may have affected patient care?	• (Positively/ negatively)<br>• examples
Part 4	Acceptability	
12.	What aspects of the PIP service went well from your perspective?	• Time commitment<br>• Paper work<br>• Issues raised/resolved
13.	What aspects of the PIP service went less well from your perspective?	• Time commitment<br>• Paper work

For peer review only - http://bmjopen.bmj.com/site/about/guidelines.xhtml

	Stem question	Probes / follow ups
		• Issues raised/resolved
	How could we improve these?	
14.	What elements of the service did you like best?	• Patient care
15.	Overall, how satisfied are you with the service provided by the PIP?	
16.	Would you like the service to continue?	• In its current from<br>• In a revised form<br>• Not at all
17.	Any final comments?	
18.	Thank you for taking part in this interview	

For peer review only

For peer review only - http://bmjopen.bmj.com/site/about/guidelines.xhtml

CHIPPS WP6 QUAL Evaluation: Topic Guide for Care Home Manager

	Stem question	Probes / follow ups
Part 1	Views of the intervention	
1.	Overall what are your views of the PIP service?	• New contributions<br>• New problems
	Views on changing skill mix in the primary care team<br>Views on changing skills mix in the care home team	• Diversity in team(s)<br>• Challenges<br>• Issues in managing
2.	How has the PIP service impacted on your work load? (positively/negatively)	• Time<br>• Referrals<br>• Tests<br>• Medication changes<br>• Medication reviews<br>• Repeat prescriptions<br>• Medication administration processes<br>• Record keeping<br>• Medication storage<br>• Interactions with GPs<br>• Interaction with community pharmacist<br>• Number of pharmaceutical advisory visits (i.e. not including PIP)
3.	In what ways has the PIP service affected patient care? positively/negatively)	• Examples<br>• Acceptability for patients without capacity?

For peer review only - http://bmjopen.bmj.com/site/about/guidelines.xhtml

	Stem question	Probes / follow ups
4.	Have you or your staff received any additional training since the introduction of the PIP service?	• Impact?
Part 2	Implementation	
5.	What barriers if any to implementing this service?	
	How could we overcome any barriers?	
6.	What did you see as facilitating the service to be implemented?	
Part 3	Working relationship	
7.	What is your view of your working relationship with the PIP?	• Good relationship areas<br>• Difficult relationship areas
	How can your working relationship with the PIP be improved?	
8.	What is your view of your staffs' relationship with the PIP?	• Good relationship areas<br>• Difficult relationship areas
	How can their working relationship with the PIP be improved?	
9.	How has your working relationship with the PIP affected your relationship with your patients?	• New contributions<br>New problems
10.	Where did communication issues arise between you and the other team members involved in the PIP service, and what were they?	• PIP<br>• Community Pharmacist<br>• Primary care Pharmacist<br>• District Nurse<br>• GP Practice
11.	How do you think communication issues may have affected patient care?	• Examples
Part 4	Acceptability	
12.	What aspects of the PIP service went well from your perspective?	• Time commitment<br>• Paper work<br>• Issues raised/resolved
13.	What aspects of the PIP service went less well from your perspective?	• Time commitment

For peer review only - http://bmjopen.bmj.com/site/about/guidelines.xhtml

	Stem question		Probes / follow ups
			• Paper work<br>• Issues raised/resolved
		How could we improve these?	
14.	What elements of the service did you like best?		
15.	Overall, how satisfied are you with the service provided by the PIP?		
16.	Would you like the service to continue?		• In its current from<br>• In a revised form<br>• Not at all
Part 5	Study procedures		
17.	Recruitment and participation		• Acceptability for residents with/without capacity<br>• Time commitment<br>• Paper work
18.	Any final comments		
19.	Thank you for taking part in this interview		

For peer review only - http://bmjopen.bmj.com/site/about/guidelines.xhtml

CHIPPS WP6 QUAL Evaluation: Topic Guide for Care Home Staff

	**Stem question**	**Probes / follow ups**
Part 1	Views of the intervention	
1.	What involvement have you had with the PIP service?	
2.	Overall what are your views of the PIP service?	• New contributions<br>• New problems
	Views on changing skill mix in the primary care team<br>Views on changing skills mix in the care home team	• Diversity in team(s)<br>• Challenges<br>• Issues in managing
3.	How has the PIP service impacted on your work load? (positively/negatively)	• Time<br>• Referrals<br>• Tests<br>• Medication changes<br>• Medication reviews<br>• Repeat prescriptions<br>• Medication administration processes<br>• Record keeping<br>• Medication storage<br>• Interactions with GPs<br>• Interaction with community pharmacist
4.	In what ways has the PIP service affected patient care? positively/negatively)	• Examples<br>• Acceptability for patients without capacity?
5.	Have you received any additional training since the introduction of the PIP service?	• Impact?
Part 2	Implementation	

For peer review only - http://bmjopen.bmj.com/site/about/guidelines.xhtml

	**Stem question**	**Probes / follow ups**
6.	What barriers if any to implementing this service?	
	How could we overcome any barriers?	
7.	What did you see as facilitating the service to be implemented?	
Part 3	Working relationship	
8.	What is your view of your working relationship with the PIP?	• Good relationship areas<br>• Difficult relationship areas
9.	*How can your working relationship with the PIP be improved?*	
10.	How has your working relationship with the PIP affected your relationship with your patients?	• New contributions<br>New problems
11.	Where did communication issues arise between you and the other team members involved in the PIP service, and what were they?	• PIP<br>• Community Pharmacist<br>• Primary care Pharmacist<br>• District Nurse<br>• GP Practice
12.	How do you think communication may have affected patient care? (positively/ negatively)	• Examples
Part 4.	Acceptability	
13.	What aspects of the PIP service went well from your perspective?	• Time commitment<br>• Paper work<br>• Issues raised/resolved
14.	What aspects of the PIP service went less well from your perspective?	• Time commitment<br>• Paper work<br>• Issues raised/resolved
	How could we improve these?	
15.	What elements of the service did you like best?	• Patient care

For peer review only - http://bmjopen.bmj.com/site/about/guidelines.xhtml

For peer review only

	Stem question	Probes / follow ups
16.	Overall, how satisfied are you with the service provided by the PIP?	
17.	Would you like the service to continue?	• In its current from<br>• In a revised form<br>• Not at all
18.	Any final comments?	
19.	Thank you for taking part in this interview	

For peer review only

For peer review only - http://bmjopen.bmj.com/site/about/guidelines.xhtml

CHIPPS WP6 QUAL Evaluation: Topic guide for residents or relatives

	**Stem question Resident/relatives**	**Probes / follow ups**
Part 1 Awareness of the intervention		
1.	Overall have you noticed any changes in your care/ the care of your relative/friend since the introduction of the PIP service?	
	What changes have you noticed?	• Good/Bad<br>• Examples
Part 2 Implementation		
2.	Before the PIP service started, what did you think about the information you received describing the new service?	• Relevant to you<br>• Amount of detail<br>• Understandable
3.	Given information you had before the PIP service started, how far was the service what you thought it would be?	• Examples
Part 3 Relationship with PIP		
4.	How did you get on with your PIP?	• Good relationship areas<br>• Difficult relationship areas
	How could your relationship with the PIP be improved?	
5.	How easy did you find it to talk to the PIP?	• Examples
6.	How do you think your relationship with the PIP affect your care/ your relative/friends care?	• Examples
Part 4 Acceptability		
7.	What aspects of the PIP service went well from your perspective?	• Examples
8.	What aspects of the PIP service went less well from your perspective?	• Examples
9.	Is there anything we could improve the overall service?	• Examples
10.	Overall how satisfied are you with the service provided by the PIP?	

For peer review only

For peer review only - http://bmjopen.bmj.com/site/about/guidelines.xhtml

Part 5	Study procedures	
16.	Recruitment and participation	• Acceptability for residents without capacity
11.	Any final comments?	
12.	Thank you for taking part in this interview	

End document

For peer review only

For peer review only - http://bmjopen.bmj.com/site/about/guidelines.xhtml

Supplementary file 3 Coding Framework

code	Number participants	Number times coded
1 Implementation	10	30
list of services offered by PIP	5	7
care home study procedure	8	23
GP study procedures	6	7
2 Mechanisms of impact	7	46
medication changes made	13	33
bio chemical monitoring	8	9
non-patient facing activities	8	25
completing PCP	5	15
tasking items to GP	4	4
liaising with community pharmacist about scripts	7	14
liaising with primary care practice pharmacy staff	4	6
evidence of GP tasking items to PIP	4	10
recruiting residents	3	4
authorising repeat prescription	7	13
staff education	8	13
staff training	12	15
review of medication systems	11	18
review of stock	7	8
care home workload due to intervention	7	24
3 Communication	10	62
PIP to GP	9	19
PIP to care home	12	55
PIP to residents and relatives	8	16
PIP to others	10	21
GP to PIP	7	16
Care home to PIP	13	56
resident or relative to PIP	0	0
GP to CH	5	6
4 Outcomes	10	46
case studies of improvement to residents	18	43
case studies where planned change not successful	2	2
reduced drug or stock wastage	3	3
improved ordering system	8	19
adding to multi disciplinary practice	17	44
opinion on intervention	6	32

For peer review only

PIP satisfaction or not	5	11
GP satisfaction or not	7	28
Care home satisfaction or not	10	47
5 intervention safety	4	6
PIP perspective	5	12
GP perspective	7	17
Care home perspective	6	9
resident or relative perspective	0	0
6 contextual factors	13	61
barriers to delivery	18	44
facilitators to delivery	14	42
impact of site factors on delivery	14	34
impact of patient factors on delivery	8	12
GP workload impact	12	27
care home staff awareness	11	30
7 Intervention normalised into routine	13	25
actions taken to ensure intervention works	3	5
narrative of engagement or disengagement	10	18
legacy of intervention	11	20
Future developments	2	4
8 Discussion on professional roles	12	46
9. Geographic differences	6	9
10 Should this type of service be implemented	14	21

For peer review only

Supplementary file 4 Example of text to code 'PIP satisfaction or not'

PIP 1

Reference 1-the one to one contact, the face to face umm for those patients that were able to participate umm you know I think a lot of the time they are happy to see somebody and know there is another person involved in their care, umm a lot of them were great characters and you know it was lovely for building that relationship and then of course there would be the patients where you would be in a relationship with them and then you would be leaving the room and they had forgotten who you were, that was just their you know their conditions, so umm but no it was a positive experience definitely from the patients' perspective and getting to know them.

Reference 2 - I thought the project is really worthwhile umm and I hope that you are able to get all the data and make sense of it all especially with me, umm I, you know I think it's a great project, I have encouraged other people to get involved in it locally umm and you know I know there's a few. -

PIP 2

Reference 1 - part of me, admittedly four hours isn't enough time, sometimes I wish it was more patients because I felt I could have probably made more of an impact and I do wish it was only the one Care Home but other than that.

Reference 2 – I think so with the Primary Care network role, pharmacist's role that is something that each Care Home resident will be getting and should be getting the Pharmacist's review so. Yeah it is, it's utterly definitely of benefit. - §

PIP 6

Reference 1 – Q I'm just wondering if there is a specific example of where you felt that that lack of knowing affected your sort of practice? Response I think it just was the, I think it was just the fact that I never got to the stage where the Home were calling me about things, we never got to that stage

Reference 2 – Q Was there anything that was particularly good about your experience of delivering the service? Response 2 / 3 Definitely, yes, I have been very negative, no I think just the fact that there is a need for it, you know I did pick up on things in review, GPs now are so busy you know particularly when they get given, the way that it happens, that they get given a whole Care Home to look after, they do tend to when, you know once that patient moves into that Care Home the GP will you know do a quick review and all of that to get to know them and their records a bit better but often after that the GP can only ever respond to acute needs and those patients, you know often those, the way it works at the Care Home I was at, that the GP would visit every Thursday, they would have a list of all of the patients that the Care Home wanted them to see so it was often dictated by what the Care Home needs were rather than what the GP, and by that time it was all filled up so the GP you know would follow up on some things but in terms of on-going chronic conditions that's where I feel Pharmacists in Care Homes have an impact and that I saw, so my main things were about trying to reduce pain medication, so we got a few people reduced on some of the Opioid patches and monitoring, not over-monitoring because you know that is totally unnecessary at this stage but the monitoring that is necessary so there was some blood tests that had slipped that were important and de-prescribing other things for different medications like statins that patients don't need so much anymore and the GP I felt was grateful for that because they would love to have

For peer review only

the time to do that I'm sure, but yeah they are working on a, you know firefighting basis whereas this was more of a you know a pro-active approach to it. –

PIP 19

Reference 1 - it was generally positive, there were obviously some things we will perhaps come back to that were sort of challenging in terms of time management and prioritising what I needed to do umm but umm but yes on the whole I think I learnt from it and it was beneficial to the patients I hope. –

PIP 9

Reference 1 - quite good really it kind of built upon the kind of relationships that I had with the Care Home anyway, so I do speak to them quite often on the phone, I deal with queries but it just, it made things better because I had to go and see the Home, I didn't know where the Home was, it was just out there somewhere. I didn't know what the Home was like, I had a picture of it in my head about who the people were who I was dealing with but I didn't know them so I had to go and see them so that was great and then because we knew one another we then kind of shared email addresses and stuff and we made maybe some of the things a bit more twenty-first century, we were emailing one another whereas in the past they were faxing us queries and stuff so, so that's yeah so it was, yeah it was good it kind of pushed us a bit more together to kind of work together.

Reference 2 - can't think of anywhere where it was negative, I can just see it was all positive. Basically we did do a kind of an in-depth six month review of people which I don't think we would have done it had I not been involved in the CHIPPS study you know and there was lots of, because basically what I used it for we kind of took a step back and said 'why is this patient on this drug?' as opposed to 'what's the drug for?' so if they are on drugs and antidepressants you know well anti-depressants they're depressed, but actually what I started doing was going you know 'why were they put on this drug?' and sometimes we didn't know and they were on the drug and I didn't think it was doing any good and I said 'maybe we could maybe halve this or stop this?' so yeah so I can't think of anything, I can't think of anything negative.

Reference 3 - So communication only improved, you know we did move away from them sending faxes to us saying 'and you owe these medicines for various people' to them emailing and you know yeah and it just improved you know and sometimes when I had those emails I was able to reply back to them and say 'I've sorted it out for you now' but I was able to reply to the Pharmacy as well saying 'these things have been sorted, the script is on the way' or whatever and it only improved really, do you know what I mean 'improved'?

Reference 4 - I thought whatever you are going to try and show a difference in, that half my time or double my time was taken filling out paperwork when actually I did do it in half the time but you do not know and I kind of squandered some of that yeah

For peer review only

Table Demographic characteristics of interview participants

Intervention ID Interviewee N=38		General practice			Pharmacist			Care Home		
**Triad**	**Interviewee**	**Location***	**Patient list**	**Previously employed PIP**	**Time Independent Prescriber**	**Previous experience in care homes**	**Number of residents in intervention**	**Type of registration**	**Indices of multiple deprivation***	**Ownership**
**1**	PIP, CH Manager	Northern Ireland Urban	< 10,000	Yes	1 month	No	6	Dual	10	Private
**2**	PIP	England Semirural	≥10,000	Yes	2 months	Yes	20	Dual	8	Private
**3**	GP	England Urban	< 10,000	No	12 months	Yes	16	Residential	6	Local Authority
**4**	PIP	Scotland Urban	≥10,000	No	12 months	No	24	Dual	10	Private
**6**	PIP, GP, CH Manager, CH Staff	England Urban	≥10,000	No	16 months	No	22	Residential	5	Voluntary
**8**	PIP, GP, CH Manager	England Semirural	≥10,000	Yes	18 months	Yes	24	Dual	4	Private
**9**	PIP, CH Manager	England Rural	≥10,000	Yes	18 months	No	21	Residential	6	Private
**11**	PIP, GP, CH Staff	Scotland Semirural	< 10,000	Yes	3 years	No	14	Residential	8	Private
**12**	CH Manager	Northern Ireland Urban	≥10,000	Yes	4 years	Yes	19	Residential	7	Voluntary
**14**	PIP, GP, CH Manager (x2), CH Staff	Scotland Rural	< 10,000	Yes	4 years	No	20	Dual	9	Private
**15**	PIP	Scotland Urban	< 10,000	Yes	6 years	Missing	18	Dual	7	Private
**16**	PIP, GP	England Urban	< 10,000	No	6 years	Yes	20	Dual	1	Private

For peer review only

**17**	PIP	Northern Ireland Urban	Missing	Yes	7 years	Yes	6	Dual	10	Private
**18**	CH Manager	Scotland Urban	< 10,000	Yes	8 years	No	9	Dual	6	Private
**19**	PIP, GP, CH Staff	England Rural	< 10,000	No	9 years	Yes	24	Residential	6	Private
**20**	PIP	Northern Ireland Semirural	Missing	Yes	10 years	Yes	23	Dual	8	Private
**21**	GP, CH Manager (x2), CH Staff	Scotland Urban	< 10,000	Yes	14 years	Yes	21	Dual	10	
**22**	PIP	Scotland Urban	< 10,000	Yes	16 years	Yes	11	Dual	Private	

Key

Location country: urban refers to city GP practice, semirural refers to market town GP practice, rural refers to village GP practice

Indices of Multiple Deprivation: The deciles ranks are nationally calculated by ranking the 32,844 LSOAs in England from most deprived to least deprived and dividing them into 10 equal groups. LSOAs in decile 1 fall within the most deprived 10% of LSOAs nationally and LSOAs in decile 10 fall within the least deprived 10% of LSOAs nationally. NI decile calculated manually from rank

For peer review only

Consolidated criteria for reporting qualitative studies (COREQ): 32-item checklist

Developed from:
Tong A, Sainsbury P, Craig J. Consolidated criteria for reporting qualitative research (COREQ): a 32-item checklist for interviews and focus groups. *International Journal for Quality in Health Care*. 2007. Volume 19, Number 6: pp. 349 – 357

For study A qualitative understanding of outcomes when pharmacist independent prescribers lead on medicine management in older people's care homes.

For peer review only

No. Item	Guide questions/description	Reported on Page #
**Domain 1: Research team and reflexivity**		
Personal Characteristics		
1. Inter viewer/facilitator	Which author/s conducted the interview or focus group?	LB and LD
2. Credentials	What were the researcher's credentials? E.g. PhD, MD	LB has PhD with extensive experience in qualitative methods<br>LD has MA and experience of qualitative data collection and analysis
3. Occupation	What was their occupation at the time of the study?	Researcher
4. Gender	Was the researcher male or female?	Female
5. Experience and training	What experience or training did the researcher have?	LB PhD and 10 years post-doctoral experience<br>LD 5 years post masters experience
Relationship with participants		
6. Relationship established	Was a relationship established prior to study commencement?	.LB had not previous contact with participants<br>LD had had some contact with participants through data collection for RCT
7. Participant knowledge of the interviewer	What did the participants know about the researcher? e.g. personal goals, reasons for doing the research	Participants were aware the study was a follow on from the intervention study and the particular focus was on proactive deprescribing. This was in the participant information sheet

8. Interviewer characteristics	What characteristics were reported about the inter viewer/facilitator? e.g. Bias, assumptions, reasons and interests in the research topic	Reflexive iterative analysis was undertaken the researchers do not work in primary care and have no vested interest in the outcomes page 6
**Domain 2: study design**		
Theoretical framework		
9. Methodological orientation and Theory	What methodological orientation was stated to underpin the study? e.g. grounded theory, discourse analysis, ethnography, phenomenology, content analysis	The reported study uses thematic analysis within a pragmatic design Page 5/6
Participant selection		
10. Sampling	How were participants selected? e.g. purposive, convenience, consecutive, snowball	Purposive sampling of a defined bounded cohort all invited to interview and sampling framework in place Page 5
11. Method of approach	How were participants approached? e.g. face-to-face, telephone, mail, email	Contacted by email Page 5
12. Sample size	How many participants were in the study?	38
13. Non-participation	How many people refused to participate or dropped out? Reasons?	38 recruited from potential sample of 86 although several care home mangers had left the establishment. All you responded to recruitment email were interviewed. Page 6
Setting		
14. Setting of data collection	Where was the data collected? e.g. home, clinic, workplace	Data collected in person and by telephone page 5
15. Presence of non-participants	Was anyone else present besides the participants and researchers?	No
16. Description of sample	What are the important characteristics of the sample? e.g. demographic data, date	Table 1 reports sample characteristics
Data collection		
17. Interview guide	Were questions, prompts, guides provided by the authors? Was it pilot tested?	Not pilot tested but reviewed by experts and PPI and drew on earlier results from feasibility study page 4
18. Repeat interviews	Were repeat inter views carried out? If yes, how many?	NO
19. Audio/visual recording	Did the research use audio or visual recording to collect the data?	Audio recording Page6

For peer review only

20. Field notes	Were field notes made during and/or after the interview or focus group?	Researcher reflexive memos kept Page 6
21. Duration	What was the duration of the inter views or focus group?	30-90 minutes Page 6
22. Data saturation	Was data saturation discussed?	Not explicitly but strengths of sample discussed page 6
23. Transcripts returned	Were transcripts returned to participants for comment and/or correction?	No page 6
**Domain 3: analysis and findings**		
Data analysis		
24. Number of data coders	How many data coders coded the data?	Primarily LB and LD but codes discussed and developed with wider team Page6
25. Description of the coding tree	Did authors provide a description of the coding tree?	No
26. Derivation of themes	Were themes identified in advance or derived from the data?	Inductive analysis Page 6
27. Software	What software, if applicable, was used to manage the data?	NVivo
28. Participant checking	Did participants provide feedback on the findings?	The results were shared with a diverse research team for feedback Page 6
Reporting		
29. Quotations presented	Were participant quotations presented to illustrate the themes/findings? Was each quotation identified? e.g. participant number	Yes in results 9-14
30. Data and findings consistent	Was there consistency between the data presented and the findings?	Yes
31. Clarity of major themes	Were major themes clearly presented in the findings?	Yes
32. Clarity of minor themes	Is there a description of diverse cases or discussion of minor themes?	Yes discussion of minority of responses which indicated concern role of PIP interweaved in results and discussion

For peer review only
